# The *Caulobacter crescentus* phage phiCbK: genomics of a canonical phage

**DOI:** 10.1186/1471-2164-13-542

**Published:** 2012-10-10

**Authors:** Jason J Gill, Joel D Berry, William K Russell, Lauren Lessor, Diego A Escobar-Garcia, Daniel Hernandez, Ashley Kane, Jennifer Keene, Matthew Maddox, Rebecca Martin, Sheba Mohan, Ashlyn M Thorn, David H Russell, Ry Young

**Affiliations:** 1 Center for Phage Technology, 2128 TAMU, Texas A&M University, College Station, Texas, TX, 77843, USA; 2 Dept. of Biochemistry & Biophysics, 2128 TAMU, Texas A&M University, College Station, Texas, TX, 77843, USA; 3 Department of Chemistry, Texas A&M University, College Station, Texas, TX, 77843, USA

**Keywords:** Bacteriophage, Genomics, Caulobacter crescentus, phiCbK

## Abstract

**Background:**

The bacterium *Caulobacter crescentus* is a popular model for the study of cell cycle regulation and senescence. The large prolate siphophage phiCbK has been an important tool in *C. crescentus* biology, and has been studied in its own right as a model for viral morphogenesis. Although a system of some interest, to date little genomic information is available on phiCbK or its relatives.

**Results:**

Five novel phiCbK-like *C. crescentus* bacteriophages, CcrMagneto, CcrSwift, CcrKarma, CcrRogue and CcrColossus, were isolated from the environment. The genomes of phage phiCbK and these five environmental phage isolates were obtained by 454 pyrosequencing. The phiCbK-like phage genomes range in size from 205 kb encoding 318 proteins (phiCbK) to 280 kb encoding 448 proteins (CcrColossus), and were found to contain nonpermuted terminal redundancies of 10 to 17 kb. A novel method of terminal ligation was developed to map genomic termini, which confirmed termini predicted by coverage analysis. This suggests that sequence coverage discontinuities may be useable as predictors of genomic termini in phage genomes. Genomic modules encoding virion morphogenesis, lysis and DNA replication proteins were identified. The phiCbK-like phages were also found to encode a number of intriguing proteins; all contain a clearly T7-like DNA polymerase, and five of the six encode a possible homolog of the *C. crescentus* cell cycle regulator GcrA, which may allow the phage to alter the host cell’s replicative state. The structural proteome of phage phiCbK was determined, identifying the portal, major and minor capsid proteins, the tail tape measure and possible tail fiber proteins. All six phage genomes are clearly related; phiCbK, CcrMagneto, CcrSwift, CcrKarma and CcrRogue form a group related at the DNA level, while CcrColossus is more diverged but retains significant similarity at the protein level.

**Conclusions:**

Due to their lack of any apparent relationship to other described phages, this group is proposed as the founding cohort of a new phage type, the phiCbK-like phages. This work will serve as a foundation for future studies on morphogenesis, infection and phage-host interactions in *C. crescentus*.

## Background

For over 40 years, the alpha-proteobacterium *Caulobacter crescentus* has been an important model organism for the study of bacterial development, physiology and cell cycle biology. *C. crescentus* exhibits a cyclical, dimorphic lifestyle that is atypical among prokaryotes [1,2]. Its sessile form displays an adhesive polar holdfast, or stalk, and this cell type is exclusively capable of DNA replication and cell division. Cell division in *C. crescentus* is asymmetrical, and stalked cells divide to produce motile daughter “swarmer” cells with a single polar flagellum and multiple polar pili. The swarmer cells are unable to divide or replicate their DNA until they shed their flagellum and pili and undergo a physical transformation to the stalked cell morphotype. *C. crescentus* is also unusual in that its DNA replication is closely coordinated with cell division, resulting in the production of a single copy of the bacterial chromosome per division cycle. The regulatory networks that control differentiation and division have been well characterized [3-5].

*Caulobacter* phages were first isolated nearly 50 years ago [6] and have been instrumental as tools for genetic transduction [7,8] and as probes for the presence of cell-cycle specific markers [9-11]. Among the phages of *C. crescentus*, the most extensively studied are large, virulent siphophages with prolate heads, among which phiCbK is the archetype. First reported in 1970 [12], phiCbK has been used as a cell-cycle and morphological indicator [10] because it uses the flagellum and polar pili of the swarmer cell for adsorption [13]. In addition, phiCbK, due to its large size, was one of the earliest phages for which the fine structure of the capsid was determined by electron microscopic image reconstruction [14-16] and was the first instance where the phage head-tail symmetry mismatch was demonstrated [17]. Phage phiCbK and related phages have been shown to have a unique filamentous structure emanating from the top vertex of the capsid [14,18]. This head filament was found to mediate primary attachment of the virion to the flagellum and thus more efficient adsorption to the swarmer cell [18]. However, despite the biological and structural significance of *C. crescentus* phages, little is known about the biology of the phages themselves. Here we report the complete genomes of phage phiCbK and five related *Caulobacter* siphophages. The results are discussed in terms of the unique structure of these phages and the biological imperatives facing phages that infect bacterial species with dimorphic cell types.

## Methods

### Phage isolation and culture

Phage phiCbK and *C. crescentus* strain CB15 were obtained from the Félix d’Hérelle Reference Center for Bacterial Viruses (Université Laval, QC, Canada), and strain CB15 was used for the enrichment and propagation of all phage isolates. *C. crescentus* was cultured at 30°C with aeration in PYE broth (2 g/L peptone (Oxoid), 1 g/L yeast extract (Difco), 0.1 g/L anhydrous MgSO_4_) or PYE agar (PYE broth plus 15 g/L Bacto agar). Phages were propagated and enumerated on PYE plates by the soft agar overlay method [19] using lawns consisting of 4 ml PYE top agar (PYE broth plus 5 g/L Bacto agar) and inoculated with 100 μl of an overnight PYE culture of *C. crescentus* CB15. After plating, lawns were incubated for 42–48 h at 30°C prior to plaque enumeration or harvesting.

Phages other than phiCbK were isolated in early 2010 from surface water samples collected in Bryan and College Station, TX, USA by students enrolled in the Phage Genomics for Undergraduates program run at Texas A&M University. Phages were isolated following culture enrichment or direct concentration methods. In culture enrichment of water samples, 40 ml of filter-sterilized water sample (0.22 μm, Millipore) was added to 10 ml of 5X strength PYE broth, inoculated with 100 μl of a fresh *C. crescentus* CB15 overnight PYE culture and incubated with aeration overnight at 30°C. Enrichment cultures were centrifuged (8,000 x g, 10 min, 4°C), the supernatants filter sterilized (0.22 μm) and plated to lawns of *C. crescentus* CB15, and observed for plaque formation. The direct concentration method was modified from a technique kindly provided by R. Hendrix, University of Pittsburgh (personal communication). Briefly, 1 L of water sample was clarified by filtration through Whatman 597½ paper (Whatman). Five grams of Whatman DE-52 anion exchange resin was added and incubated at 22°C for 30 min with shaking. The resin was collected in a 50 ml centrifuge tube and centrifuged at 2,000 x g, 2 min, 22°C and the supernatant discarded. The resin was washed twice by resuspension in 45 ml wash buffer (25 mM Tris–HCl, pH 7.5, 10 mM NaCl, 5 mM MgSO_4_) followed by centrifugation as above. Phage were eluted from the resin by addition of 30 ml elution buffer (25 mM Tris–HCl, pH 7.5, 600 mM NaCl, 5 mM MgSO_4_) followed by centrifugation as above, and the supernatant was retained and concentrated to a final volume of ~500 μl in a 100 kDa NWCO centrifugal ultrafiltration device (Millipore). This total phage concentrate was then plated to lawns of *C. crescentus* CB15 and observed for plaque formation. Individual plaques were picked and subcultured three times, then propagated to high-titer lysates in soft agar overlays [19].

### Phage DNA preparation and sequencing

Bacteriophage genomic DNA was prepared from 10–20 ml of filter-sterilized, high-titer (> 1 x 10^9^ PFU/ml) phage lysates using a modified form of the Promega Wizard DNA clean-up kit (Promega) as described previously [20]. DNA integrity was verified by running on a 0.8% agarose gel and staining with ethidium bromide and DNA was quantified by band densitometry. Phage genome size was estimated by pulsed field gel electrophoresis (PFGE) analysis of genomic DNA on a 1% agarose gel (Pulsed-Field agarose, BioRad) and comparison to a size marker (Lambda Ladder PFG Marker, New England Biolabs).

Phage genomic DNA was mixed in equimolar amounts and sequenced as MID-labeled pools by 454 pyrosequencing (Roche) at the Emory GRA Genome Center (Emory University, GA, USA). Trimmed FLX Titanium flowgram outputs were assembled using the Newbler assembler version 2.0.01.14 or 2.5.3 (454 Life Sciences) at default settings. Contigs were confirmed to be complete by PCR, using primers that faced off each end of the contigs and sequencing of the resulting products. Phage phiCbK was sequenced to 181-fold average coverage, Colossus to 74-fold coverage, Rogue to 38-fold coverage, Swift to 59-fold coverage, Karma to 291-fold coverage and Magneto to 83-fold coverage. In all cases, the phage genomes produced circular assemblies. Breakpoints in sequencing coverage were determined by manual inspection of contigs in CLC Workbench version 6.2 (CLC bio).

### Terminal labeling of genomic DNA

In order to locate the physical termini of the phiCbK chromosome, whole phage genomic DNA was ligated with a short oligonucleotide tag of known sequence and this product resequenced by pyrosequencing as described above. In principle, the oligo tag will ligate to the physical chromosomal termini and the boundary between the tag sequence and the phage genomic sequence will indicate the original physical ends of the phage chromosome. Genomic DNA of phage phiCbK was end-repaired with the NEBNext End Repair Module (NEB) using 1 μg of DNA and 1 μl of enzyme in a 100 μl reaction according to the manufacturer’s protocol. End-repaired DNA was precipitated by addition of 200 mM NaCl and 3 volumes of ethanol, and resuspended in 20 μl water. A 49 bp dsDNA oligonucleotide (5' - TTACTTACAATCCTTGGCGGTTTTGCTGCGCGCCCATGATGGACTGGAC - 3') was added to the genomic DNA at a 25:1 molar ratio and ligated at 16°C for 18 h with T4 DNA ligase (NEB) in a 50 μl reaction volume. The ligase was heat inactivated (65°C, 10 min), DNA precipitated with NaCl and ethanol as described above, resuspended in 20 μl water and submitted for sequencing by 454 pyrosequencing. All reads from this resequencing run containing the complete 49 bp oligo sequence were extracted from the sequencing output and assembled onto the previously established phiCbK genomic sequence to locate hotspots of tag ligation.

### Genome annotation

Genes were predicted using GeneMark.hmm [21] and Glimmer 3 [22] and gene starts manually edited in Artemis [23]. tRNA genes were predicted by tRNAscan-SE 1.21 [24] and Rfam [25], and Rho-independent terminators predicted by TransTermHP [26]. Proteins were exported and analyzed in batch by BLASTp [27]; protein functional characteristics were predicted by batch analysis of all proteins in InterProScan version 4.7 [28]. Proteins of particular interest were additionally analyzed by HHpred searches against the pdb70_18Aug12 database [29], TMHMM (http://www.cbs.dtu.dk/services/TMHMM), LipoP 1.0 [30], and the EMBOSS package [31]. Intergenomic protein comparisons were conducted via RAST [32] using phiCbK or Colossus as the reference genome and a similarity cutoff of 25%, and variably conserved proteins were grouped by NCBI BLASTClust at settings of L=0.9, S=30. Figures were generated using Circos version 0.55 [33] and DNA Master (cobamide2.bio.pitt.edu/computer.htm).

### Sequence deposition

The names of novel phage isolates were prefixed with Ccr, which is the ReBase species acronym for *C. crescentus* (rebase.neb.com). Completed phage genomes were deposited in GenBank under the following accession numbers: phiCbK, JX100813; CcrMagneto, JX100812; CcrSwift, JX100809; CcrKarma, JX100811; CcrRogue, JX100814; CcrColossus, JX100810. For simplicity, the phage names will be used without the Ccr prefix.

### Proteomic analysis

Phage phiCbK was propagated in 2 L PYE liquid culture at 30°C to a titer of ~10^10^ PFU/ml. Phage lysate was clarified by two rounds of centrifugation (8,000 x g, 10 min, 4°C) and the phage-containing supernatant was digested with 1 μg/ml DNase I and RNase A (Sigma) for 2 h at 22°C. Phage was concentrated by pelleting in a centrifuge (6,000 x g, 18 h, 4°C) and the pellet was resuspended in λ-dil buffer (25 mM Tris–HCl pH 7.5, 100 mM NaCl, 8 mM MgSO_4_). Residual cell debris was removed from the phage suspension by centrifugation (12,000 x g, 15 min, 4°C) and the phage were then banded by isopycnic CsCl gradient ultracentrifugation, dialyzed and concentrated as described previously [34]. The phage preparation was diluted in λ-dil, boiled for 5 min and incubated with 2 μg/ml DNase I for 1 h at 37°C to reduce sample viscosity. Samples were then mixed with Laemmli sample buffer (62.5 mM Tris–HCl pH 6.8, 2% SDS, 10% glycerol, 5% β-mercaptoethanol, 0.001% bromophenol blue), boiled for 5 min and the equivalent of 1 x 10^10^ to 5 x 10^10^ PFU/lane were run on a 10% Tris-glycine SDS-PAGE gel by standard methods [35]. Bands were visualized with Coomassie blue, and the entire gel lane with the exception of the dye front was segmented based on band boundaries. All gel segments, containing visible bands or unstained inter-band regions, were subjected to proteomic analysis as described previously [34]. Alternatively, digested samples were injected into a Waters nanoACQUITY system for UPLC separation of peptic peptides. Separation was achieved after the peptides were trapped and desalted on a VanGuard Pre-Column trap (2.1 × 5 mm, ACQUITY UPLC BEH C18, 1.7 μm) for 3 min. Peptides were eluted from the trap using an 2%–40% linear gradient of acetonitrile over 32 min at a flow rate of 0.40 μl/min and were separated using an ACQUITY UPLC BEH C18 1.7 μm 1.0 × 100 mm column. Peptides that were produced from the enzymatic cleavage were identified from the using Waters MS^E^ technology on a Waters Synapte G2 instrument and ProteinLynx Global Server (PLGS) searches of a customized database.

### Transmission electron microscopy

Phages were prepared for microscopy by the Valentine method [36] and stained with 2% (w/v) uranyl acetate. Grids were viewed in a JEOL 1200 EX transmission electron microscope under 100 kV accelerating voltage. Five virions of each phage were measured and these data used to calculate mean dimensions.

## Results and discussion

### Phage and genome characteristics

**Figure 1:**
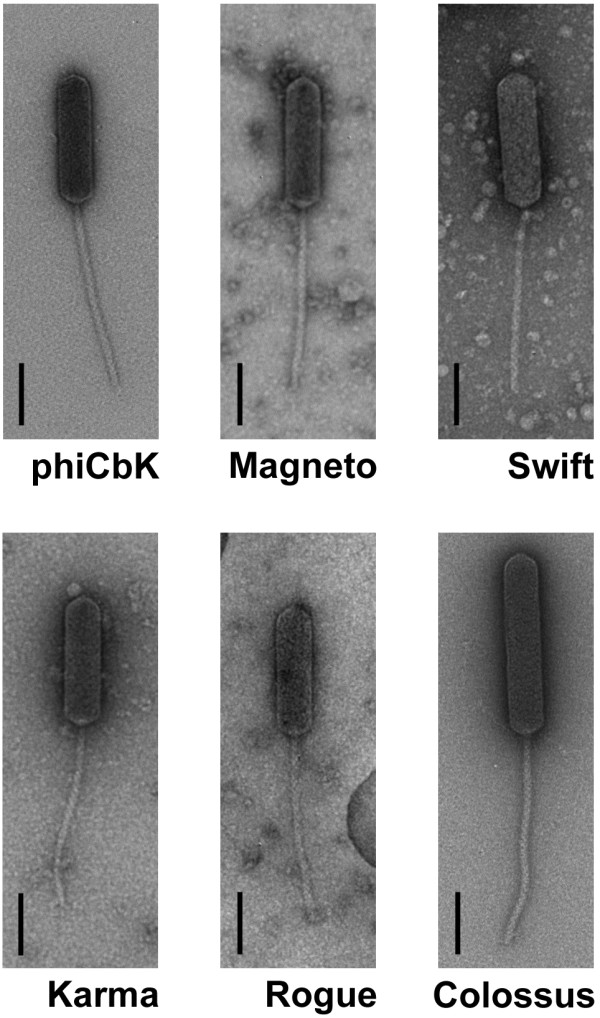
**Negative-stain transmission electron micrographs of the *C. crescentus *phage phiCbK and five phiCbK-like phages.** All five exhibit *Siphoviridae* morphology and prolate heads. Scale bars are 100 nm.

Phages Magneto, Swift, Karma, Rogue and Colossus were isolated from surface waters; Magneto, Swift, Rogue and Colossus were obtained by the culture enrichment method, and phage Karma was isolated by the direct concentration method. Electron microscopic analysis of these phages revealed that all five possessed similar *Siphoviridae* morphology to the previously described phiCbK, with long, non-contractile tails and large prolate heads of varying lengths (Figure 1). The phage phiCbK head measured 205 nm long and 56 nm wide with a tail length of 300 nm, dimensions which are in good agreement with previously reported measurements [14,15,18]. Phage dimensions are summarized in Table 1; all of the phages exhibited similar dimensions to that of phage phiCbK except for Colossus, which had a considerably longer head and slightly longer tail than the other phages.

**Table 1:** **Summary of physical dimensions and genomic characteristics of six phiCbK-like bacteriophages of *C. crescentus***

**Feature**	**Phage isolate**					
	**phiCbk**	**Magneto**	**Swift**	**Kanma**	**Rogue**	**Colossus**
Head length (nm)^a^	205 (±2)	211 (±3)	219 (±6)	205 (±3)	205 (±9)	292 (±4)
Head width (nm)^a^	56 (±2)	58 (±2)	63 (±2)	61 (±2)	60 (±1)	65 (±5)
Tail length (nm)^a^	300 (±8)	293 (±5)	295 (±4)	314 (±10)	319 (±12)	336 (±9)
Unit genome (bp)	205,423	208,983	209,245	211,574	213,399	279,967
Terminal repeat (bp)	10,287	9,946	9,971	10,254	10,321	~16,700^b^
GC content	66.2%	66.6%	66.1%	66.2%	66.6%	62.2%
No. protein-coding genes	318	328	325	333	331	448
No. unique genes^c^	1	3	2	4	61	307
No. tRNA genes	26	27	27	26	23	28

^a^Phage physical dimensions are the means of measurements of five virions, bracketed values after each dimension indicate standard deviations.

^b^One boundary of the terminal repeat in Colossus is indistinct, thus the repeat length is approximate.

^c^Protein-coding genes that were not detected in any of the other 5 phiCbK-like phage genomes sequenced.

**Figure 2:**
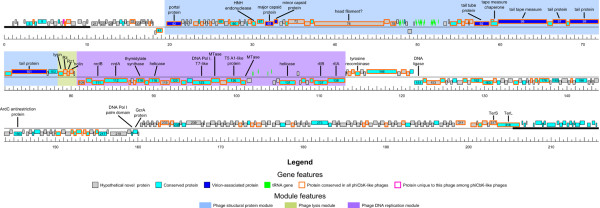
**Genomic map of *C. crescentus *phage phiCbK.** Predicted genes are represented by boxes above and below the black line; boxes above the line are genes encoded on the forward strand, those below the line are on the reverse strand. Segments of heavier black line at each end of the genome represent the 10.3 kb terminal repeats present in the genome. Gene features (conserved, unique, hypothetical novel and virion-associated proteins; tRNA genes) and genome modules (assembly, lysis and DNA replication) are color-coded according to the legend below the figure. Selected genes and gene modules are annotated based on predicted function, as documented in Table S1 and the text. The ruler below the genomes indicates scale in kb.

**Figure 3:**
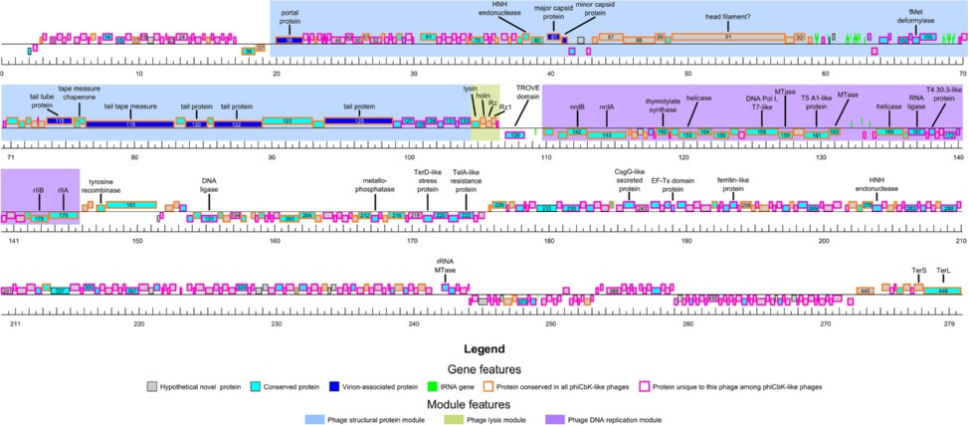
**Genomic map of *C. crescentus *phage Colossus.** Predicted genes are represented by boxes above and below the black line; boxes above the line are genes encoded on the forward strand, those below the line are on the reverse strand. Gene features (conserved, unique, hypothetical novel and virion-associated proteins; tRNA genes) and genome modules (assembly, lysis and DNA replication) are color-coded according to the legend below the figure. Selected genes and gene modules are annotated based on predicted function, as documented in Table S6 and the text. The ruler below the genomes indicates scale in kb.

The genomes of these six phages were sequenced to completion by 454 pyrosequencing followed by manual closure, representing a total combined DNA sequence of 1.33 Mb. The general characteristics of the phage genomes are summarized in Table 1, complete annotations and supporting evidence are provided as supplementary data in Additional file 1: Tables S1 - S6. The unit genomes of these phages ranged from 205.4 kb encoding 318 protein-coding genes (phiCbK) to 280 kb encoding 448 protein-coding genes (Colossus), with terminal direct repeats of ~10 kb in all phages except Colossus, in which the terminal redundancy appeared to be closer to ~17 kb. Three major modules can be identified in these genomes (Figure 2, Figure 3). The phage structural protein module begins with the gene encoding the portal protein (gp42 in phiCbK, gp38 in Colossus) at an apparent divergent transcription site and extends to slightly after a tail protein gene on the plus strand. A lysis cassette, encoding an endolysin, a holin and a two-component spanin protein immediately follows the structural module on the same strand. A DNA metabolism and replication module is encoded on the minus strand, beginning at another divergent transcription site with an *rIIA*/*rIIB* gene pair (gp138/137 in phiCbK, gp179/178 in Colossus) and extending to the end of the lysis cassette. These genomic features are described in greater detail in the following sections.

The GC content of the genomes were all close to the 67.2% GC content of *C. crescentus* CB15. Each phage also encodes a large number of tRNA genes, ranging from 23 in Rogue to 28 found in Colossus; Rfam analysis did not detect any functional RNA elements other than tRNAs. Each phage encodes tRNAs specific for 13 to 16 amino acids; Thr and Tyr tRNAs are not found in any phage, Asn and His are found only in Colossus, and Ala is found only in Magneto, Swift and Colossus. Phage phiCbK is the only phage to lack an Arg tRNA, while Rogue lacks Asp and Ser. Codon usage was analyzed for phages phiCbK and Colossus, and this analysis suggests the phage-encoded tRNAs may aid the phage by supplying additional tRNAs specific for codons most commonly used by the phage. In the case of phiCbK, the 23 codons represented by phage-encoded tRNAs specified 58% of the total amino acid residues encoded by phage proteins; in Colossus the 24 codons with corresponding phage tRNAs specified 50% of residues.

### Relationships of the phiCbK-like phages

**Figure 4:**
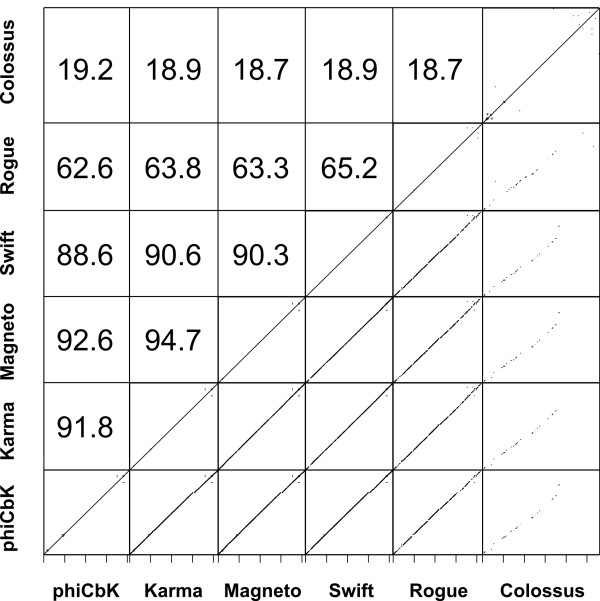
**DNA sequence relatedness of six phiCbK-like phages.** Upper section: pairwise percent DNA sequence identities between all six phages, as determined by BlastN analysis [37] followed by multiplication of the mean percent identity of matched segments by the percent length of the genomes matched. Lower section: dotplots visually representing DNA sequence homology between phages. For clarity, terminal repeat regions were removed from the DNA sequences prior to analysis.

**Figure 5:**
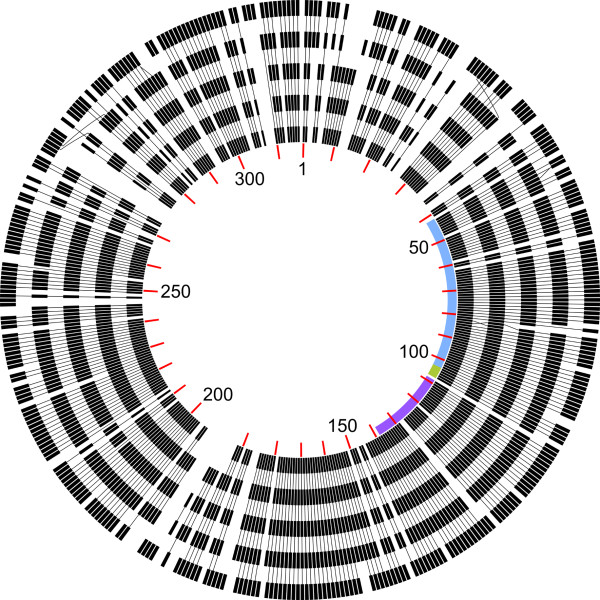
**Non-proportional synteny map showing the relationships between related *C. crescentus *phages at the protein level.** The black blocks represent protein-coding genes in the order they appear in each phage genome, starting with gp1 at the top and running clockwise, with red tick marks indicating 10-gene intervals in phage phiCbK. Black lines connecting blocks indicate similarity of proteins between phages. From innermost to outermost, tracks represent phages phiCbK, Karma, Magneto, Swift and Rogue. Blue, green and purple arcs on the inside track indicate the boundaries of the phage morphogenesis, lysis and DNA replication modules, respectively. Terminal repeat regions were excluded from this figure for clarity.

**Figure 6:**
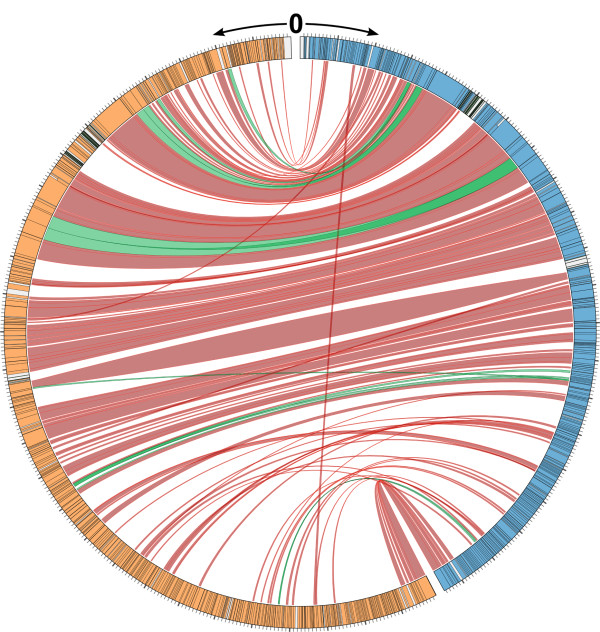
**Map showing the relationship between *C. crescentus *phages phiCbK and Colossus at the protein level.** Both genomes start at position 0 at the top of the figure and move down, with Colossus in orange on the left and phiCbK in blue on the right. Black bands in each track denote the boundaries of protein-coding genes, grey areas represent non-coding regions. Grey tick marks on the outside edge represent 1 kb of sequence, with heavier ticks representing 10 kb. Proteins present in both phages are connected by red ribbons between the two genomes; green ribbons mark proteins with more than one homolog in the other phage, suggesting gene duplications. For clarity, terminal repeat regions were excluded from this figure.

Based on analyses of DNA similarity (Figure 4), phages phiCbK, Karma, Magneto and Swift were found to form a tightly cohesive group (88.6% – 94.7% identity), with Rogue slightly less related (62.6% identity with phiCbK) and Colossus more distantly related (19.2% identity with phiCbK). Because of these relationships, the phage genomes will be discussed in the context of either phiCbK or Colossus, as phiCbK is representative of phages Karma, Magneto, Swift and Rogue. Comparison of the more closely related phages phiCbK, Karma, Magneto, Swift and Rogue at the protein level (Figure 5) shows that these phages are largely syntenic, with few gene translocations. Gene insertions and deletions generally occur singly or as discrete groups of 2–8 genes. Phages Karma, Magneto, Swift and Rogue contain a cluster of 7–8 additional genes inserted between phiCbK genes *192* and *193*. Aside from the presence of a putative DNA-binding protein (gp198 in phage Karma and conserved in phages Magneto, Swift and Rogue), the function of this region is unknown. Due to its more distant relationship to the other phages, the protein-coding genes of Colossus were compared to phage phiCbK separately. Of 448 predicted proteins in phage Colossus, 307 (68%) do not have any homologs in the other five phiCbK-like phages. Nevertheless, as shown in Figure 6, Colossus is still largely syntenic with phiCbK. The regions of greatest protein conservation occur in the central portion of the genomes, which contain the predicted structural and DNA replication proteins (Figure 3). Large sections at the left and right ends of the genomes, primarily containing proteins of unknown function, are less well conserved. These sections contain the majority of gene insertions and deletions, and also several apparent gene translocations and duplications (Figure 6). Phage phiCbK genes *68, 99* and *176* appear to be directly duplicated in Colossus, and Colossus genes *212* and *346* are duplicated in phiCbK. In the case of phiCbK gp68 one of the duplicates, Colossus gene *36,* is also significantly diverged and translocated to a position ~21 kb upstream of its paralog, the Colossus major capsid protein gene *81* (see below).

While these phages are related to one another, a striking feature of the phiCbK-like phages is the lack of any meaningful relationship to other described organisms, including other phages. In the case of phage phiCbK, 239 of its 318 predicted proteins, or 75%, have no matches (E value >1 x 10^-5^) to proteins in the NCBI nr database. Similarly, in phage Colossus, 310 proteins (69.2%) do not have any detectable homologs in the nr database. The phage most closely related to phiCbK is PhiJL001 (YP_224010), a slightly prolate siphophage that infects an uncharacterized marine *Alphaproteobacterium* strain [38]. This relationship however is extremely distant, as even in this case only seven phiCbK proteins, (gp42, gp96, gp97, gp98, gp99, gp118, gp126) mostly located in the phage tail structural region, are detectably related to PhiJL001 proteins, with 14.3 – 40.3% identity. These proteins in themselves do not appear to form a cohesive evolutionary module; for example the PhiJL001 portal homolog gp60 (YP_223984) is most closely related to proteins found in *Bordetella* genomes, while the tail protein homolog gp84 (YP_224008) has a homolog located in *Polymorphum gilvum*. Three of these proteins, gp96, gp97 and gp99, are related to gene transfer agent proteins orfg12 (ABK27260), orfg13 (ABK27261) and orfg15 (ABK27263) of *Rhodobacter capsulatus*[39] (with 42.3%, 17.2% and 15.9% similarity, respectively), suggesting some very distant relationship between the phiCbK-like phages and these phage-like gene transfer agents.

Due to frequent horizontal gene transfer events, the concept of biological species or hierarchical lineages in the classical Linnaean sense are difficult to apply to phages [40,41]. The model of “phage type” has been proposed, which groups phages based on shared features of genome organization and function [42]. Protein or DNA sequence relationships have been used to organize phages into groups that appear to have biological meaning (e.g., [43-45]), however in all of these cases the enormous diversity of phages results in numerous phage groups with no obvious evolutionary linkage to each other. While the phiCbK-like phages lack obvious relationships to any other described organisms, they are obviously related to each other; 119 proteins were found to be conserved in all six genomes. These conserved proteins form syntenic blocks and are primarily located in the phage structural, lysis and DNA replication modules (Figure 2, Figure 3), suggesting these constitute the core genes of the phiCbK phage type. Given the lack of any clear relationship to any other described phages, we propose phage phiCbK as the founder of a novel phage type, the phiCbK-like phages, which includes phages Magneto, Swift, Karma, Rogue and Colossus as its inaugural members, with the latter perhaps the founder of a sub-type.

### Determination of genomic termini

Following closure, the phage phiCbK genome produced a circular assembly of 205,423 bp. However, the sequence contigs assembled from pyrosequencing reads possessed a 10,287 bp region of significantly higher sequence coverage than the rest of the genome. At the boundaries of this region, sequence coverage abruptly transitioned from approximately 170-fold to over 360-fold between two base positions, and coverage of this region was on average 2.2-fold greater than the rest of the sequence. This suggested that the high coverage region was a large terminal repeat of ~10 kbp, as found in the classical coliphage T5 [46]. To test this notion, the phiCbK genomic DNA was subjected to terminal labeling analysis to determine the physical ends of the phage genome. This procedure retrieved 52 pyrosequencing reads containing the complete oligo tag sequence, of which 41 assembled to the established phiCbK genome. Fifteen of these 41 reads assembled to two distinct loci on the phiCbK genome, with the boundaries of the oligo sequence and the phage genomic sequence corresponding exactly to the boundaries of the high-coverage region identified in the phiCbK assembly. The remaining 26 sequencing reads assembled to various loci across the genome, which we interpret as tag ligation to double-stranded DNA breaks generated during the DNA isolation procedure, or possibly termini generated by aberrant packaging events. These experimental data were interpreted as a confirmation of the presence and location of long direct terminal repeats in the phiCbK genome, and the genome was therefore reopened accordingly as shown in Figure 2.

**Figure 7:**
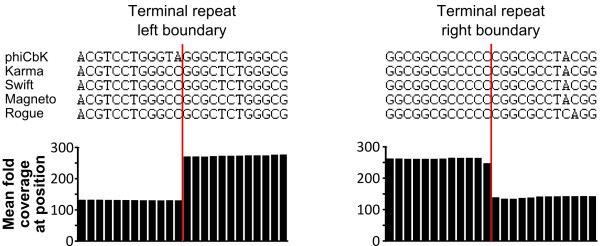
**The left and right genomic terminal repeat boundaries of phage phiCbK and four phiCbK-like phages.** Terminal boundaries are indicated by the vertical red lines. Above: aligned DNA sequences 12 bp up- and downstream of each terminus are shown; alignments show that the experimentally confirmed boundary sequences of phiCbK are nearly identical to those found in the other four close phiCbK-like relatives. Below: average fold coverage at each base position for all five genomic sequences; note the coverage within the terminal repeats is approximately twofold greater than the surrounding genome, and the breakpoints are identical.

The left terminus of the physical genome lies within codon 652 of the predicted terminase large subunit gene, while the right terminus resides in a predicted non-coding region between genes *20* and *21*, resulting in a 10,287 bp direct repeat. Phages Swift, Magneto, Karma and Rogue exhibited similar abrupt discontinuities in sequencing coverage to that of phage phiCbK, indicating that these phages possess direct terminal repeats similar to that of phiCbK. In all five cases these coverage transition boundaries were of identical or nearly identical nucleotide sequence to that found in phiCbK (Figure 7), with the left boundary in all cases lying within codon 652 of the large terminase gene, and the right boundary within an intergenic region ~10 kb downstream. Given these similarities, the genomes of phages Swift, Magneto, Karma and Rogue were also reopened and annotated to reflect the presence of these repeats. The sizes of the terminal repeat regions in these phage genomes are summarized in Table 1.

Like the other phiCbK-like phages, Colossus exhibited an abrupt transition at the left end of a high-coverage region, but instead of an equally abrupt reduction at the end of a discrete length of sequence, a more gradual transition was observed, spanning ~ 200 bp from high to normal coverage on the right end. In this case the left boundary is in a non-coding region slightly downstream of the large terminase gene, and the right boundary lies within gene *34*, some 16,700 bp downstream. Thus, Colossus possesses a non-permuted terminal repeat like the other phiCbK-like phages, marking the initiation site for genome packaging. However, unlike the more closely related phiCbK-like phages, where packaging terminates at the end of the terminal repeat, genome packaging in Colossus appears to have a more imprecise termination and cleavage mechanism.

While all *Caudovirales* phages package their DNA into the capsid as a linear molecule, the nature of the genomic termini can be markedly different between phage types [47]. Phage genomes may have non-permuted termini with short 3’ or 5’ overhangs (like phage lambda), circularly permuted terminal redundancies (like phage T4), or non-permuted direct terminal repeats which can be either short (like phage T7) or long (like phage T5). Some phages, like Mu, have host DNA at their termini, and the phi29-like phages have a covalently linked protein. Characterizing these diverse modalities for DNA packaging is crucial for understanding the biology of phages. In addition, there is practical significance, in that these mechanisms differ greatly in supporting generalized transduction, a feature of great utility for bacterial genetics but highly undesirable in phages being considered for therapeutic use [48]. With the advent of next-generation DNA sequencing, a rate-limiting step in phage genomics is now the determination of the genomic termini, which must still largely be determined experimentally. This is especially true of phages with non-permuted terminal repeats: during sequence assembly, reads from each identical repeat are usually collapsed into a single region in the middle of the assembled contig, resulting in what can appear to be a circularly permuted genome. Furthermore, determination of the true boundaries of direct repeats by traditional methods of restriction mapping and direct sequencing is laborious and time consuming [47]. Here, simple quantification of coverage depth in the assembled contigs revealed the boundaries of non-permuted terminal repeats in these genomes. Moreover, the terminal ligation method developed here confirmed these repeats and the position of the genomic termini in the phiCbK genome, and the close DNA sequence homology and similar coverage patterns (Figure 7) allowed high-confidence assignment of the termini in four of the other five phages. Deep-sequencing coverage has been used to determine copy number variation in other genomic contexts (recently reviewed in [49]), but the small size and low complexity of phage genomes allows for manual inspection of coverage patterns. The terminal ligation method used here is similar in principle to a previously described method based on cloning of restriction fragments following linker ligation [50]. The use of next-generation sequencing, however, removes the limitations imposed by restriction digestion of the DNA, and thus should be applicable to mapping all forms of genomic termini. In principle this method can be run in parallel with the phage *de novo* sequencing run to obtain terminus information simultaneously with the genomic sequence.

### Terminase and DNA packaging

The genes encoding the large subunit terminase (TerL) proteins of the phiCbK-like phages are located at the far right end of the genomes (Figure 2, Figure 3). In all phages except Colossus, the TerL gene overlaps with the terminal repeat, generating an unusual architecture in which the C-terminal fragment of *terL* is duplicated in each repeat. The novel genes immediately upstream of *terL* (gene *317* in phiCbK and *447* in Colossus) are predicted to encode small subunit terminase (TerS) proteins, based on their HHpred similarity to the TerS-based PDB family 3zqp_A (91.6% probability in phiCbK), and their position and size relative to *terL*. These proteins are conserved within the phiCbK-like phages but otherwise do not have any homologs in the NCBI database detectable by BLASTp. Phage TerS proteins are generally less conserved and more difficult to predict by simple sequence homology than TerL proteins.

The phiCbK-like TerL proteins contain an intein in the N-terminal portion of the protein, based on the detection of conserved intein domains by InterProScan, including a Hint domain (IPR003587) and intein splice site (IPR006141). Inteins are self-splicing elements that post-translationally catalyze their own excision from the completed protein. Based on alignments of the phiCbK TerL with its non-intein containing homologs, the putative intein appears to reside between K130 and S472, residues which are adjacent and absolutely conserved in 50 related TerL proteins (E = 10^-28^ to 10^-43^ vs the nr database). The intein sequence itself is predicted to contain the 341 residues inclusive of P131 to N471, resulting in a 568 aa functional TerL protein following splicing; these boundaries are annotated in the phage genome records. The C-terminal boundary of the intein, S472, immediately follows the conserved His-Asn residues often associated with the C-terminal intein-extein boundary [51]. While the N-terminal intein boundary does not possess the Ser or Cys residue involved in typical N-terminal intein cleavage, functional inteins with a Pro at this position have been described [52]. Interestingly, the N-terminal region of the mature TerL protein is not related to other TerL proteins, including those that share similarity in the C-terminal region. The N-terminal domain of the phiCbK-like TerL may be the result of a recombinational event that swapped this portion of the protein for a different domain, perhaps using the intein sequence to provide homologous DNA for recombination.

The TerL proteins of phiCbK, Swift, Magneto and Karma share 100% identity in amino acid sequence. The TerL protein of Rogue is 87% similar, while Colossus is only 55% similar to that of phiCbK. Previous bioinformatic analysis of bacteriophage large subunit terminase proteins has shown that TerL alleles can be grouped into families that correlate to the DNA packaging style of the organism [53]. A mature version of these TerL proteins, with the predicted inteins excised, was used for BLAST analysis and alignment. Among the TerL proteins of phages with known packaging types, the phiCbK TerL protein is most similar to that of phages AaΦ23 (NP_852753, 25.6% similarity) and PY100 (CAJ28416, 24.4% similarity). Phage AaΦ23 has been shown to package DNA that is circularly permuted and terminally redundant by 3.5% of the phage genome [54], and phage PY100 packages its DNA by a headful mechanism initiated at a defined *pac* site analogous to the mechanism employed by phage P22 [55]. Given the low protein similarity of the phiCbK TerL to even its closest relatives, we propose that the TerL proteins of phages phiCbK, Magneto, Swift, Karma and Rogue constitute a novel TerL class that packages phage DNA via a mechanism of long direct terminal repeats in a manner similar to that of phages T5 or SPO1 [46,56]. The TerL homolog of phage Colossus is distinctly diverged from the phiCbK TerL and appears to use a slightly altered packaging mechanism, based on analysis of its genomic termini as described above.

### DNA replication

The central portion of the phiCbK genome contains a region of genes encoded predominantly on the minus strand, beginning approximately at position 113,200 and ending at 80,700, encompassing genes *109* through *138*. In phage Colossus, this region spans position 146,000 to 109,000, including Colossus genes *140* through *179*. This module appears to contain phage DNA metabolism and replication functions, including predicted aerobic ribonucleoside reductase subunits (phiCbK gp111 and gp112), thymidylate synthase (phiCbK gp116), a RecD-like ExoV helicase (phiCbK gp118), a DNA Pol III-like ribonuclease (phiCbK gp121) and a T7-like DNA polymerase (phiCbK gp123). The ribonucleoside reductase alpha subunit (phiCbK gp112) contains an intein that spans C278 to N584 inclusive, featuring the commonly conserved Cys residue at its N terminus and a relatively uncommon C-terminal Gly-Gln [52], resulting in a mature 629 amino acid protein. This intein is also present in the Colossus homolog gp143. The only other organism containing this intein feature is invertebrate iridescent virus 6 (NP_149548). While the alpha subunit possesses significant similarity to other proteins in the database (e.g., to the *C. crescentus* CB15 homolog NP_422286, 24.8% identity), the ribonucleoside reductase beta subunit (gp112) exhibits virtually no similarity to previously identified proteins. The beta subunit also contains a C-terminal thioredoxin-like domain (IPR012336, IPR002109), a domain architecture found only in the ribonucleoside reductase of *Vibrio cholerae* phage ICP1 (YP_004251147).

An interesting feature of all six phiCbK-like phages is the presence of a DNA polymerase, gp123 in phiCbK and gp158 in Colossus, which closely resembles that of coliphage T7 and members of the T7-like phage superfamily. Among the most closely related proteins to phiCbK gp123 is the DNA polymerase gp5 of phage T7 itself (NP_041982, E = 4 x 10^-109^, 35.1% identity) which can be aligned to phiCbK gp123 over its entire length. This relationship suggests that phiCbK-like phages may replicate their DNA in a manner similar to that of phage T7. Coliphage T7 employs a unique mechanism of DNA replication, requiring only four proteins: a DNA polymerase (gp5), a combination helicase-primase (gp4), a single-stranded DNA binding protein (gp2.5), and thioredoxin which serves as DNA polymerase processivity factor and is supplied by the *E. coli* host [57]. Both the phiCbK-like and T7 DNA polymerases possess similar domain architectures, with an N-terminal ribonuclease-like domain (IPR012337) responsible for proofreading and C-terminal DNA polymerase A domain (IPR001098). Unlike other members of the polA family such as the *E. coli* DNA pol I, which are low processivity enzymes involved in DNA repair, T7 gp5 is responsible for replication of the phage chromosome with an associated high processivity of thousands of bp per event [58]. The high processivity of T7 gp5 is conferred in large part by its association with host thioredoxin [58,59]; the thioredoxin-binding loop of T7 gp5 (residues 258–333) [59] is present but extended by 11 aa in phiCbK gp123, thus the phiCbK-like homologs may establish similar interactions with another protein to enhance their processivity. Phage phiCbK possesses a DNA helicase (gp131), but this protein does not appear to contain a DNA primase domain like that of T7 and is not related to T7 gp4 at the primary structure level. A single-stranded DNA binding protein was not detected in the genome of phage phiCbK or its relatives. If these phiCbK-like phages replicate their DNA in a manner similar to that of the T7-like phages, they may use host proteins to complete the DNA replication complex. To our knowledge, this is the first instance of a T7-like DNA polymerase appearing in a phage outside of the T7 superfamily.

PhiCbK gene *126* (Colossus *161*) encodes a homolog of the coliphage T5 A1 protein (YP_006832, 34.2% similarity, E ~ 10^-89^), located transcriptionally slightly upstream of the predicted DNA polymerase (Figure 2 and Figure 3). This is the second time that an A1 homolog has been detected outside of the known T5-like phages, with the only other instance in the genome of phiJL001 (YP_223929, 31.8% similarity). In T5, the A1 protein is transcribed in the pre-early stage of infection as one of the eight genes of the first-step-transfer (FST) DNA, which corresponds to the left large terminal repeat. A1 mutants are defective in multiple processes, including failure to degrade host DNA and to shut off T5 pre-early gene expression [60], and to accomplish the transfer of the rest of the T5 genome into the cell [61]. Oddly, A1 fractionates with the membrane fraction after treatment with RNase and DNase [62]. It also co-immunoprecipitates with host RNA polymerase separated in glycerol gradients, and A1-bound RNA polymerase molecules have altered transcription patterns on T5 DNA [63]. The simplest notion is that A1 is a transcription factor that also interacts with a membrane protein. In support of this notion, the only T5 FST gene that encodes a predicted membrane protein is adjacent to *A1*. In this perspective, the various diverse phenotypes of *A1* mutants are derived from a failure to alter the promoter specificity during pre-early gene expression. Because of their location in the phiCbK-like phages within the DNA replication module, it seems unlikely that these A1 homologs would play a role in DNA degradation, and their function may be to alter phiCbK gene expression via interaction with the host RNA polymerase. This function is supported by an N-terminal similarity of these proteins to PDB family 1g2h_A (94.0% probability in phiCbK) as detected by HHpred; 1g2h is based on the DNA-binding domain of the TyrR transcription factor of *Haemophilus influenzae*.

Tyrosine recombinase proteins were identified in all six phiCbK-like phages, in phage phiCbK this protein is gp143. Site-specific tyrosine recombinases like gp143 are often associated with prophage integration. However, as there are no reports of phage phiCbK exhibiting temperate behavior, it is most likely that the gp143 recombinase instead plays some role in the resolution of replicative intermediates or recombinational multimers of the phage chromosome. Tyrosine recombinases of this type are well known to be involved in chromosomal segregation, as with *E. coli* XerC and XerD [64] or the resolution of recombinational dimers, as with the Cre protein of coliphage P1 [65]. At 208 aa, phiCbK gp143 is also considerably smaller than other well-studied tyrosine recombinases such as phage lambda Int (NP_040609, 356 aa) or P1 Cre (YP_006472, 343 aa). PhiCbK gp143 is comprised of little more than the recombinase catalytic core which begins with R52, the apparent equivalent of the catalytic R173 of Cre [66]. The equivalent of the N-terminal clamp domain of Cre appears to be absent in phiCbK gp143, suggesting it could require an accessory protein for this function.

### Phage structural proteins

The structural proteome of phage phiCbK was analyzed by band excision from a SDS-PAGE gel followed by trypsin digestion and LC-MS/MS. As shown in Table 2, analysis of phiCbK directly identified eight proteins associated with the virion. The structural proteins detected in phiCbK are conserved in all six phiCbK-like phages (Table 2), indicating that despite differences in head length and genome composition, these phages are structurally very similar and likely share mechanisms of assembly and host adsorption.

**Table 2:** Virion-associated proteins of phage phiCbK

**Putative function**	**Phage protein**					
	**phiCbK**^**a**^	**Magneto**	**Swift**	**Karma**	**Rogue**	**Colossus**
Portal protein	gp42	gp40	gp40	gp43	gp46	gp38
Major capsid protein	gp68	gp66	gp66	gp69	gp70	gp81
Minor capsid protein	gp69	gp67	gp67	gp70	gp71	gp82
Major tail tube protein	gp92	gp89	gp88	gp93	gp91	gp115
Tape measure	gp95	gp92	gp91	gp96	gp94	gp118
Tail protein	gp97	gp94	gp93	gp98	gp96	gp120
Tail protein	gp99	gp96	gp95	gp100	gp98	gp122, gp123^b^
Tail protein	gp101	gp98	gp97	gp102	gp100	gp125

^a^PhiCbK proteins were detected by proteomic analysis of purified virions;

the homologous proteins in the other phiCbK-like phages are listed.

^b^PhiCbK gp99 is duplicated in phage Colossus as gp122 and gp123.

**Figure 8:**
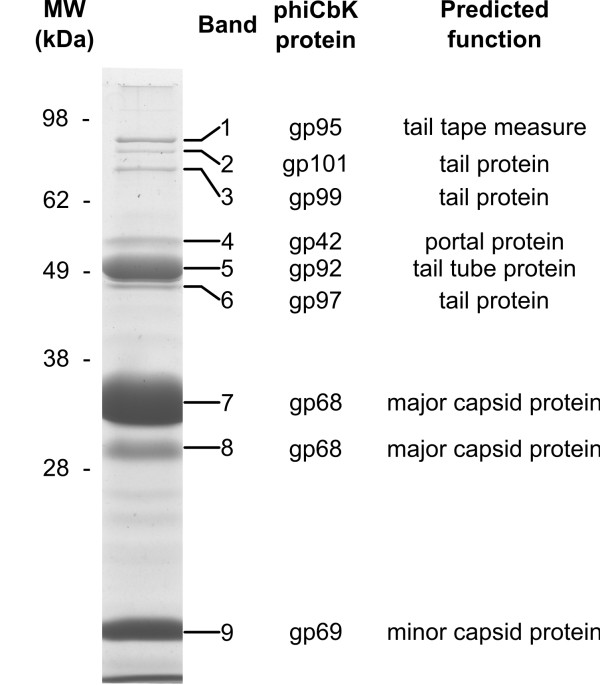
**Coomassie-stained SDS-PAGE gel of purified phiCbK virions.** The entire gel lane shown was segmented and subjected to proteomic analysis; band identities and predicted functions are annotated on the right side of the figure. While the entire gel lane was analyzed, only bands that returned conclusive peptide matches are annotated.

Considerable study has been devoted to the structural proteins of phiCbK. Previous work by Leonard *et al.*[14] has indicated the phiCbK phage head is largely composed of two proteins: a major capsid protein of 36 kDa, and a minor capsid protein of 13.5 kDa, assembled in a 1:4 ratio. A third, minor protein of 33 kDa was also reported, with a stoichiometry suggesting a possible role as a head vertex protein. As shown in Figure 8, the major capsid protein (band 7), minor capsid protein (band 9) and putative vertex protein (band 8) exhibited molecular weights similar to those reported previously, and also correspond well to their calculated molecular weights. Interestingly, the bands corresponding to the major capsid protein (band 7) and the putative vertex protein (band 8) were both identified as gp68, suggesting that the band 8 protein is a processed form of the major capsid protein. In both bands 7 and 8, peptide hits were identified that spanned from A50 to R324 of the 339 aa gp68 protein, leaving the possibility open for a cleavage event to occur somewhere in the N-terminal 49 or C-terminal 15 amino acids of this protein. The Colossus gp81 major capsid protein is very similar (71% identity) to phiCbK gp68, except that it is truncated by 29 residues at its N terminus with respect to phiCbK gp68. In Figure 8, the gp92 tail tube protein (band 5), with a predicted MW of 64.8 kDa, exhibits an apparent molecular weight of ~52 kDa which is slightly lower than the previously reported 58 kDa [14,67]. The tail tape measure protein, phiCbK gp95, was identified with the virions and appears as band 1 in Figure 8. The lengths of phiCbK gp95 (1,964 aa) and its Colossus homolog, gp118 (2,155 aa) predict tail lengths of 295 nm for phiCbK and 323 nm for Colossus assuming the alpha-helical structure found in other phage tail tape measure proteins [68]. This is good agreement with the measured lengths of the tails of these phages (Table 1). In many dsDNA phages with long tails (i.e., *Myoviridae* and *Siphoviridae*), the gene encoding the tape measure protein is preceded by a gene that encodes two tape measure chaperone proteins, one of which is an extended form of the other as specified by a programmed −1 translational frameshift [69]. In all six phages, a pre-tape measure chaperone protein (phiCbK gp93) and its extended version (phiCbK gp94) could be identified, with the frameshift site located in the slippery sequence 5’ - AAAAAAC - 3’. In Colossus, this slippery site is 5’ - GGGAAAC - 3’.

Band 4 in Figure 8 was identified as phiCbK gp42, a protein that contains a DUF4055 domain (IPR025129) but otherwise has no homologs detectable by BLASTp with experimentally confirmed functional annotations in the NCBI database. HHpred searches detected a strong relationship to PDB family 2jes_A (99.9% probability in phiCbK), which is based on the phage SPP1 portal protein. This annotation as the portal protein is supported by the positions of several gp42 homologs (e.g., XP15 (YP_239276, 27.0% identity), BcepGomr (YP_001210225, 24.5% identity) and SE2 (YP_005098128, 22.9% identity)), which are all located immediately downstream of their predicted *terL*, suggesting their function as portal proteins. The 12-member portal ring formed by the portal protein is the nucleus of phage capsid assembly and is also the docking site of the DNA packaging complex [70]. Because of their required intimate interactions with the TerL protein, portal protein genes are often genetically coupled to the large terminase gene. A recent positional analysis of phage genomes found that the location of the large terminase gene immediately upstream of the portal-encoding gene to be common [71]. In the phiCbK-like phages, this arrangement appears to be disrupted as the portal gene is located 18–20 kb downstream from *terL*.

Phage phiCbK preferentially adsorbs to the swarmer cell type [13], as stalked cells lack the both the flagellar apparatus and pili [72] required for adsorption. Phage phiCbK is known to adsorb to its host cell via a two-step process [18]. First, a filament extending from the apical vertex of the phage head associates with the rotating bacterial flagellum; this attachment is not strictly required for phage adsorption, but flagellar defects reduce adsorption efficiency by ~3-fold. Flagellar rotation brings the phage into close proximity to the cell pole, where the phage tail tip adsorbs to the cell at the site of the pilus portal and completes the infection process. Because of their similar genomic and proteomic compositions, all six phiCbK-like phages presented here are likely to use the same host adsorption mechanism. The phiCbK head filament measures ~200 nm in length and is highly flexible, but nothing is otherwise known about its structural conformation or mechanism of association with the flagellum (E. Wright, personal communication). In addition to the head filament, phage phiCbK has been observed to possess a single ~50 nm tail fiber that extends from the distal end of the phage tail [15] and is presumably involved in adsorption of the tail tip to the cell surface. The identity of the head filament and tail fiber proteins in phiCbK is of significant interest as this mechanism of phage attachment appears to be unique in phage biology.

Aside from the major tail tube subunit and the tape measure protein, three putative tail proteins were found to be associated with the phiCbK virion: gp101 (1,412 aa), gp99 (1,158 aa) and gp97 (541 aa) seen in Figure 8 as bands 2, 3 and 6, respectively. The genes encoding these proteins are in close proximity to other tail structural protein genes, the tail tube gene *92* and tail tape measure gene *95*. Gp101 exhibits weak similarity to a putative tail fiber found in *Burkholderia* phage BcepNazgul (NP_918975, E = 4.8 x 10^-11^, 7.4% identity). While gp101 does not possess the conserved fibritin domain of the BcepNazgul protein, it does contain a pair of apparent ligand-binding domains (IPR000421, IPR008979), one of which is C-terminal, which may be involved in receptor binding. The band 3 protein gp99 contains an immunoglobulin-like (Ig) domain (IPR007110) and a phage tail-like domain (PF13550), and is weakly related (E = 2 x 10^-22^, 15.9% identity) to the *Rhodobacter capsulatus* gene transfer agent orf 15 (ABK27263), which is believed to be responsible for host specificity [73]. The N-proximal position of the gp99 Ig-like domain is not likely to play a role in receptor binding, as phage tail fibers typically have their receptor-binding domains in their C-termini. The phage tail-like domain is located in the central portion of gp99, although the function of this domain is unknown. The coliphage lambda tail fiber protein J also contains this Pfam tail fiber domain, but this region of the protein is outside of the C-terminal receptor-recognition domain [74] and thus may contribute to tail fiber structure rather than binding specificity. Finally, phiCbK gp97 contains three phage-associated conserved domains (IPR011928, IPR018964, IPR019228) of unknown function. A brief survey of other well-characterized phage tail fibers shows that such fibers yield approximately one nm of length per 12–20 amino acid residues (data not shown). If phiCbK gp101 formed tail fibers of this nature, the resulting fiber length should be on the order of 70–120 nm, while gp99 would form fibers of approximately 58–95 nm, and gp97 would form fibers of 27–48 nm. All of these lengths are in reasonable agreement with the size of the previously observed ~50 nm tail fiber. The identity of this fiber cannot be conclusively assigned at this time; the size and conserved domains of gp101 and gp99 make them more probable candidates for this role.

Phage phiCbK and its relatives also encode a very large conserved protein which, while not detected as associated with the virion in this analysis, remains the most probable candidate for the head filament protein. In phiCbK, gp76 is 2,799 amino acids in length and contains a concanavalin A-like lectin domain (IPR008985, IPR013320) in its central region. Gene *76* is located in the phage structural module, and is located closer to genes involved in head morphogenesis than to those associated with the phage tail (Figure 2). While this protein is conserved within all six of the phiCbK-like phages, it does not have any similarity to other known proteins detectable by BLASTp. The protein is also highly enriched in glycine (21% of amino acid residues, with multiple poly-Gly runs), alanine (12%), and proline (7%), with glycine repeat domains (PFAM12810) in the N-terminal and central domains of the protein. The high glycine content of gp76 would be expected to result in a highly flexible structure, and analysis by PsiPred [75] suggests the protein predominantly assumes random coil or beta strand conformation. Thus, gp76 fulfills the major criteria expected of the phiCbK head filament in terms of its size, flexibility, domain architecture and genomic location. Analysis of phiCbK *76* at the DNA level suggests that this gene has undergone expansion by tandem duplications in its N-terminal half. The high molecular weight bands above band 1 in Figure 8 did not return any peptide matches following MS analysis, and digestion of entire freeze-thawed virions followed by LC-MS also did not yield any peptide matches additional to those already described (data not shown). The head filament protein may not have been directly detected in the proteomic analysis due to the fact that it is relatively fragile and could have been lost during the purification procedure (E. Wright, personal communication).

### Phage lysis proteins

**Figure 9:**
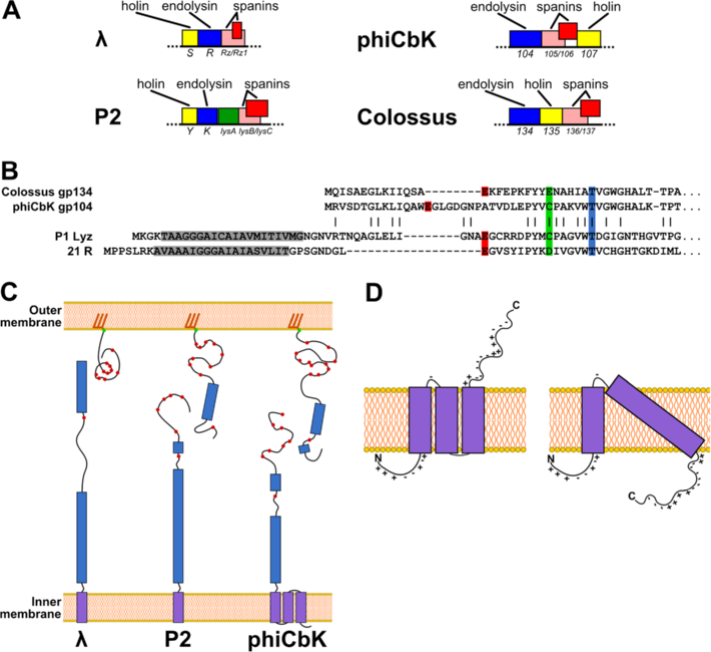
**Schematic representations of the lysis genes and proteins of *C. crescentus *phages.** Lysis components of phages phiCbK and Colossus are compared to those of other phages. **A**: Overall organization of the lysis cassettes of phages lambda, P2, phiCbK and Colossus; genes are represented by colored boxes, with gene functions labeled above each module and gene names below. Color indicates conserved function, not sequence similarity. **B**: Protein sequence alignment of the N-termini of the phages P1, 21, phiCbK and Colossus endolysins; the positions of equivalent E-D/C-T catalytic residues are highlighted in red, green and blue, transmembrane SAR domains are highlighted in grey. Identical residues in phiCbK gp104 and P1 Lyz are indicated by vertical bars, illustrating their relationship. **C**: The mature spanin proteins of phages lambda, P2 and phiCbK. Transmembrane domains are purple, predicted alpha-helical domains are blue, unstructured domains are black curves, and the positions of proline residues are represented by red dots. The spanin IM components are anchored to the inner membrane by a transmembrane helix, OM components are tethered to the outer membrane by a lipoylated (red bars) Cys residue (green dot). The P2 complex contains alpha-helical IM and OM components with proline-rich domains, and the phiCbK spanin complex more closely resembles that of P2. **D**: Possible membrane topologies of the phiCbK-like holin proteins. Left, three TMDs with N-in, C-out topology; right, two TMDs — including a 36-residue TMD2 — with N-in, C-in topology. Charge distributions of the protein and TMD length strongly favor the three TMD model.

In many phage types, the lysis genes are clustered in a "lysis cassette". For phages of Gram-negative hosts, the typical cassette consists of genes for the holin, the endolysin and the spanin subunits, responsible for permeabilization or destruction of the cytoplasmic membrane, the peptidoglycan, and the outer membrane, respectively. Of these three classes of genes, the endolysin and the spanins can be identified with the most confidence, because endolysins have a variety of easily identified catalytic motifs, and spanins have unique primary and secondary structure features. Using these considerations, the lysis cassettes were identified in all six phiCbK-like phages. The phiCbK cassette consists of genes *104* through *107* (Figure 9A), encoding the predicted endolysin, holin and spanin proteins, respectively; this order is conserved in phages Karma, Magneto, Swift and Rogue. In Colossus, the predicted holin gene has been transposed to a position following the spanin genes, resulting in the gene order endolysin-spanin-holin, encoded by genes *134* to *137*, respectively (Figure 9A). No such inversion of gene order has been detected before in phages of the same type, although among phages of the T7 phage-type the endolysin gene is in some cases transposed to an early gene transcriptional unit.

#### The endolysin proteins

All six phiCbK-like genomes have two proteins with catalytic motifs that are common in endolysins: the tail tape measure protein (gp95 in phiCbK), with a CHAP endopeptidase domain (IPR007921); and phiCbK gp104, which has a glycoside hydrolase domain (IPR002196). The presence of a CHAP domain in the tape measure protein is not unusual and is consistent with proposed mechanisms in which this protein is involved in penetrating the peptidoglycan after being ejected from the phage tail [76]. PhiCbK gp104 (gp134 in Colossus) is clearly the endolysin, since it has a strongly conserved glycoside hydrolase domain and has extensive homology to many phage proteins annotated as endolysins. However, only two coliphage homologs, P1 Lyz and R^21^ (both with an E value ~ 10^-7^) have been subjected to biochemical and structural analysis. Both are SAR endolysins carrying export signals at their N-termini [77,78], a feature not shared by the phiCbK-like endolysins. Both R^21^ and P1 Lyz belong to the canonical T4 lysozyme family, enzymes that have a characteristic N-terminal Glu-8X-Cys/Asp-5X-Thr catalytic triad. A comparison of the N-terminal domains of these endolysins (Figure 9B) suggests that the phiCbK-like proteins represent novel variants of the canonical lysozyme catalytic triad. While all have retained the Cys/Asp and Thr residues with the standard spacing, in phiCbK and its close relatives Magneto, Karma, Swift and Rogue, four nearby acidic residues are available to play the role of the catalytic Glu common to all T4-related enzymes. The simplest notion that E15 is the catalytic Glu, with the spacing between the catalytic Glu and Cys residues increased to 16 residues by the insertion of an intervening Gly-Pro rich loop. In Colossus gp134, the normal spacing would be retained but the catalytic Asp/Cys would be replaced by another Glu residue. In P1 Lyz, the presence of the Cys residue in the catalytic triad is critical to the regulation of the enzyme because it is occupied in a disulfide bond until the enzyme is activated [79]. However, phiCbK gp104 lacks a SAR domain and is thus a soluble endolysin, dependent on the holin for release to the periplasm. It does have two other Cys residues, Cys124 and Cys 213, but neither is conserved in Colossus, making disulfide bond regulation unlikely. Thus phiCbK gp104 may have evolved from a P1 Lyz-like SAR endolysin, consistent with our previous proposal that SAR endolysins might constitute the most primitive form of regulated phage lysis, predating the appearance of holins in phage genomes [80].

#### The spanin subunits

By far the most common spanin genes are those that encode two-component spanins [81]. Each two component spanin locus encodes an integral inner membrane protein (the IM spanin), and an outer membrane lipoprotein (the OM spanin); these are the functional equivalents of the lambda Rz and Rz1 proteins, respectively. Genetic, biochemical and ultrastructural analyses have shown that the two proteins interact by virtue of their C-terminal domains to form the spanin complex, thus spanning the entire periplasm [82]. During lysis, after the endolysin has degraded the peptidoglycan, the Rz-Rz1 complexes are thought to oligomerize laterally to form coiled-coil bundles, which somehow leads to OM disruption [83]. There are three known two-component spanin gene architectures: embedded, in which the gene encoding the OM spanin component is entirely contained within the IM component gene in the +1 reading frame (Figure 9A, lambda *Rz/Rz1*); overlapped, where the OM gene also starts within the gene encoding the IM component in the +1 frame but extends beyond it (Figure 9A, P2 *lysB/lysC*); and separated, where the coding regions do not overlap at all [81]. The phiCbK-like spanins (gp105/gp106 in phiCbK) are remarkably similar in their architecture to LysB and LysC found in the classical coliphage P2. As shown in Figure 9A, both gene pairs belong to the overlapped spanin class. Although the Colossus spanin proteins are significantly diverged from those of the other phiCbK-like phages (29.7% and 36.0% similarity to phiCbK in the inner and outer membrane spanins, respectively), and these bear no sequence similarity to the spanins of phage P2, the periplasmic domains of all of these IM spanins both have similar predicted alpha helical character with proline-rich C-terminal domains, unlike lambda Rz (Figure 9C). One striking feature of the phiCbK-like IM spanins is the presence of a large N-terminal hydrophobic domain preceding the anchoring TMD, which strongly suggests the presence at least one and possibly two additional TMDs. The N-terminal TMD of the IM subunit of lambda Rz can be substituted by heterologous TMDs and is thus thought to be purely a membrane tether [82]. The extra membrane component in the phiCbK-like IM spanins would be unnecessary for simple membrane tethering and thus may reflect an additional function in lysis, perhaps in coordinating the function of the spanin complex with the function of the holin, which is an IM protein in all phages. Similarly, the periplasmic domains of the phiCbK-like and P2 OM spanins are remarkably parallel in primary structural features, with an N-terminal domain that is very enriched in proline residues and a C-terminal segment predicted to be alpha-helical but containing a single distal proline (Figure 9C). Little genetic analysis has been done with any spanin so it is unclear which of these features are important for function. However, missense changes in the first two proline residues in LysC have been shown to cause a premature lysis phenotype in cells with compromised peptidoglycan [84]. To date, these *lysC* alleles are unique in being the only alleles outside of holin and antiholin genes that have early lysis phenotypes, perhaps another hint that P2-like spanins may interact functionally with their cognate holins.

#### The holin proteins

Unless there is sequence similarity with one of the very few holins that have been experimentally verified, it is difficult to identify holins with the same degree of confidence available for endolysins and spanins. However, most of the experimentally tested holins have multiple TMDs and are encoded by genes clustered within a lysis cassette; phiCbK gene *107* (gene *135* in Colossus) fits these criteria (Figure 9A) and is thus predicted to be the holin gene. Experimentally confirmed holins have been identified in three different membrane topologies: class I (3 TMDs with N-out, C-in); class II (2 TMDs with N and C in); and class III (one TMD with N-in, C-out) [85]. The first TMD of phiCbK gp107 is unambiguous, between Leu37 and Phe60, and the charge distribution flanking this TMD would put Asp66 at the periplasmic interface, so TMD1 is N-in, C-out. Beyond this, however, phiCbK-like holin topology becomes ambiguous. Commonly used algorithms like TMHMM and TMPred predict these holins to have only two TMDs, the second of which would be over 30 residues in length (Figure 9D). An alternative perspective is that this region encodes three TMDs, taking advantage of the fact that Lys residues, like Lys100, can “snorkel” and thus be mapped up to three residues inside the bilayer. This would give the phiCbK-like holins a novel topology, with three TMDs and an N-in, C-out orientation (Figure 9D). In support of this notion, the C-terminal domain beyond the ambiguous hydrophobic region is strongly acidic, unlike class I and class II holins, which both feature positively charged C-terminal cytoplasmic domains and consistent with the positive-inside, negative-outside theorem that dominates prokaryotic TMD topology [86]. It should be noted, however, that absent experimental confirmation, the identity of *107* as the holin is speculative. Especially in view of the extra TMDs present in the IM spanin subunit of phiCbK, it is worth noting that genes implicated in antiholin function have been identified in other phages, with gene products having a different variety of topologies.

### PhiCbK-like GcrA homolog

*C. crescentus* exhibits a peculiar dimorphic lifestyle, in which the stalked cell form divides asymmetrically to produce a flagellated “swarmer” cell. The swarmer cells are not capable of DNA replication nor division until they undergo the transition to the stalked morphotype (recently reviewed in [4]). Transformation of cell shape, chromosomal replication and cell division are tightly regulated in *C. crescentus* by a cascade of master regulatory proteins including DnaA [87], CtrA [3,88] and GcrA [89], and by the methylation state of the chromosome as mediated by CcrM [90]. Under normal conditions, GcrA is present in stalked cells undergoing DNA replication and division, and is not detectable in swarmer cells where CtrA is dominant [89]. CtrA binds to the GcrA transcriptional start site, negatively regulating its expression during the swarmer phase [89]. When expressed during early stages of DNA replication and cell division, GcrA up-regulates transcription of 89 *C. crescentus* genes, including several components of the cell’s DNA replication machinery such as a DNA gyrase, topoisomerase IV and a DNA pol III epsilon subunit [89].

The tropism of phage phiCbK for the swarmer cell type means that phiCbK and its relatives predominantly infect cells in which DNA replication is halted and GcrA is downregulated. It is significant, therefore, that all of the phiCbK-like phages with the exception of Colossus encode a protein with homology to the *C. crescentus* GcrA cell cycle regulator; in phage phiCbK this protein is gp222. The GcrA homologs encoded by phages phiCbK, Magneto, Swift and Karma are identical and the Rogue-encoded homolog is slightly diverged, a reflection of the overall relationship of these phages. Forty-one amino acid residues are conserved across all phage-encoded proteins and the *C. crescentus* CB15 GcrA homolog, and an additional 18 residues are similar. Furthermore, all of the phage-encoded homologs are detectable as members of the GcrA protein family (pfam07750, IPR011681). If the phage-encoded GcrA homolog possesses the same functionality as its *C. crescentus* counterpart, the expression of phiCbK gp222 in the infected swarmer cell may function to up-regulate expression of the *C. crescentus* DNA replication apparatus, components of which may be required for the successful replication of the phiCbK chromosome. GcrA is rapidly degraded in swarmer cells [87], therefore it also might be expected that the phage-encoded GcrA homologs are able to resist this degradation.

### Possible translational modulators in phage Colossus

While phage Colossus lacks the GcrA-like proteins found in the other phiCbK-like phages, it possesses a number of additional genes, not found in the other phages, that may serve to modulate the host cell to the phage’s advantage. For example, Colossus gp247 appears to be a truncated form of ribosomal elongation factor Ts (EF-Ts), with a predicted EF-Ts domain (IPR014039). EF-Ts catalyzes the cycling of GDP to GTP in elongation factor Tu (EF-Tu) by binding to the EF-Tu·GDP complex and accelerating both the release of GDP and the association of GTP. Expression of a phage-encoded EF-Ts-like protein during infection could theoretically benefit the phage by increasing the available charged EF-Tu pool and accelerating translation. While Colossus gp247 is only 98 amino acids long, approximately one-third the length of known EF-Ts proteins, it contains the N-proximal EF-Ts subdomain N with its conserved F81 residue (F32 in gp247) that plays a major role in GDP nucleotide release from EF-Tu [91]. However, gp247 seems to lack the C-proximal dimerization domain and other residues that are required for the interaction of EF-Ts with EF-Tu in other proteins, thus it is not clear if gp247 possesses full EF-Ts-like functionality.

Colossus also contains a possible ribosomal RNA methyltransferase protein, gp358. This protein possesses a RsmD-like rRNA methyltransferase domain (IPR004398) and HHpred searches detect similarity to a number of methyltransferase families with roughly equal probability, including type I restriction-like DNA methyltransferases (e.g., 2okc_A, 2ar0_A, both 99.8% probability) and rRNA methyltransferases (e.g., 1uwv_A, 99.7% probability, 3g89_A, 99.6% probability). Methylated rRNA bases tend to be clustered around active sites of the mature ribosome [92], although their function is not entirely clear as absence of methylation at these sites typically yields a mild phenotype [93,94]. Modification of the bacterial ribosome by a phage-encoded rRNA methyltransferase may provide a fitness advantage to the phage, although the function of gp358 remains to be determined.

## Conclusions

The phiCbK-like phages described here constitute a novel phage type and are distinguished by large genomes of 200 – 300 kb and prolate siphophage morphology. They all possess a T7-like DNA polymerase, suggesting that they employ a T7-like DNA replication strategy; this is the first case to our knowledge of a T7-like DNA polymerase found outside of phages of the T7 superfamily. The structural proteome of phiCbK identified the major phage structural components and several minor tail proteins of currently unknown function, although at least one of these is likely to be the tail fiber protein. The head filament protein was not conclusively established in the phiCbK virion but a strong candidate protein for this role, gp76 in phiCbK, was identified by bioinformatic analysis. These phages were found to contain several proteins that may allow the phage to more efficiently utilize cellular resources, including homologs of the GcrA cell cycle regulator and proteins that may interact with the cellular translational apparatus. The phages were sequenced by 454 pyrosequencing to coverages of 38 to 291-fold depth. These coverages were sufficient to identify coverage breakpoints that represented the boundaries of non-permuted terminal repeats in these genomes. The terminal ligation method developed here confirmed these boundaries in phage phiCbK. These methods will allow for greater throughput in the identification of phage genomic termini in the future.

## Competing interests

The authors declare that they have no competing interests.

## Authors’ contributions

JJG, JDB and RY conceived the study. JDB and JJG sequenced and assembled the phage genomes, and JJG, JDB and RFY finalized genome annotations. JJG conducted comparative genomic analyses, generated maps and figures, and conceived of and analyzed terminal labeling experiments. JJG and RY wrote the initial draft of the manuscript. WKR and DHR conducted proteomic analysis and analyzed the data. DAE prepared phage for the proteomic analysis and conducted SDS-PAGE experiments. LL conducted the terminal labeling experiments and helped close and finalize the phage genomes. MM and SM conducted experiments to close and finalize the phage genomes, and assisted with annotation. DH, AK, JK, RM and AMT conducted initial genomic annotations and analyses. All authors have read and approved the final manuscript.

## Supplementary Material

Additional file 1 **Tables S1–S6.** Complete genome annotation tables with evidence. Click here for file
