# Supplementary material for: The Caulobacter crescentus phage phiCbK: genomics of a canonical phage
Source: BMC Genomics. 2012 Oct 10;13:542. doi: 10.1186/1471-2164-13-542 (PMC3556154; doi:10.1186/1471-2164-13-542)
Supplement: Additional file 1 — Tables S1–S6. Complete genome annotation tables with evidence. [file 1471-2164-13-542-S1.pdf]

Table S1. Predicted proteins, gene starts and annotations of *C. crescentus* phage phiCbK. Upstream sequence is the 25 bp of DNA sequence upstream of the start codon, with the predicted Shine-Dalgarno motif capitalized. S-D is the Shine-Dalgarno motif associated with the gene start, and spacing is the interval (in bp) between the S-D and start codon. Evidence includes detected conserved domains or motifs, BlastP and HHpred hits, and experimental or other evidence as detailed in the text.

Protein name	Strand	Upstream Sequence	Start Codon	S-D	spacing	Predicted product	Evidence
**CbK_gp001**	+	tggcaaatcaAGGAGccggcg	atg	aggag	6	hypothetical conserved protein	
**CbK_gp002**	+	cgatttcaAGGgcttatacct	atg	agg	10	hypothetical conserved protein	
**CbK_gp003**	+	ccagcccttgtGAGGcctcgg	atg	gagg	6	hypothetical conserved protein	
**CbK_gp004**	+	ccgggcaAGGAGcttcccgcc	atg	aggag	9	hypothetical conserved protein	
**CbK_gp005**	+	gcgccttcGGAGaaaaccgcc	atg	ggag	9	hypothetical conserved protein	
**CbK_gp006**	+	cttcaatgacGGGGcggccgg	gtg	gggg	7	hypothetical conserved protein	
**CbK_gp007**	+	ggccttcaAGGAGtccgcccc	gtg	aggag	8	hypothetical conserved protein	
**CbK_gp008**	+	ctttcaAGGAttgctcccgtc	atg	agga	11	hypothetical conserved protein	
**CbK_gp009**	+	gccggccaAGGAtcgcagacc	atg	agga	9	hypothetical conserved protein	
**CbK_gp010**	+	tcctcgaaAGGAtcgccgacc	atg	agga	9	hypothetical conserved protein	
**CbK_gp011**	+	gacgacggGGAGGcgtgagcc	gtg	ggagg	8	hypothetical conserved protein	
**CbK_gp012**	+	gccgcccaAGGAttggaccgc	atg	agga	9	hypothetical conserved protein	
**CbK_gp013**	+	ccctcgcaAGGAtcgcacccc	atg	agga	9	hypothetical conserved protein	
**CbK_gp014**	+	ttgggcGGGGgtcgcctccta	atg	gggg	10	hypothetical novel protein	
**CbK_gp015**	+	catagaagAGGActaaaccca	atg	agga	9	hypothetical conserved protein	
**CbK_gp016**	+	caaccgaAGGccgcctaagcc	atg	agg	11	hypothetical conserved protein	
**CbK_gp017**	+	cccgcaAGGAGGcttaagacc	atg	aggagg	9	hypothetical conserved protein	
**CbK_gp018**	+	ccttccttcGGAGcttccaaa	atg	ggag	8	hypothetical conserved protein	
**CbK_gp019**	+	cccctctctGGAtcgctcccc	atg	gga	9	hypothetical conserved protein	
**CbK_gp020**	+	ggtcgcaAGGAGacacccgac	atg	aggag	9	hypothetical conserved protein	
**CbK_gp021**	+	cacgcaatcgAGGAGcccgtc	atg	aggag	6	hypothetical conserved protein	
**CbK_gp022**	+	tccgAGGAcggcaagcacgtc	atg	agga	13	hypothetical conserved protein	
**CbK_gp023**	+	cgcccttGGAGttcccggacg	atg	ggag	10	hypothetical conserved protein	
**CbK_gp024**	+	caacaaGAGGTaccccgcctg	atg	gaggt	10	putative lipoprotein	LipoP
**CbK_gp025**	+	acgcttgtttGGAaaatactg	atg	gga	8	hypothetical conserved protein	
**CbK_gp026**	+	acttcaacccgtAGGcgtaac	atg	agg	6	hypothetical conserved protein	
**CbK_gp027**	+	caccagcaacGAGttccattg	atg	gag	8	hypothetical conserved protein	
**CbK_gp028**	+	catccccatcggaGAGGcctg	atg	gagg	4	hypothetical conserved protein	
**CbK_gp029**	+	ctacgagcgcgtGGAGGcccc	gtg	ggagg	4	hypothetical conserved protein	
**CbK_gp030**	+	ggtcccgctGGAGccgctgcc	gtg	ggag	8	hypothetical conserved protein	
**CbK_gp031**	+	tcgccggcaAGGAGGcgatcc	atg	aggagg	6	hypothetical conserved protein	
**CbK_gp032**	+	gccccgcaAGGAGcgtgtctt	ttg	aggag	8	hypothetical conserved protein	
**CbK_gp033**	+	agacgccgcctgaAGGAcatc	atg	agga	4	hypothetical conserved protein	
**CbK_gp034**	+	tcgccccgtaacgAGGAcccc	atg	agga	4	hypothetical conserved protein	
**CbK_gp035**	+	ccctgatcaccGAGGcctgac	atg	gagg	6	hypothetical conserved protein	
**CbK_gp036**	+	gggcatgaagaAGGcgaagtc	atg	agg	7	hypothetical conserved protein	
**CbK_gp037**	+	gcgcGGAcaactggcggcggc	atg	gga	14	hypothetical conserved protein	
**CbK_gp038**	+	ggcatcaAGGccgccttcagc	ctg	agg	11	hypothetical conserved protein	
**CbK_gp039**	+	cgaaactgtGAGGcccattcg	atg	gagg	8	hypothetical conserved protein	
**CbK_gp040**	-	gctacctcaAGGAGtagcacc	gtg	aggag	7	hypothetical conserved protein	
**CbK_gp041**	-	gacacttaaGGGGtgtcgatc	atg	gggg	8	putative transglutaminase-like cysteine peptidase	IPR010319
**CbK_gp042**	+	gcgacctcgcAGGccatctat	ctg	agg	8	portal protein	HHpred vs pdb70_18Aug12, hit 2jes_A, prob 99.9% E=4.9E-23; positional evidence, see text
**CbK_gp043**	+	cccgaacccgAGGAccgcccg	atg	agga	7	hypothetical conserved protein	
**CbK_gp044**	+	aaccctagcttgcaGAGtaac	atg	gag	4	hypothetical conserved protein	
**CbK_gp045**	+	ccgccccaagaaagcgacctg	atg	None	0	hypothetical conserved protein	
**CbK_gp046**	+	cctccagccttGAGGcgcccc	gtg	gagg	6	hypothetical conserved protein	
**CbK_gp047**	+	gttaaccacGAGGgcttttcc	atg	gagg	8	hypothetical conserved protein	
**CbK_gp048**	+	gctcctttcccaGAGGgctcc	atg	gagg	5	hypothetical conserved protein	
**CbK_gp049**	+	cgaccgcgtgcGGGGcacctg	atg	gggg	6	hypothetical conserved protein	
**CbK_gp050**	+	tccaagcttAGGAGcccgtcc	atg	aggag	7	hypothetical conserved protein	
**CbK_gp051**	+	gatcaccgcctcggtcgagac	gtg	None	0	hypothetical conserved protein	
**CbK_gp052**	+	gaccatctacGAGagcacgcg	atg	gag	6	hypothetical conserved protein	
**CbK_gp053**	+	ggtgaccttcagGGAagagcc	gtg	gga	6	hypothetical conserved protein	
**CbK_gp054**	+	tccggtcgAGGAGGgtgacat	ttg	aggagg	7	hypothetical conserved protein	
**CbK_gp055**	+	gcgcgaagccgccaagcgtcc	atg	None	0	hypothetical conserved protein	
**CbK_gp056**	+	ttaaccatgacgggctccctg	atg	None	0	hypothetical conserved protein	
**CbK_gp057**	+	gatgggcgAGGAGcgctacca	gtg	aggag	8	putative HD-domain/PDEase-like protein	SSF109604
**CbK_gp058**	+	aacgatacGAGaacatccgct	atg	gag	10	hypothetical conserved protein	
**CbK_gp059**	+	gctatctGGAccgctgagccg	atg	gga	11	hypothetical conserved protein	
**CbK_gp060**	+	cctgccgaagaAGGccgcctg	atg	agg	7	putative DHH phosphoesterase protein	SSF64182
**CbK_gp061**	+	caagcgccgggcGGGGacggc	gtg	gggg	5	hypothetical conserved protein	
**CbK_gp062**	+	tctgccaacAGGAGatcgccg	gtg	aggag	7	hypothetical conserved protein	
**CbK_gp063**	+	ttccagcacatctAGGAcccc	atg	agga	4	hypothetical conserved protein	
**CbK_gp064**	+	gattacGAGGcctatcaccac	gtg	gagg	11	hypothetical conserved protein	
**CbK_gp065**	+	ttcgctgcGAGGccatttctg	atg	gagg	9	hypothetical conserved protein	
**CbK_gp066**	+	aagccggcgcgcaacttccgg	atg	None	0	putative HNH endonuclease	IPR002711
**CbK_gp067**	+	ccgtgggtaGAGagaaactgt	atg	gag	7	hypothetical conserved protein	
**CbK_gp068**	+	cgtgacaaGAGaattccctcg	gtg	gag	10	major capsid protein	experimental evidence, see text
**CbK_gp069**	+	atctaagcAGGAaatccccag	atg	agga	9	minor capsid protein	experimental evidence, see text
**CbK_gp070**	+	cccccgaaacGGAaaatcccc	atg	gga	8	hypothetical conserved protein	

**CbK_gp071**	+	aagcccacggcggcctaagcc	atg	None	0	hypothetical conserved protein	
**CbK_gp072**	+	atcacccAGGTgaactaagcc	gtg	aggt	10	hypothetical conserved protein	
**CbK_gp073**	+	caactccgctacgcctaagtc	atg	None	0	hypothetical conserved protein	
**CbK_gp074**	+	ttcacgaactaagGGAcggcc	atg	gga	5	hypothetical conserved protein	
**CbK_gp075**	+	accccccAGGAGccctgaacc	atg	aggag	9	hypothetical conserved protein	
**CbK_gp076**	+	ccgcctcaAGGgtccttcgcg	ttg	agg	10	putative lectin-like domain protein	IPR013320, IPR008985
**CbK_gp077**	+	cagacctGGAGGcctaagacc	atg	ggagg	9	hypothetical conserved protein	
**CbK_gp078**	+	ttccgctcgcaGGGGcgccgc	gtg	gggg	6	hypothetical conserved protein	
**CbK_gp079**	+	tctGGAGtttcgagcgccatc	atg	ggag	14	hypothetical conserved protein	
**CbK_gp080**	+	acgcaccgccAGGgcttttcc	atg	agg	8	hypothetical conserved protein	
**CbK_gp081**	+	ctgcggtcgcgcGAGGcttcc	atg	gagg	5	hypothetical conserved protein	
**CbK_gp082**	-	tctcaaccttAGGAGcagctt	atg	aggag	6	hypothetical conserved protein	
**CbK_gp083**	+	ggcggccgttcGAGagctatc	atg	gag	5	hypothetical conserved protein	
**CbK_gp084**	+	agtcGGGGaccgagagccgtc	atg	gggg	13	hypothetical conserved protein	
**CbK_gp085**	+	tcctgggtGGAGttttcccta	atg	ggag	9	hypothetical conserved protein	
**CbK_gp086**	+	cgccatcccagagGGAGcgac	gtg	ggag	4	putative PhoH-like protein	IPR003714, SSF52540, G3DSA:3.40.50.300
**CbK_gp087**	+	gtgacaaagacgtagcgcacc	atg	None	0	hypothetical conserved protein	
**CbK_gp088**	+	caagaagatcattcgccacta	atg	None	0	hypothetical conserved protein	
**CbK_gp089**	+	cagcacgcGGAagaagacccc	gtg	gga	10	hypothetical conserved protein	
**CbK_gp090**	+	ctcgattaaaGAGGTgttcgg	atg	gaggt	6	hypothetical conserved protein	
**CbK_gp091**	+	gttttggctgggaGAGGactg	atg	gagg	4	hypothetical conserved protein	
**CbK_gp092**	+	ccgtGGAttggtgacctgccc	atg	gga	14	major tail tube protein	experimental evidence, see text
**CbK_gp093**	+	cccgatacGGAatccccgaac	atg	gga	10	putative pre-tape measure chaperone protein	HHpred vs pdb70_18Aug12, hit 2ob9_A, prob 89.3% E=0.82; positional evidence, see text
**CbK_gp094**	+	cccgatacGGAatccccgaac	atg	gga	10	putative pre-tape measure chaperone protein, frameshifted version	positional evidence, see text; slippery sequence AAAAAAC
**CbK_gp095**	+	gcGGGGccgccgataggatca	atg	gggg	15	tail tape measure protein	IPR007921, IPR013491, IPR013423; experimental evidence, see text
**CbK_gp096**	+	tcctttcatggttaatgcccg	atg	None	0	hypothetical conserved protein	IPR011740
**CbK_gp097**	+	ctggcaaGAGGccctgacgcc	gtg	gagg	10	virion-associated protein, putative tail protein	IPR019228, IPR018964, IPR011928; experimental evidence, see text
**CbK_gp098**	+	gcccaacgctccGGAGcagga	atg	ggag	5	putative NlpC/P60 family cell wall peptidase	IPR000064, SSF54001, G3DSA:3.90.1720.10
**CbK_gp099**	+	ccccggcgttgAGGActaatc	atg	agga	6	virion-associated protein, putative tail protein	IPR007110; experimental evidence, see text
**CbK_gp100**	+	tgtcataaaagtcgtggaacg	atg	None	0	hypothetical conserved protein	
**CbK_gp101**	+	cctcaAGGgcgtcatcctgca	atg	agg	13	virion-associated protein, putative tail protein	IPR000421, IPR008979, G3DSA:2.60.120.260; experimental evidence, see text
**CbK_gp102**	+	accctcaagGGAatccgatag	atg	gga	9	hypothetical conserved protein	
**CbK_gp103**	+	tcttcatgctcGGAGcctgac	atg	ggag	6	hypothetical conserved protein	
**CbK_gp104**	+	tcagcacttccGGAattccac	atg	gga	7	putative endolysin	IPR002196, SSF53955, G3DSA:1.10.530.40
**CbK_gp105**	+	gggcttcaAGGgctgctgatc	atg	agg	10	putative inner membrane spanin component	N-terminal TMD, outer membrane spanin component embedded in +1 frame, see text
**CbK_gp106**	+	agtaccaaGAGGTtattcacg	atg	gaggt	8	putative outer membrane spanin component	lipoprotein embedded in inner membrane spanin component, see text
**CbK_gp107**	+	caagctcaaAGGgcagaagca	gtg	agg	9	putative holin protein	predicted 4 TMDs, see text
**CbK_gp108**	+	cgctatcacGGAGtagggacc	atg	ggag	8	hypothetical conserved protein	
**CbK_gp109**	-	cgacggacggAGGAGaacgaa	atg	aggag	6	putative HTH domain DNA-binding protein	IPR001387, IPR010982, G3DSA:1.10.260.40
**CbK_gp110**	-	ttaaccaGAGGcccgaaaata	atg	gagg	10	putative dUTP pyrophosphatase	IPR008181, IPR008180, SSF51283, PTHR11241, G3DSA:2.70.40.10
**CbK_gp111**	-	tccttttcctGAGaccccttc	atg	gag	8	putative ribonucleoside diphosphate reductase beta subunit	IPR012348, IPR012335, IPR000358, IPR002109, IPR009078, IPR012336, IPR011767
**CbK_gp112**	-	tcgacgcGAGaacGAGacccc	atg	gag	5	putative ribonucleoside diphosphate reductase alpha subunit	IPR006141, IPR003587, IPR000788, IPR013509, IPR008926, SSF51294, SSF51998, G3DSA:2.170.16.10, G3DSA:3.20.70.20
**CbK_gp113**	-	cggtggttGGGGtggctggcg	ttg	gggg	9	hypothetical conserved protein	
**CbK_gp114**	-	tcctcatgcagcAGGAatccc	atg	agga	5	hypothetical conserved protein	
**CbK_gp115**	-	acgacatcaccGGAGccgagc	atg	ggag	6	hypothetical conserved protein	
**CbK_gp116**	-	gacgccgaccAGGActgaccc	atg	agga	7	putative thymidylate synthase	IPR003669
**CbK_gp117**	-	atccttcgctAGGAGaacccc	atg	aggag	6	putative dNMP kinase	HHpred vs pdb70_18Aug12, hit 2grj_A, prob 100% E=7.4E-28; SSF52540, G3DSA:3.40.50.300
**CbK_gp118**	-	aagatcaAGGAcgggcaaccc	ctg	agga	10	putative RecD-like helicase	HHpred vs pdb70_18Aug12, hit 3e1s_A, prob 100% E=1.3E-45; SSF52540, G3DSA:3.40.50.300
**CbK_gp119**	-	acagaaaatAGGTatcagcaa	atg	aggt	8	hypothetical conserved protein	
**CbK_gp120**	-	aGAGaaatgtgacggtgcgcg	atg	gag	17	hypothetical conserved protein	
**CbK_gp121**	-	actcccgaacGAGatcgcccg	atg	gag	8	putative exoribonuclease, Pol III-like	IPR006055, IPR013520, IPR012337, G3DSA:3.30.420.10
**CbK_gp122**	-	cacctgggcGGAGacgcactg	atg	ggag	8	hypothetical conserved protein	
**CbK_gp123**	-	atcaaatcctcaAGGTgctcc	atg	aggt	5	putative Pol I DNA polymerase, T7-like	IPR001098, IPR012337, SSF56672, PTHR10133, G3DSA:1.10.150.20, G3DSA:1.20.1060.10, G3DSA:3.30.420.10, G3DSA:3.30.70.370; see text
**CbK_gp124**	-	acatccgttaaccatagagcc	atg	None	0	putative DNA cytosine methyltransferase	IPR001525, IPR018117, SSF53335, PTHR10629:SF11, G3DSA:3.40.50.150, G3DSA:3.90.120.10
**CbK_gp125**	-	ggcgcctgtaagcGAGGtccc	atg	gagg	4	hypothetical conserved protein	
**CbK_gp126**	-	gcgccaaaccGGAGcttctct	atg	ggag	7	putative T5 A1-like protein	blastP to T5 A1 (AAU05157), E=2.81E-89, 34.2% Dice identity; HHpred vs pdb70_18Aug12, hit 1g2h_A, prob 94.0% E=0.049
**CbK_gp127**	-	tccggatatgccAGGAccccg	ctg	agga	5	putative DNA methylase	IPR002052, SSF53335, G3DSA:3.40.50.150
**CbK_gp128**	-	aggctccgttcgcgGGActtg	ctg	gga	4	hypothetical conserved protein	
**CbK_gp129**	-	aaagatcgctatcgttgtcgt	atg	None	0	hypothetical conserved protein	
**CbK_gp130**	-	catcgcaaAGGTcatggacac	gtg	aggt	9	hypothetical conserved protein	
**CbK_gp131**	-	cgttaaccacgtgcggcgccc	atg	None	0	putative DNA helicase	IPR014001, IPR001650, IPR000330, IPR014021, SSF52540, PTHR10799, G3DSA:3.40.50.300
**CbK_gp132**	+	ccggtcacaAGGAGaactatc	atg	aggag	7	hypothetical conserved protein	
**CbK_gp133**	-	ggaaatcaAGGAagccgcgtg	atg	agga	9	hypothetical conserved protein	
**CbK_gp134**	-	caccgccgcaaAGGccccctg	atg	agg	7	hypothetical conserved protein	
**CbK_gp135**	-	acccacGAGGTctcgcgctac	ctg	gaggt	10	hypothetical conserved protein	
**CbK_gp136**	-	gccttttcaAGGAtcgcttcg	atg	agga	8	hypothetical conserved protein	
**CbK_gp137**	-	gccggtctcaAGGAGacgacc	atg	aggag	6	Putative rIIb-like protein	PD130832
**CbK_gp138**	-	gcctgcctttcGGAGtttgcc	atg	ggag	6	putative rIIa-like protein	IPR003594
**CbK_gp139**	+	ggcaaactccgaaAGGcAGGc	ttg	agg	1	hypothetical conserved protein	
**CbK_gp140**	+	gcctGAGGcatgctcgtattc	atg	gagg	13	hypothetical conserved protein	
**CbK_gp141**	+	cgtatggtacgcgAGGAtgac	atg	agga	4	hypothetical conserved protein	
**CbK_gp142**	+	caccgacgggcgcgtcctcca	ttg	None	0	hypothetical conserved protein	
**CbK_gp143**	+	acgacaacgaAGGAGGctctc	atg	aggagg	5	putative tyrosine recombinase	IPR013762, IPR002104, IPR011010
**CbK_gp144**	+	gtcctgcgatAGGAGGgcgcg	atg	aggagg	5	hypothetical conserved protein	

**CbK_gp145**	+	ccgttagtccAGGAGtacagt	ttg	aggag	6	hypothetical conserved protein	
**CbK_gp146**	+	acccccgtcccgacAGGcttc	atg	agg	4	hypothetical conserved protein	
**CbK_gp147**	+	atcgctcctGGGGccttttga	gtg	gggg	8	hypothetical conserved protein	
**CbK_gp148**	+	tcctgtacggtaagGGAaaac	atg	gga	4	hypothetical conserved protein	
**CbK_gp149**	+	acgattcGGAGtattcatgaa	atg	ggag	10	putative HTH domain protein	IPR001387, G3DSA:1.10.260.40
**CbK_gp150**	+	cctgtattcctGAGGTcccgc	atg	gaggt	5	putative tRNA amidotransferase domain protein	IPR019004, IPR003789
**CbK_gp151**	-	acttatcGAGGgaaagttgag	atg	gagg	10	putative DNA ligase	IPR012340, IPR016027, SSF56091, G3DSA:3.30.470.30
**CbK_gp152**	-	tcgatccGGAGattttcggcg	atg	ggag	10	hypothetical conserved protein	
**CbK_gp153**	-	gccatcccctctatctccgag	gtg	None	0	hypothetical conserved protein	
**CbK_gp154**	-	gtttcAGGgcaaatgtcAGGa	atg	agg	1	DUF1643 protein	IPR012441
**CbK_gp155**	-	gcctcgcccttgaAGGAggca	atg	agga	4	hypothetical conserved protein	
**CbK_gp156**	-	gaagctaagaagcgggccacg	gtg	None	0	hypothetical conserved protein	
**CbK_gp157**	-	ctGGAtcgccccggcggcctg	atg	gga	16	hypothetical conserved protein	
**CbK_gp158**	-	gcttttgAGGAGtttggacgt	atg	aggag	9	hypothetical conserved protein	
**CbK_gp159**	-	agcaggactAGGAGaaagacc	atg	aggag	7	hypothetical conserved protein	
**CbK_gp160**	-	cgacgccaAGGAcgtctgcca	atg	agga	9	hypothetical conserved protein	
**CbK_gp161**	-	ggccggctacGAGatcgcctg	atg	gag	8	putative lipoprotein	LipoP
**CbK_gp162**	-	cgcttaaaGGGGgacctcatc	atg	gggg	8	putative nicotinate phosphoribosyltransferase	IPR015977, IPR002638, PTHR11098
**CbK_gp163**	-	cagaatgaGGGGtaagcgata	gtg	gggg	9	putative NUDIX hydrolase domain protein	IPR014729, IPR000086, IPR015797, SSF52374, PS51462, PTHR22769
**CbK_gp164**	-	aatacaagaacccaaagcgta	gtg	None	0	hypothetical conserved protein	
**CbK_gp165**	-	cgcaacgcctgGGActcactg	atg	gga	7	putative peptidyl-tRNA hydrolase	IPR002833, SSF102462, PTHR12649, G3DSA:3.40.1490.10
**CbK_gp166**	-	agcgccggccgccGAGGgctg	atg	gagg	4	hypothetical conserved protein	
**CbK_gp167**	-	taacctccagcAGGAGaccgc	atg	aggag	5	hypothetical conserved protein	
**CbK_gp168**	-	acaacgccGGAGGccctttc	atg	ggagg	8	hypothetical conserved protein	
**CbK_gp169**	-	ccccgatctccGAGaagcccc	atg	gag	7	putative HD-domain/PDEase-like protein	SSF109604
**CbK_gp170**	-	gggagaattcctGAGGgtcgc	atg	gagg	5	hypothetical conserved protein	
**CbK_gp171**	-	acgcccagaAGGActaatcct	atg	agga	8	putative DNA-binding domain protein	IPR016177, SSF54060
**CbK_gp172**	-	gaggcctcGGAGaagtaagcc	atg	ggag	9	hypothetical conserved protein	
**CbK_gp173**	-	tcttgagaAGGAacaaccctg	atg	agga	9	hypothetical conserved protein	
**CbK_gp174**	-	tcaacaAGGgcaagaacgccg	atg	agg	12	hypothetical conserved protein	
**CbK_gp175**	-	caagcAGGcggcgggtctgct	ttg	agg	13	hypothetical conserved protein	
**CbK_gp176**	-	gctgttcgactGGAGaaaatc	gtg	ggag	6	hypothetical conserved protein	
**CbK_gp177**	-	ctctgcgtgaAGGgctAGGcc	atg	agg	2	hypothetical conserved protein	
**CbK_gp178**	-	acaccacagaAGGAGcccgcc	gtg	aggag	6	putative RtcB-like protein	IPR001233
**CbK_gp179**	-	taccccttcaAGGTgatcccc	atg	aggt	7	hypothetical conserved protein	
**CbK_gp180**	-	aacaaagcaAGGAGcaacccc	ttg	aggag	7	putative band 7 lipoprotein	IPR001107
**CbK_gp181**	-	actcctgaaAGGccgcgccca	atg	agg	9	hypothetical conserved protein	
**CbK_gp182**	-	gcagcgcgggctcgcggcctg	atg	None	0	hypothetical conserved protein	
**CbK_gp183**	-	acgacaacgaAGGAGacctcc	atg	aggag	6	hypothetical conserved protein	
**CbK_gp184**	-	caggatGGAGGccgccgccaa	atg	ggagg	10	hypothetical conserved protein	
**CbK_gp185**	-	tccgagccaAGGAGcgctgat	ttg	aggag	7	hypothetical conserved protein	
**CbK_gp186**	-	cgacagcgagAGGAGccgatc	atg	aggag	6	hypothetical conserved protein	
**CbK_gp187**	-	gcactctggaaaAGGAccacc	atg	agga	5	hypothetical conserved protein	
**CbK_gp188**	-	ggtcctctcgGGAGacaatcc	atg	ggag	7	hypothetical conserved protein	
**CbK_gp189**	-	tctgaaAGGctcgacctcctg	atg	agg	12	hypothetical conserved protein	
**CbK_gp190**	-	cgttccaactcgcAGGTctcc	gtg	aggt	4	hypothetical conserved protein	
**CbK_gp191**	-	cctgggcctGGAGaagcacct	gtg	ggag	8	hypothetical conserved protein	
**CbK_gp192**	-	gccctatatGAGcGAGcagca	gtg	gag	5	hypothetical conserved protein	
**CbK_gp193**	-	gacgacaacgAGGAGGccctg	atg	aggagg	5	putative ArdC-like antirestriction protein	IPR017113, IPR013610
**CbK_gp194**	-	cgcggcgataatGAGcgcctg	atg	gag	6	hypothetical conserved protein	
**CbK_gp195**	-	gggccggGGAGGgcgtgcatc	atg	ggagg	9	hypothetical conserved protein	
**CbK_gp196**	-	cgcctgccaGAGcctcaagtc	atg	gag	9	hypothetical conserved protein	
**CbK_gp197**	-	aacgacaccagggGGAGagcc	gtg	ggag	4	putative acyl carrier protein	IPR009081, IPR006163, PTHR20863
**CbK_gp198**	-	tcgacaacggcaAGGAcggct	atg	agga	5	putative HNH endonuclease	IPR002711
**CbK_gp199**	-	ctcattcgttaAGGAtcgacc	atg	agga	6	hypothetical conserved protein	
**CbK_gp200**	-	cgttcctgggctatcaaaccg	atg	None	0	hypothetical conserved protein	
**CbK_gp201**	-	tggGGAGaagctgaagtgagc	atg	ggag	14	hypothetical conserved protein	
**CbK_gp202**	-	cgccttcGAGGTgcaagcctg	atg	gaggt	9	hypothetical conserved protein	
**CbK_gp203**	-	tccggacctcGGAGaaggccc	gtg	ggag	7	hypothetical conserved protein	
**CbK_gp204**	-	gcggttcgAGGAGatcgaggc	ttg	aggag	8	hypothetical conserved protein	
**CbK_gp205**	-	gacgacaacggaAGGAaccct	atg	agga	5	hypothetical conserved protein	
**CbK_gp206**	-	cggtgacttcgccaAGGggac	atg	agg	4	hypothetical conserved protein	
**CbK_gp207**	-	gcgcgaaccgaAGGTcgactg	atg	aggt	6	hypothetical conserved protein	
**CbK_gp208**	-	gacgacaacaAGGAGGcctcc	atg	aggagg	5	hypothetical conserved protein	
**CbK_gp209**	-	ctacgtGGAGagccgggcggc	atg	ggag	11	hypothetical conserved protein	
**CbK_gp210**	-	cgcgcaatttccGGAGtgacc	atg	ggag	5	hypothetical conserved protein	SSF56784
**CbK_gp211**	-	ggcctgcgcgGGAGccctgtg	atg	ggag	7	hypothetical conserved protein	
**CbK_gp212**	-	tgcagggcctgGGAGactagg	gtg	ggag	6	hypothetical conserved protein	
**CbK_gp213**	-	gatcaagtcGAGGccctgagc	atg	gagg	8	hypothetical conserved protein	
**CbK_gp214**	-	ggctcccgtacAGGAtcacct	atg	agga	6	hypothetical conserved protein	
**CbK_gp215**	-	caccgggctcgtggcggcctg	atg	None	0	hypothetical conserved protein	
**CbK_gp216**	-	agcagaAGGAGGaagccgctc	atg	aggagg	9	putative HTH domain DNA-binding protein	IPR010982, IPR001387, G3DSA:1.10.260.40
**CbK_gp217**	-	aatcgcccAGGgcgccgcctg	atg	agg	10	hypothetical conserved protein	
**CbK_gp218**	-	acaacgacagaAGGAGGccgc	gtg	aggagg	4	hypothetical conserved protein	

**CbK_gp219**	-	gaggcgggcgaGGGGcacgtc	gtg	gggg	6	hypothetical conserved protein	
**CbK_gp220**	-	cactgtccggaAGGTcgagcc	atg	aggt	6	hypothetical conserved protein	
**CbK_gp221**	-	ggtctatcgccGGAGGgcctg	atg	ggagg	5	putative Pol I DNA polymerase, palm domain	IPR001098, IPR002298, SSF56672, G3DSA:3.30.70.370
**CbK_gp222**	-	agatcaAGGAGtagtgcaagc	gtg	aggag	10	putative GcrA-like cell cycle regulator	IPR011681; see text
**CbK_gp223**	-	gccgccaaattcGAGGggaag	atg	gagg	5	hypothetical conserved protein	
**CbK_gp224**	+	cgacaacgaaAGGAGcctgcc	atg	aggag	6	hypothetical conserved protein	
**CbK_gp225**	+	cgccgcGAGGctcgcccaatg	atg	gagg	11	hypothetical conserved protein	
**CbK_gp226**	+	caagatcGAGGaaGAGGtctg	atg	gagg	4	hypothetical conserved protein	
**CbK_gp227**	+	cattcgcgtgttGGGGctcgg	atg	gggg	5	hypothetical conserved protein	
**CbK_gp228**	+	caagctcaagaaAGGTcgcct	atg	aggt	5	hypothetical conserved protein	
**CbK_gp229**	+	cattctcgactAGGAGGaccc	atg	aggagg	4	hypothetical conserved protein	
**CbK_gp230**	+	tcgatttcctggcgggcgccg	atg	None	0	hypothetical conserved protein	
**CbK_gp231**	+	gtttctggaAGGTcaggactg	atg	aggt	8	hypothetical conserved protein	
**CbK_gp232**	+	agcgcgagccgcAGGTctgac	atg	aggt	5	hypothetical conserved protein	
**CbK_gp233**	+	cgacagcgaaAGGAGcctgcc	ttg	aggag	6	hypothetical conserved protein	
**CbK_gp234**	+	tcggatgctGGAGGTcgcggc	ttg	ggaggt	6	hypothetical conserved protein	
**CbK_gp235**	+	cgccgtggAGGAGatgcgccg	gtg	aggag	8	hypothetical conserved protein	
**CbK_gp236**	+	cgccgacttcaAGGAcaagcg	atg	agga	6	hypothetical conserved protein	
**CbK_gp237**	+	accgccGGAGGcagacccgac	gtg	ggagg	10	hypothetical conserved protein	
**CbK_gp238**	+	tcaagctGGAGaccgtctgac	atg	ggag	10	hypothetical conserved protein	
**CbK_gp239**	+	acgacaacgagAGGAGttccc	atg	aggag	5	hypothetical conserved protein	
**CbK_gp240**	+	cacggaatgGGAGattttcga	atg	ggag	8	hypothetical conserved protein	
**CbK_gp241**	+	ggcctGGAGatcatcgcctag	atg	ggag	12	hypothetical conserved protein	
**CbK_gp242**	+	aagacctgaAGGAagactgac	atg	agga	8	hypothetical conserved protein	
**CbK_gp243**	+	ggcccccgAGGAGcccaaagc	atg	aggag	8	hypothetical conserved protein	
**CbK_gp244**	+	ggtcagccAGGAcatgccgtc	gtg	agga	9	hypothetical conserved protein	
**CbK_gp245**	+	gacgacaacgAGGAGGcttcg	atg	aggagg	5	hypothetical conserved protein	
**CbK_gp246**	+	gtcaaagAGGAGGcctgagcc	ttg	aggagg	8	hypothetical conserved protein	
**CbK_gp247**	+	ggctccctacGAGGccccaca	atg	gagg	7	hypothetical conserved protein	
**CbK_gp248**	+	ccggcgAGGAGGTccgctgac	atg	aggaggt	8	hypothetical conserved protein	
**CbK_gp249**	+	tggacgacaacAGGAGGgacc	atg	aggagg	4	hypothetical conserved protein	
**CbK_gp250**	+	ctatgtgaAGGAcctgcgtcg	atg	agga	9	hypothetical conserved protein	
**CbK_gp251**	+	tcagcgtGGGGgtggctgctg	atg	gggg	9	hypothetical conserved protein	
**CbK_gp252**	+	gacccaGGGGgttcccgaggc	atg	gggg	10	hypothetical conserved protein	
**CbK_gp253**	+	cctcgacgcgAGGAaaagctg	atg	agga	7	hypothetical conserved protein	
**CbK_gp254**	+	cctgaAGGAaggcggaaccga	atg	agga	12	hypothetical conserved protein	
**CbK_gp255**	+	ttgtcttgagaAGGcccgcac	atg	agg	7	DUF1937 protein	IPR015235, SSF52309
**CbK_gp256**	+	gagcaccagGGAGattgaagc	gtg	ggag	8	hypothetical conserved protein	
**CbK_gp257**	+	ggtgtcccaGAGGTaccagcg	atg	gaggt	7	hypothetical conserved protein	
**CbK_gp258**	+	ccggcgcctatGGAGagagcc	atg	ggag	6	putative TAT signal protein	IPR006311;PRED-TAT
**CbK_gp259**	+	tctcgatatggcgaaaagcgc	atg	None	0	hypothetical conserved protein	
**CbK_gp260**	+	acgtcaacaAGGAGcccaccg	atg	aggag	7	hypothetical conserved protein	
**CbK_gp261**	+	agaagcccggcgggccgagtg	atg	None	0	hypothetical conserved protein	
**CbK_gp262**	+	gcggtcgaaatcctGGAgacc	ctg	gga	4	hypothetical conserved protein	
**CbK_gp263**	+	gccgctcgacgggtGGAacgg	atg	gga	4	hypothetical conserved protein	
**CbK_gp264**	+	cgaggaagAGGAGcgctgctg	atg	aggag	8	putative Ser/Thr protein phosphatase	IPR004843, IPR006186, SSF56300, PTHR11668, G3DSA:3.60.21.10
**CbK_gp265**	+	gttgatccgcgAGGAcgacgc	atg	agga	6	hypothetical conserved protein	
**CbK_gp266**	+	gacgacaacgAGGAGGcccac	atg	aggagg	5	hypothetical conserved protein	
**CbK_gp267**	+	catgaccaaGAGccgcttctg	atg	gag	9	hypothetical conserved protein	
**CbK_gp268**	+	gatcatcctgGGAGacgccgc	atg	ggag	7	putative TrmB-like transcriptional regulator	IPR002831
**CbK_gp269**	+	ccgtttcgAGGAGGctcgccc	atg	aggagg	7	hypothetical conserved protein	
**CbK_gp270**	+	cgcaagAGGAGcccccgagcc	gtg	aggag	10	hypothetical conserved protein	
**CbK_gp271**	+	gccacggaagAGGAcgcctag	atg	agga	7	hypothetical conserved protein	
**CbK_gp272**	+	acgagcgacgGGAGGgctgac	atg	ggagg	6	hypothetical conserved protein	
**CbK_gp273**	+	gagacaaAGGgccttaagtcc	atg	agg	11	hypothetical conserved protein	
**CbK_gp274**	+	aacggaAGGAcctgaaagacc	atg	agga	11	hypothetical conserved protein	
**CbK_gp275**	+	caacgccGGAGGccctttttc	atg	ggagg	9	hypothetical conserved protein	
**CbK_gp276**	+	atactacgccaagcAGGccgc	atg	agg	4	hypothetical conserved protein	
**CbK_gp277**	+	caacatcgaaGAGttcggcga	atg	gag	8	hypothetical conserved protein	
**CbK_gp278**	+	ccagatcaAGGAGGcccagac	ttg	aggagg	7	hypothetical conserved protein	
**CbK_gp279**	+	aatcagcaataGGGGaccagc	atg	gggg	6	hypothetical conserved protein	
**CbK_gp280**	+	ctgggtccaGGGGaccggcgc	gtg	gggg	8	hypothetical conserved protein	
**CbK_gp281**	+	cggacatagAGGAGcaccccg	atg	aggag	7	hypothetical conserved protein	
**CbK_gp282**	+	acgccgcctgAGGAGGacccg	atg	aggagg	5	hypothetical conserved protein	
**CbK_gp283**	+	gcttgagaAGGcgttcggcca	atg	agg	10	hypothetical conserved protein	
**CbK_gp284**	+	ccaagcctcGGGGctcttcca	atg	gggg	8	hypothetical conserved protein	
**CbK_gp285**	+	caccgactactAGGAGGtccc	atg	aggagg	4	hypothetical conserved protein	
**CbK_gp286**	+	ccgctaggacaAGGAGGtccc	atg	aggagg	4	hypothetical conserved protein	
**CbK_gp287**	+	acgacaacAGGAGGccccgcc	ttg	aggagg	7	hypothetical conserved protein	
**CbK_gp288**	+	acgcggtcatgAGGAttcgaa	atg	agga	6	hypothetical conserved protein	
**CbK_gp289**	+	ccggcggctgacgggtgcgtc	atg	None	0	hypothetical conserved protein	
**CbK_gp290**	+	gacgacaacgAGGAGGcctcc	atg	aggagg	5	hypothetical conserved protein	
**CbK_gp291**	+	ggactacatcGAGGTgagcct	gtg	gaggt	6	hypothetical conserved protein	
**CbK_gp292**	+	gcttggccgGGAGGTgcagcc	ttg	ggaggt	6	hypothetical conserved protein	

**CbK_gp293**	+	ggcagaaGAGGTcatggcctg	atg	gaggt	9	hypothetical conserved protein	
**CbK_gp294**	+	tctggaacAGGAGaccgccgc	ttg	aggag	8	hypothetical conserved protein	
**CbK_gp295**	+	cggcgggatcaAGGTgagcgc	atg	aggt	6	hypothetical conserved protein	
**CbK_gp296**	+	tttcatcgccGAGGTcctgtc	atg	gaggt	6	hypothetical conserved protein	
**CbK_gp297**	+	acgctgcgaAGGAGcctcgac	atg	aggag	7	hypothetical conserved protein	
**CbK_gp298**	+	ccagatcAGGAGGgccgccgc	atg	aggagg	8	hypothetical conserved protein	
**CbK_gp299**	+	tcgcgacacggcaAGGTacgc	atg	aggt	4	hypothetical conserved protein	
**CbK_gp300**	+	cggcctgcatgcGAGGggctg	atg	gagg	5	hypothetical conserved protein	
**CbK_gp301**	+	gatctacaagccggtgcgctg	atg	None	0	hypothetical conserved protein	
**CbK_gp302**	+	gctcgccaAGGAcgtggtgtg	atg	agga	9	hypothetical conserved protein	
**CbK_gp303**	+	ccgcctGGAGcttgccgccac	atg	ggag	11	hypothetical conserved protein	
**CbK_gp304**	+	cctcggctGGAagtcctcgtc	atg	gga	10	hypothetical conserved protein	
**CbK_gp305**	+	cctggagtatcGGGGtcgggc	gtg	gggg	6	putative HNH homing endonuclease	IPR002711
**CbK_gp306**	+	cgggctagaccGGAGGccatg	atg	ggagg	5	hypothetical conserved protein	
**CbK_gp307**	+	gatccttccGGAGGacgcggc	gtg	ggagg	7	hypothetical conserved protein	
**CbK_gp308**	+	caggctgaaGAGaccgggcgg	atg	gag	9	hypothetical conserved protein	
**CbK_gp309**	+	caccctcaacaAGGAGcccct	gtg	aggag	5	hypothetical conserved protein	
**CbK_gp310**	+	ccggtcctgaGAGGccccaag	atg	gagg	7	hypothetical conserved protein	
**CbK_gp311**	+	taagaatgAGGAaatacatcc	atg	agga	9	hypothetical conserved protein	
**CbK_gp312**	+	aaggcccctcgGGAGccgccc	atg	ggag	6	hypothetical conserved protein	
**CbK_gp313**	+	gccatcgcctGAGacccttcc	atg	gag	8	hypothetical conserved protein	
**CbK_gp314**	+	cgccgacGGGGtggacctgac	gtg	gggg	10	putative RNaseH-like domain protein	IPR012337
**CbK_gp315**	+	cgacccggcGGAGtttccggc	ttg	ggag	8	hypothetical conserved protein	
**CbK_gp316**	+	aagacgacGAGGacGAGGacg	atg	gagg	3	hypothetical conserved protein	
**CbK_gp317**	+	aacgcaccGAGctttaagacc	atg	gag	10	Terminase small subunit	HHpred vs pdb70_18Aug12, hit 3zqp_A, prob 91.6% E=0.1; position relative to terminase large subunit, see text
**CbK_gp318**	+	tcgcgctgaccctagaacgcc	gtg	None	0	Terminase large subunit	IPR004921, IPR007868, IPR006141, IPR003587, IPR006517, SSF51294, G3DSA:2.170.16.10

Table S2. Predicted proteins, gene starts and annotations of *C. crescentus* phage CcrKarma. Upstream sequence is the 25 bp of DNA sequence upstream of the start codon, with the predicted Shine-Dalgarno motif capitalized. S-D is the Shine-Dalgarno motif associated with the gene start, and spacing is the interval (in bp) between the S-D and start codon. Evidence includes detected conserved domains or motifs, BlastP and HHpred hits, and experimental or other evidence as detailed in the text.

Protein name	Strand	Upstream Sequence	Start Codon	S-D	spacing	Predicted product	Evidence
**CcrKarma_gp001**	+	tggcaaatcaAGGAGccggcg	atg	aggag	6	hypothetical conserved protein	
**CcrKarma_gp002**	+	cgatttcaaAGGcttactgcg	atg	agg	9	hypothetical conserved protein	
**CcrKarma_gp003**	+	ccagcccttgtGAGGcctcgg	atg	gagg	6	hypothetical conserved protein	
**CcrKarma_gp004**	+	ccgggcaAGGAGcttcccgcc	atg	aggag	9	hypothetical conserved protein	
**CcrKarma_gp005**	+	gcgccttcGGAGaaaaccgcc	atg	ggag	9	hypothetical conserved protein	
**CcrKarma_gp006**	+	cttcaatgacggcgcggcgtc	gtg	None	0	hypothetical conserved protein	
**CcrKarma_gp007**	+	ggccttcaAGGAGtccgcccc	gtg	aggag	8	hypothetical conserved protein	
**CcrKarma_gp008**	+	ctttcaAGGAttgctcccgtc	atg	agga	11	hypothetical conserved protein	
**CcrKarma_gp009**	+	gccggccaAGGAtcgcagact	atg	agga	9	hypothetical conserved protein	
**CcrKarma_gp010**	+	ccctcgaaAGGAtcgccgacc	atg	agga	9	hypothetical conserved protein	
**CcrKarma_gp011**	+	gacgacggGGAGGcgtgagcc	atg	ggagg	8	hypothetical conserved protein	
**CcrKarma_gp012**	+	cgcatcttGGAGacagcgccc	atg	ggag	9	hypothetical conserved protein	
**CcrKarma_gp013**	+	ccctcgcaAGGAtcgcacccc	atg	agga	9	hypothetical conserved protein	
**CcrKarma_gp014**	+	tcgcctaAGGAGGcccgcccg	atg	aggagg	8	hypothetical conserved protein	
**CcrKarma_gp015**	+	caaccgaAGGccgcctaagcc	atg	agg	11	hypothetical conserved protein	
**CcrKarma_gp016**	+	ccgcaAGGAGGcctaaagccc	atg	aggagg	10	hypothetical conserved protein	
**CcrKarma_gp017**	+	cgccacGGGGgtctaacgccc	gtg	gggg	10	hypothetical novel protein	
**CcrKarma_gp018**	+	cttccttcGGAGcttctgatc	atg	ggag	9	hypothetical conserved protein	
**CcrKarma_gp019**	+	cccctttctGGAtcgctcccc	atg	gga	9	hypothetical conserved protein	
**CcrKarma_gp020**	+	ggtcgcaAGGAGacacccgac	atg	aggag	9	hypothetical conserved protein	
**CcrKarma_gp021**	+	cacgcaatcgAGGAGcccgtc	atg	aggag	6	hypothetical conserved protein	
**CcrKarma_gp022**	+	tccgAGGAcggcaagcacgtc	atg	agga	13	hypothetical conserved protein	
**CcrKarma_gp023**	+	cgcccttGGAGttcccggacg	atg	ggag	10	hypothetical conserved protein	
**CcrKarma_gp024**	+	ctacaAGGTggaaaccctctg	atg	aggt	12	putative lipoprotein	LipoP
**CcrKarma_gp025**	+	atcctgggcctcaAGGTcctg	ctg	aggt	4	hypothetical conserved protein	
**CcrKarma_gp026**	+	tgaaattgactgaAGGTagac	atg	aggt	4	hypothetical novel protein	
**CcrKarma_gp027**	+	catcgcgcgcctGGAGGgagc	atg	ggagg	4	hypothetical conserved protein	
**CcrKarma_gp028**	+	caccagcaacGAGttccattg	atg	gag	8	hypothetical conserved protein	
**CcrKarma_gp029**	+	catccccatcggaGAGGtctg	atg	gagg	4	hypothetical conserved protein	
**CcrKarma_gp030**	+	tggaaatcttcGAGGctacct	atg	gagg	6	hypothetical conserved protein	
**CcrKarma_gp031**	+	ggtcccgctGGAGccgctgcc	gtg	ggag	8	hypothetical conserved protein	
**CcrKarma_gp032**	+	ttgccggcaAGGAGGTggtcc	atg	aggaggt	5	hypothetical conserved protein	
**CcrKarma_gp033**	+	gccccgcaAGGAGcgtgtctt	ttg	aggag	8	hypothetical conserved protein	
**CcrKarma_gp034**	+	agctgccgcctgaAGGAtgcc	atg	agga	4	hypothetical conserved protein	
**CcrKarma_gp035**	+	tcgccccgtaacgAGGAcccc	atg	agga	4	hypothetical conserved protein	
**CcrKarma_gp036**	+	ccctgatcaccGAGGcctgag	atg	gagg	6	hypothetical conserved protein	
**CcrKarma_gp037**	+	gggcatgaagaAGGcgaaacg	atg	agg	7	hypothetical conserved protein	
**CcrKarma_gp038**	+	gcgtGGAcaaccggcggcggc	atg	gga	14	hypothetical conserved protein	
**CcrKarma_gp039**	+	gacatcaAGGAcgccttcgcc	ctg	agga	10	hypothetical conserved protein	
**CcrKarma_gp040**	+	cgaaactgtGAGGcccattcg	atg	gagg	8	hypothetical conserved protein	
**CcrKarma_gp041**	-	gctacctcaAGGAGtagcacc	gtg	aggag	7	hypothetical conserved protein	
**CcrKarma_gp042**	-	gacacttaaGGGGtgtcgatc	atg	gggg	8	putative transglutaminase-like cysteine peptidase	IPR010319
**CcrKarma_gp043**	+	gcgacctcgcAGGccatctat	ctg	agg	8	putative portal protein	HHpred vs pdb70_18Aug12, hit 2jes_A, prob 99.9% E=4.8E-23; positional evidence, see text
**CcrKarma_gp044**	+	cccgaacccgAGGAccgcccg	atg	agga	7	hypothetical conserved protein	
**CcrKarma_gp045**	+	aaccctagcttgtaGAGtaac	atg	gag	4	hypothetical conserved protein	
**CcrKarma_gp046**	+	ccgtcccaagaAGGcgacctg	atg	agg	7	hypothetical conserved protein	
**CcrKarma_gp047**	+	cctccagccttGAGGcgcccc	gtg	gagg	6	hypothetical conserved protein	
**CcrKarma_gp048**	+	gttaaccacGAGGgcttttcc	atg	gagg	8	hypothetical conserved protein	
**CcrKarma_gp049**	+	gctcctttcccaGAGGgctcc	atg	gagg	5	hypothetical conserved protein	
**CcrKarma_gp050**	+	cgaccgcgtgcGGGGcacctg	atg	gggg	6	hypothetical conserved protein	
**CcrKarma_gp051**	+	tccaagcttAGGAGcccgtcc	atg	aggag	7	hypothetical conserved protein	
**CcrKarma_gp052**	+	ggcGAGaagcagtggtggacg	gtg	gag	15	hypothetical conserved protein	
**CcrKarma_gp053**	+	gaccatctacGAGagcacgcg	atg	gag	6	hypothetical conserved protein	
**CcrKarma_gp054**	+	ggtgaccttccgGGAGGagcc	gtg	ggagg	4	hypothetical conserved protein	
**CcrKarma_gp055**	+	tccggtcgAGGAGGgtgacat	ttg	aggagg	7	hypothetical conserved protein	
**CcrKarma_gp056**	+	gcctatctccGGAccccgccg	atg	gga	8	hypothetical conserved protein	
**CcrKarma_gp057**	+	ttaaccatgacgggctccccg	atg	None	0	hypothetical conserved protein	
**CcrKarma_gp058**	+	cctgatgagcGGGGtggacgc	gtg	gggg	7	putative HD-domain/PDEase-like protein	SSF109604
**CcrKarma_gp059**	+	aacgatacGAGaacatccgct	atg	gag	10	hypothetical conserved protein	
**CcrKarma_gp060**	+	gctatctGGAtcgctgagccg	atg	gga	11	hypothetical conserved protein	
**CcrKarma_gp061**	+	cctgccgaagaAGGccgcctg	atg	agg	7	putative DHH phosphoesterase protein	SSF64182
**CcrKarma_gp062**	+	caagcgtcgggcGGGGacggc	gtg	gggg	5	hypothetical conserved protein	
**CcrKarma_gp063**	+	tctgccaacAGGAGatcgccg	gtg	aggag	7	hypothetical conserved protein	
**CcrKarma_gp064**	+	ttccagcacatctAGGAcccc	atg	agga	4	hypothetical conserved protein	
**CcrKarma_gp065**	+	gattacGAGGcctatcaccac	gtg	gagg	11	hypothetical conserved protein	
**CcrKarma_gp066**	+	ttcgctgcGAGGccatttctg	atg	gagg	9	hypothetical conserved protein	
**CcrKarma_gp067**	+	cAGGTctgcgtctcccactac	ctg	aggt	16	putative HNH endonuclease	IPR002711
**CcrKarma_gp068**	+	ccgtgggaaGAGagaaactgt	atg	gag	7	hypothetical conserved protein	
**CcrKarma_gp069**	+	aacaccaagAGGAGcaagcca	atg	aggag	7	putative major capsid protein	experimental evidence, see text
**CcrKarma_gp070**	+	atctaagcAGGAaatccccag	atg	agga	9	putative minor capsid protein	experimental evidence, see text
**CcrKarma_gp071**	+	ccccgaaacGGAaaatccccc	atg	gga	9	hypothetical conserved protein	
**CcrKarma_gp072**	+	aagcccacggcggcctaagcc	atg	None	0	hypothetical conserved protein	
**CcrKarma_gp073**	+	atcacccAGGTgaactaagcc	gtg	aggt	10	hypothetical conserved protein	
**CcrKarma_gp074**	+	tGGAccgcacgcgctggcgcc	ctg	gga	17	hypothetical conserved protein	
**CcrKarma_gp075**	+	ttcacgaactaagGGAcggcc	atg	gga	5	hypothetical conserved protein	

**CcrKarma_gp076**	+	aacccccAGGAGccctgaacc	atg	aggag	9	hypothetical conserved protein	
**CcrKarma_gp077**	+	gccgcctcaAGGgtcttcgcg	ttg	agg	9	putative lectin-like domain protein	IPR013320, IPR008985
**CcrKarma_gp078**	+	cagacctGGAGGcctaagacc	atg	ggagg	9	hypothetical conserved protein	
**CcrKarma_gp079**	+	ttccgctcgcaGGGGcgccgc	gtg	gggg	6	hypothetical conserved protein	
**CcrKarma_gp080**	+	tctGGAGtttcgagcgccatc	atg	ggag	14	hypothetical conserved protein	
**CcrKarma_gp081**	+	cgcaccgccAGGgctttccca	atg	agg	9	hypothetical conserved protein	
**CcrKarma_gp082**	+	ctgcggtcgcgcGAGGcttct	atg	gagg	5	hypothetical conserved protein	
**CcrKarma_gp083**	+	ggcggccgttcGAGagccatc	atg	gag	5	hypothetical conserved protein	
**CcrKarma_gp084**	-	atccagccgctGGAGatactt	atg	ggag	6	hypothetical conserved protein	
**CcrKarma_gp085**	+	agtcGGGGaccgagagccgtc	atg	gggg	13	hypothetical conserved protein	
**CcrKarma_gp086**	+	tcctgggtGGAGttttcccta	atg	ggag	9	hypothetical conserved protein	
**CcrKarma_gp087**	+	cgccatcccagagGGAGcgac	gtg	ggag	4	putative PhoH-like protein	IPR003714, SSF52540, G3DSA:3.40.50.300
**CcrKarma_gp088**	+	cAGGAcggcaAGGAacgtttt	ctg	agga	7	hypothetical conserved protein	
**CcrKarma_gp089**	+	gtgcgtgatgtcGAGagcggc	gtg	gag	4	hypothetical conserved protein	
**CcrKarma_gp090**	+	cagcacgcGGAagaagacccc	gtg	gga	10	hypothetical conserved protein	
**CcrKarma_gp091**	+	ctcgattaaaGAGGTgttcgg	atg	gaggt	6	hypothetical conserved protein	
**CcrKarma_gp092**	+	gttctggctgggaGAGGactg	atg	gagg	4	hypothetical conserved protein	
**CcrKarma_gp093**	+	ccgtGGAttggtgacctgccc	atg	gga	14	putative major tail tube protein	experimental evidence, see text
**CcrKarma_gp094**	+	cccgatacGGAatccccgaac	atg	gga	10	putative pre-tape measure chaperone protein	HHpred vs pdb70_18Aug12, hit 2ob9_A, prob 89.3% E=0.82; positional evidence, see text
**CcrKarma_gp095**	+	cccgatacGGAatccccgaac	atg	gga	10	putative pre-tape measure chaperone protein, frameshifted version	positional evidence, see text; slippery sequence AAAAAAC
**CcrKarma_gp096**	+	gcGGGGccgccgataggatca	atg	gggg	15	putative tail tape measure protein	IPR007921, IPR013491, IPR013423; experimental evidence, see text
**CcrKarma_gp097**	+	tcctttcatggttaatgcccg	atg	None	0	hypothetical conserved protein	IPR011740
**CcrKarma_gp098**	+	ctggcaaGAGGccctgacgcc	gtg	gagg	10	putative tail protein	IPR019228, IPR018964, IPR011928; experimental evidence, see text
**CcrKarma_gp099**	+	gcccaacgctccGGAGcagga	atg	ggag	5	putative NlpC/P60 family cell wall peptidase	IPR000064, SSF54001, G3DSA:3.90.1720.10
**CcrKarma_gp100**	+	ccccggcgttgAGGActaatc	atg	agga	6	putative tail protein	IPR007110; experimental evidence, see text
**CcrKarma_gp101**	+	tgtcataaaagtcgtggaacg	atg	None	0	hypothetical conserved protein	
**CcrKarma_gp102**	+	cctcaAGGgcgtcatcctgca	atg	agg	13	putative tail protein	IPR000421, IPR008979, G3DSA:2.60.120.260; experimental evidence, see text
**CcrKarma_gp103**	+	accctcaagGGAatccgatag	atg	gga	9	hypothetical conserved protein	
**CcrKarma_gp104**	+	tcttcatgctcGGAGcctgac	atg	ggag	6	hypothetical conserved protein	
**CcrKarma_gp105**	+	tcagcacttccGGAattccac	atg	gga	7	putative endolysin	IPR002196, SSF53955, G3DSA:1.10.530.40
**CcrKarma_gp106**	+	gggcttcaAGGgctgctgatc	atg	agg	10	putative inner membrane spanin component	N-terminal TMD, outer membrane spanin component embedded in +1 frame, see text
**CcrKarma_gp107**	+	agtaccaaGAGGTtattcacg	atg	gaggt	8	putative outer membrane spanin component	lipoprotein embedded in inner membrane spanin component, see text
**CcrKarma_gp108**	+	caagctcaaGGGGaagacgca	gtg	gggg	8	putative holin protein	predicted 4 TMDs, see text
**CcrKarma_gp109**	+	cgctatcacGGAGtagggacc	atg	ggag	8	hypothetical conserved protein	
**CcrKarma_gp110**	-	cgacggacggAGGAGaacgaa	atg	aggag	6	putative HTH domain DNA-binding protein	IPR001387, IPR010982, G3DSA:1.10.260.40
**CcrKarma_gp111**	-	ttaaccaGAGGcccgaaaata	atg	gagg	10	putative dUTP pyrophosphatase	IPR008181, IPR008180, SSF51283, PTHR11241, G3DSA:2.70.40.10
**CcrKarma_gp112**	-	tccttttcctGAGacccсttc	atg	gag	8	putative ribonucleoside diphosphate reductase beta subunit	IPR012348, IPR012335, IPR000358, IPR002109, IPR009078, IPR012336, IPR011767
**CcrKarma_gp113**	-	tcgacgcGAGaacGAGacccc	atg	gag	5	putative ribonucleoside diphosphate reductase alpha subunit	IPR006141, IPR003587, IPR000788, IPR013509, IPR008926, SSF51294, SSF51998, G3DSA:2.170.16.10, G3DSA:3.20.70.20
**CcrKarma_gp114**	-	cggtggtGGGGgtggctggcg	ttg	gggg	9	hypothetical conserved protein	
**CcrKarma_gp115**	-	tcctcatgcagcAGGAatccc	atg	agga	5	hypothetical conserved protein	
**CcrKarma_gp116**	-	acgacatcaccGGAGccgagc	atg	ggag	6	hypothetical conserved protein	
**CcrKarma_gp117**	-	gacgccgcccAGGActgatcc	atg	agga	7	putative thymidylate synthase	IPR003669
**CcrKarma_gp118**	-	atccttcgctAGGAGaacccc	atg	aggag	6	putative dNMP kinase	HHpred vs pdb70_18Aug12, hit 2grj_A, prob 99.9% E=3.6E-27; SSF52540, G3DSA:3.40.50.300
**CcrKarma_gp119**	-	aagatcaAGGAcgggcaaccc	ctg	agga	10	putative RecD-like helicase	HHpred vs pdb70_18Aug12, hit 3e1s_A, prob 100% E=1.3E-45; SSF52540, G3DSA:3.40.50.300
**CcrKarma_gp120**	-	acagaaaatAGGTatcagcaa	atg	aggt	8	hypothetical conserved protein	
**CcrKarma_gp121**	-	aGAGaaatgtgacggtgcgcg	atg	gag	17	hypothetical conserved protein	
**CcrKarma_gp122**	-	caagatgcgGGAGaaggccgc	gtg	ggag	8	hypothetical conserved protein	
**CcrKarma_gp123**	-	actcccgaacGAGatcgcccg	atg	gag	8	putative exoribonuclease, Pol III-like	IPR006055, IPR013520, IPR012337, G3DSA:3.30.420.10
**CcrKarma_gp124**	-	cacctgggcGGAGacgcactg	atg	ggag	8	hypothetical conserved protein	
**CcrKarma_gp125**	-	atcaaatcctcaAGGTgctcc	atg	aggt	5	putative Pol I DNA polymerase, T7-like	IPR001098, IPR012337, SSF56672, PTHR10133, G3DSA:1.10.150.20, G3DSA:1.20.1060.10, G3DSA:3.30.420.10, G3DSA:3.30.70.370; see text
**CcrKarma_gp126**	-	acatccgttaaccatagagcc	atg	None	0	putative DNA cytosine methyltransferase	IPR001525, IPR018117, SSF53335, PTHR10629:SF11, G3DSA:3.40.50.150, G3DSA:3.90.120.10
**CcrKarma_gp127**	-	cgaaagccttcAGGAGtaatc	atg	aggag	5	hypothetical conserved protein	
**CcrKarma_gp128**	-	gcgccaaaccGGAGcttctct	atg	ggag	7	putative T5 A1-like protein	blastP to T5 A1 (AAU05157), E=4.81E-90, 34.2% Dice identity; HHpred vs pdb70_18Aug12, hit 1g2h_A, prob 94.0% E=0.05
**CcrKarma_gp129**	-	tccggatatgccAGGAccccg	ctg	agga	5	putative DNA methylase	IPR002052, SSF53335, G3DSA:3.40.50.150
**CcrKarma_gp130**	-	aggctccgttcgcgGGActtg	ctg	gga	4	hypothetical conserved protein	
**CcrKarma_gp131**	-	aaagatcgctatcgttgtcgc	atg	None	0	hypothetical conserved protein	
**CcrKarma_gp132**	-	catcgcaaAGGTcatggacac	gtg	aggt	9	hypothetical conserved protein	
**CcrKarma_gp133**	-	cgttaaccacgtgcggcgccc	atg	None	0	putative DNA helicase	IPR014001, IPR001650, IPR000330, IPR014021, SSF52540, PTHR10799, G3DSA:3.40.50.300
**CcrKarma_gp134**	+	ccggtcacaAGGAGaactatc	atg	aggag	7	hypothetical conserved protein	
**CcrKarma_gp135**	-	ggaaatcaAGGAagctgcgtg	atg	agga	9	hypothetical conserved protein	
**CcrKarma_gp136**	-	ccccgccgcaaAGGccccctg	atg	agg	7	hypothetical conserved protein	
**CcrKarma_gp137**	-	acccacGAGGTctcgcgttac	ctg	gaggt	10	hypothetical conserved protein	
**CcrKarma_gp138**	-	cgccttttcaAGGAtcgcccg	atg	agga	7	hypothetical conserved protein	
**CcrKarma_gp139**	-	gccggtctcaAGGAGacgacc	atg	aggag	6	Putative rIIb-like protein	PD130832
**CcrKarma_gp140**	-	gcctgcctttcGGAGtttgcc	atg	ggag	6	putative rIIa-like protein	IPR003594
**CcrKarma_gp141**	+	ggcaaactccgaaAGGcAGGc	ttg	agg	1	hypothetical conserved protein	
**CcrKarma_gp142**	+	gcctGAGGcatgctcgtattc	atg	gagg	13	hypothetical conserved protein	
**CcrKarma_gp143**	+	cgtatggtacgcgAGGAtgac	atg	agga	4	hypothetical conserved protein	
**CcrKarma_gp144**	+	caccgacgggcgcgtcctcca	ttg	None	0	hypothetical conserved protein	
**CcrKarma_gp145**	+	acgacaacgaAGGAGGTtctc	atg	aggaggt	4	putative tyrosine recombinase	IPR013762, IPR002104, IPR011010
**CcrKarma_gp146**	+	gtcctgcgatAGGAGGgcgcg	atg	aggagg	5	hypothetical conserved protein	
**CcrKarma_gp147**	+	ccgctagtccAGGAGtacagt	ttg	aggag	6	hypothetical conserved protein	
**CcrKarma_gp148**	+	acccccgtcccgacAGGcttc	atg	agg	4	hypothetical conserved protein	
**CcrKarma_gp149**	+	atcgctcctGGGGccttttga	gtg	gggg	8	hypothetical conserved protein	
**CcrKarma_gp150**	+	tcctgtacggtaagGGAaaac	atg	gga	4	hypothetical conserved protein	
**CcrKarma_gp151**	+	acgattcGGAGtattcatgaa	atg	ggag	10	putative HTH domain protein	IPR001387, G3DSA:1.10.260.40
**CcrKarma_gp152**	+	cctgtattcctGAGGTcccgc	atg	gaggt	5	putative tRNA amidotransferase domain protein	IPR019004, IPR003789
**CcrKarma_gp153**	-	actgatcgaGGGGaagttgag	atg	gggg	8	putative DNA ligase	IPR012340, IPR016027, SSF56091, G3DSA:3.30.470.30
**CcrKarma_gp154**	-	tcgatccGGAGattttcggcg	atg	ggag	10	hypothetical conserved protein	

**CcrKarma_gp155**	-	gccatcccctctatctccgag	gtg	None	0	hypothetical conserved protein	
**CcrKarma_gp156**	-	gtttcAGGgcaaatgtcAGGa	atg	agg	1	DUF1643 protein	IPR012441
**CcrKarma_gp157**	-	gcctcgcccttgaAGGAggca	atg	agga	4	hypothetical conserved protein	
**CcrKarma_gp158**	-	gAGGccaagaagcgggccacg	gtg	agg	17	hypothetical conserved protein	
**CcrKarma_gp159**	-	ctGGAtcgccccggcggcctg	atg	gga	16	hypothetical conserved protein	
**CcrKarma_gp160**	-	gcttttgAGGAGtttggacgt	atg	aggag	9	hypothetical conserved protein	
**CcrKarma_gp161**	-	agcaggactAGGAGaaagacc	atg	aggag	7	hypothetical conserved protein	
**CcrKarma_gp162**	-	cgacgccaAGGAcgtctgcca	atg	agga	9	hypothetical conserved protein	
**CcrKarma_gp163**	-	ggccggctacGAGatcgcctg	atg	gag	8	putative lipoprotein	LipoP
**CcrKarma_gp164**	-	cgcttaaaGGGGgacctcatc	atg	gggg	8	putative nicotinate phosphoribosyltransferase	IPR015977, IPR002638, PTHR11098
**CcrKarma_gp165**	-	cagaatgaGGGGtaagcgata	gtg	gggg	9	putative NUDIX hydrolase domain protein	IPR014729, IPR000086, IPR015797, SSF52374, PS51462, PTHR22769
**CcrKarma_gp166**	-	caaagcgtagtgtcgcgaggc	atg	None	0	hypothetical conserved protein	
**CcrKarma_gp167**	-	cgcaacgcctgGGActctctg	atg	gga	7	putative peptidyl-tRNA hydrolase	IPR002833
**CcrKarma_gp168**	-	agcgccggccgccGAGGgctg	atg	gagg	4	hypothetical conserved protein	
**CcrKarma_gp169**	-	taacctccagcAGGAGaccgc	atg	aggag	5	hypothetical conserved protein	
**CcrKarma_gp170**	-	acaacgccGGAGGccctttttc	atg	ggagg	8	hypothetical conserved protein	
**CcrKarma_gp171**	-	ccccgatctccGAGaagcccc	atg	gag	7	putative HD-domain/PDEase-like protein	SSF109604
**CcrKarma_gp172**	-	gggagaattcctGAGGgtcgc	atg	gagg	5	hypothetical conserved protein	
**CcrKarma_gp173**	-	acgcccagaAGGActaatcct	atg	agga	8	putative DNA-binding domain protein	IPR016177, SSF54060
**CcrKarma_gp174**	-	gaggcctcGGAGaagtaagcc	atg	ggag	9	hypothetical conserved protein	
**CcrKarma_gp175**	-	tcttgagaAGGAacaaccctg	atg	agga	9	hypothetical conserved protein	
**CcrKarma_gp176**	-	tcaacaAGGgcaagaacgccg	atg	agg	12	hypothetical conserved protein	
**CcrKarma_gp177**	-	caagcAGGcggcgggtctgct	ttg	agg	13	hypothetical conserved protein	
**CcrKarma_gp178**	-	gctgttcgactGGAGaaaatc	gtg	ggag	6	hypothetical conserved protein	
**CcrKarma_gp179**	-	ctctgcgtgaAGGgctAGGcc	atg	agg	2	hypothetical conserved protein	
**CcrKarma_gp180**	-	acaccacagaAGGAGcccgcc	gtg	aggag	6	putative RtcB-like protein	IPR001233
**CcrKarma_gp181**	-	gtacccctcaAGGTgatcccc	atg	aggt	7	hypothetical conserved protein	
**CcrKarma_gp182**	-	aacaaagcaAGGAGcaacccc	ttg	aggag	7	putative band 7 lipoprotein	IPR001107
**CcrKarma_gp183**	-	actcctgaaAGGccgcgccca	atg	agg	9	hypothetical conserved protein	
**CcrKarma_gp184**	-	gccaccGAGGccatggacgac	atg	gagg	11	hypothetical conserved protein	
**CcrKarma_gp185**	-	gacgacaacgaAGGAGacccc	atg	aggag	5	hypothetical conserved protein	
**CcrKarma_gp186**	-	caggatGGAGGccgccgccaa	atg	ggagg	10	hypothetical conserved protein	
**CcrKarma_gp187**	-	tccgagccaAGGAGcgctgat	ttg	aggag	7	hypothetical conserved protein	
**CcrKarma_gp188**	-	cgacagcgagAGGAGccgatc	atg	aggag	6	hypothetical conserved protein	
**CcrKarma_gp189**	-	gcactctggaaaAGGAccacc	atg	agga	5	hypothetical conserved protein	
**CcrKarma_gp190**	-	ggtcctctcgGGAGacaatcc	atg	ggag	7	hypothetical conserved protein	
**CcrKarma_gp191**	-	tctgaaAGGctcgacctcctg	atg	agg	12	hypothetical conserved protein	
**CcrKarma_gp192**	-	cgttccaactcgcAGGTctcc	gtg	aggt	4	hypothetical conserved protein	
**CcrKarma_gp193**	-	cctgagcctGGAGaagcacct	gtg	ggag	8	hypothetical conserved protein	
**CcrKarma_gp194**	-	actggcgaAGGTgtcggacca	atg	aggt	9	hypothetical conserved protein	
**CcrKarma_gp195**	-	acaaccgcagAGGAGaccctc	atg	aggag	6	hypothetical conserved protein	
**CcrKarma_gp196**	-	cgGGAtgcgttccgggtcgag	atg	gga	16	hypothetical conserved protein	
**CcrKarma_gp197**	-	gcccgagaaAGGcgaAGGccc	atg	agg	3	hypothetical conserved protein	
**CcrKarma_gp198**	-	cgccgacGGAGagctactgcg	gtg	ggag	10	putative winged-helix HTH DNA binding protein	IPR011991
**CcrKarma_gp199**	-	gcccgagcAGGTcccttggac	atg	aggt	9	hypothetical novel protein	
**CcrKarma_gp200**	-	ccacccgcgccgcccgaggag	gtg	None	0	hypothetical conserved protein	
**CcrKarma_gp201**	-	cagcgccggcAGGgtccccgc	atg	agg	8	hypothetical conserved protein	
**CcrKarma_gp202**	-	aagaggtcgAGGAGGTcgccg	atg	aggaggt	5	hypothetical conserved protein	
**CcrKarma_gp203**	-	gacgacaacgAGGAGGccctg	atg	aggagg	5	putative ArdC-like antirestriction protein	IPR017113, IPR013610
**CcrKarma_gp204**	-	cgcggcgataatGAGcgcctg	atg	gag	6	hypothetical conserved protein	
**CcrKarma_gp205**	-	ggacctGGAGaacaaccgctg	atg	ggag	11	hypothetical conserved protein	
**CcrKarma_gp206**	-	gggccggGGAGGgcgtgcatc	gtg	ggagg	9	hypothetical conserved protein	
**CcrKarma_gp207**	-	cgcctgccaGAGcctcaagtc	atg	gag	9	hypothetical conserved protein	
**CcrKarma_gp208**	-	aacgacaccagggGGAGagcc	gtg	ggag	4	putative acyl carrier protein	IPR009081, IPR006163, PTHR20863
**CcrKarma_gp209**	-	tcgacaacggcaAGGAcggct	atg	agga	5	putative HNH endonuclease	IPR002711
**CcrKarma_gp210**	-	ctcattcgttaAGGAtcgacc	atg	agga	6	hypothetical conserved protein	
**CcrKarma_gp211**	-	ccGGAactccaggcGGAgttc	atg	gga	4	hypothetical conserved protein	
**CcrKarma_gp212**	-	tggGGAGaagctgaagtgagc	atg	ggag	14	hypothetical conserved protein	
**CcrKarma_gp213**	-	cgccttcGAGGTgcaagcctg	atg	gaggt	9	hypothetical conserved protein	
**CcrKarma_gp214**	-	tccggacctcGGAGaaggccc	gtg	ggag	7	hypothetical conserved protein	
**CcrKarma_gp215**	-	gcggttcgAGGAGatcgaggc	ttg	aggag	8	hypothetical conserved protein	
**CcrKarma_gp216**	-	gacgacaacggaAGGAaccct	atg	agga	5	hypothetical conserved protein	
**CcrKarma_gp217**	-	cttcgccaaGGGGacctgacc	atg	gggg	8	hypothetical conserved protein	
**CcrKarma_gp218**	-	ccaaGGGGgacggctactaac	atg	gggg	12	hypothetical conserved protein	
**CcrKarma_gp219**	-	gcgcgaaccgaAGGTcgactg	atg	aggt	6	hypothetical conserved protein	
**CcrKarma_gp220**	-	gacgacaacaAGGAGGcctcc	atg	aggagg	5	hypothetical conserved protein	
**CcrKarma_gp221**	-	ctacgtGGAGagccgggcggc	atg	ggag	11	hypothetical conserved protein	
**CcrKarma_gp222**	-	cgcgcaatttccGGAGtgacc	atg	ggag	5	hypothetical conserved protein	SSF56784
**CcrKarma_gp223**	-	ggcctgcgcgGGAGccctgtg	atg	ggag	7	hypothetical conserved protein	
**CcrKarma_gp224**	-	gagactagggtGGGGatgcga	ttg	gggg	6	hypothetical conserved protein	
**CcrKarma_gp225**	-	gatcaagtcGAGGccctgagc	atg	gagg	8	hypothetical conserved protein	
**CcrKarma_gp226**	-	ggctcccgtacAGGAtcacct	atg	agga	6	hypothetical conserved protein	
**CcrKarma_gp227**	-	aaaGGGGccgtctatggctcc	ctg	gggg	14	hypothetical conserved protein	
**CcrKarma_gp228**	-	agcagaAGGAGGaagccgctc	atg	aggagg	9	putative HTH domain DNA-binding protein	IPR010982, IPR001387, G3DSA:1.10.260.40
**CcrKarma_gp229**	-	aatcgcccAGGgcgccgcctg	atg	agg	10	hypothetical conserved protein	
**CcrKarma_gp230**	-	acaacgacagaAGGAGGccgc	gtg	aggagg	4	hypothetical conserved protein	
**CcrKarma_gp231**	-	gaggcgggcgaGGGGcacgtc	gtg	gggg	6	hypothetical conserved protein	
**CcrKarma_gp232**	-	cactgtccggaAGGTcgagcc	atg	aggt	6	hypothetical conserved protein	
**CcrKarma_gp233**	-	ggtctatcgccGGAGGgcctg	atg	ggagg	5	putative Pol I DNA polymerase	IPR001098, IPR002298, SSF56672, G3DSA:3.30.70.370

**CcrKarma_gp234**	-	agatcaAGGAGtagtgcaagc	gtg	aggag	10	putative GcrA-like cell cycle regulator	IPR011681; see text
**CcrKarma_gp235**	-	gccgccaaattcGAGGggaag	atg	gagg	5	hypothetical conserved protein	
**CcrKarma_gp236**	+	cgacaacgaaAGGAGcctgcc	atg	aggag	6	hypothetical conserved protein	
**CcrKarma_gp237**	+	cgccgcGAGGctcgcccaatg	atg	gagg	11	hypothetical conserved protein	
**CcrKarma_gp238**	+	caagatcGAGGaaGAGGtctg	atg	gagg	4	hypothetical conserved protein	
**CcrKarma_gp239**	+	cattcgcgtgttGGGGctcgg	atg	gggg	5	hypothetical conserved protein	
**CcrKarma_gp240**	+	caagctcaagaaAGGTcgcct	atg	aggt	5	hypothetical conserved protein	
**CcrKarma_gp241**	+	cattctcgactAGGAGGaccc	atg	aggagg	4	hypothetical conserved protein	
**CcrKarma_gp242**	+	tcgatttcctggcgggcgccg	atg	None	0	hypothetical conserved protein	
**CcrKarma_gp243**	+	gtttctggaAGGTcaggactg	atg	aggt	8	hypothetical conserved protein	
**CcrKarma_gp244**	+	agcgcgagccgcAGGActgac	atg	agga	5	hypothetical conserved protein	
**CcrKarma_gp245**	+	cgacagcgaaAGGAGcctgcc	ttg	aggag	6	hypothetical conserved protein	
**CcrKarma_gp246**	+	tcggatgctGGAGGTcgcggc	ttg	ggaggt	6	hypothetical conserved protein	
**CcrKarma_gp247**	+	cgccgtggAGGAGatgcgccg	gtg	aggag	8	hypothetical conserved protein	
**CcrKarma_gp248**	+	cgcagacttcaAGGgcaagcg	atg	agg	7	hypothetical conserved protein	
**CcrKarma_gp249**	+	gccgccGGAGGcagacccgac	gtg	ggagg	10	hypothetical conserved protein	
**CcrKarma_gp250**	+	tcaagctGGAGaccgtctgac	atg	ggag	10	hypothetical conserved protein	
**CcrKarma_gp251**	+	acgacaacgagAGGAGttccc	atg	aggag	5	hypothetical conserved protein	
**CcrKarma_gp252**	+	cacggaatgGGAGattttcga	atg	ggag	8	hypothetical conserved protein	
**CcrKarma_gp253**	+	ggcctGGAGatcatcgcctag	atg	ggag	12	hypothetical conserved protein	
**CcrKarma_gp254**	+	acctcggacgAGGAGGactaa	atg	aggagg	5	hypothetical conserved protein	
**CcrKarma_gp255**	+	gaccccGGAGtccaagaccga	atg	ggag	11	hypothetical conserved protein	
**CcrKarma_gp256**	+	caacgccctgGGAGccgccaa	atg	ggag	7	hypothetical conserved protein	
**CcrKarma_gp257**	+	ggtcagccAGGAcatgccgtc	gtg	agga	9	hypothetical conserved protein	
**CcrKarma_gp258**	+	gacgacaacgAGGAGGcttcg	atg	aggagg	5	hypothetical conserved protein	
**CcrKarma_gp259**	+	gtcaaagAGGAGGcctgagcc	ttg	aggagg	8	hypothetical conserved protein	
**CcrKarma_gp260**	+	ggcgccctacGAGGccccgca	atg	gagg	7	hypothetical conserved protein	
**CcrKarma_gp261**	+	ccggcgAGGAGGTccgctgac	atg	aggaggt	8	hypothetical conserved protein	
**CcrKarma_gp262**	+	tggacgacaacAGGAGGgacc	atg	aggagg	4	hypothetical conserved protein	
**CcrKarma_gp263**	+	ctacgtcaAGGAcctgcgtcg	atg	agga	9	hypothetical conserved protein	
**CcrKarma_gp264**	+	tcagcgtgGGAGtggttgctg	atg	ggag	9	hypothetical conserved protein	
**CcrKarma_gp265**	+	gacccaGGGGgttcccgaggc	atg	gggg	10	hypothetical conserved protein	
**CcrKarma_gp266**	+	cctcgacgcgAGGAaaagctg	atg	agga	7	hypothetical conserved protein	
**CcrKarma_gp267**	+	aggccctgaagGGAGagaccg	atg	ggag	6	hypothetical conserved protein	
**CcrKarma_gp268**	+	gaccaaGGGGgtgcGGGGcaa	atg	gggg	3	hypothetical conserved protein	
**CcrKarma_gp269**	+	ttgtcttGAGaaagccaacgc	atg	gag	11	DUF1937 protein	IPR015235, SSF52309
**CcrKarma_gp270**	+	gagcaccagGGAGattgaagc	gtg	ggag	8	hypothetical conserved protein	
**CcrKarma_gp271**	+	ggtgtcccaGAGGTaccagcg	atg	gaggt	7	hypothetical conserved protein	
**CcrKarma_gp272**	+	ccggcgcctatGGAGagagcc	atg	ggag	6	putative TAT signal protein	IPR006311; PRED-TAT
**CcrKarma_gp273**	+	tctcgacatggcgaaaagcgc	atg	None	0	hypothetical conserved protein	
**CcrKarma_gp274**	+	acgtcaacaAGGAGcccaccg	atg	aggag	7	hypothetical conserved protein	
**CcrKarma_gp275**	+	agaagcccggcgggccgagtg	atg	None	0	hypothetical conserved protein	
**CcrKarma_gp276**	+	gcggtcgaaatcctGGAgacc	ctg	gga	4	hypothetical conserved protein	
**CcrKarma_gp277**	+	gccgctcgacgggtGGAacgg	atg	gga	4	hypothetical conserved protein	
**CcrKarma_gp278**	+	cgaggaagAGGAGcgctgctg	atg	aggag	8	putative Ser/Thr protein phosphatase	IPR004843, IPR006186, SSF56300, PTHR11668, G3DSA:3.60.21.10
**CcrKarma_gp279**	+	gttgatccgcgAGGAcgacgc	atg	agga	6	hypothetical conserved protein	
**CcrKarma_gp280**	+	gacgacaacgAGGAGGgccac	atg	aggagg	5	hypothetical conserved protein	
**CcrKarma_gp281**	+	catgaccaaGAGccgcttctg	atg	gag	9	hypothetical conserved protein	
**CcrKarma_gp282**	+	gatcatcctgGGAGacgccgc	atg	ggag	7	hypothetical conserved protein	
**CcrKarma_gp283**	+	ccgcttcgAGGAGGcccgccc	atg	aggagg	7	hypothetical conserved protein	
**CcrKarma_gp284**	+	cgacgacaacgAGGAGGcccc	atg	aggagg	4	hypothetical conserved protein	
**CcrKarma_gp285**	+	ctcgaaGAGGTcgagatcgcc	ctg	gaggt	10	hypothetical novel protein	
**CcrKarma_gp286**	+	gcgctgaccaAGGgctggtag	atg	agg	8	hypothetical conserved protein	
**CcrKarma_gp287**	+	acgcgcgacgGGAGGgctaac	atg	ggagg	6	hypothetical conserved protein	
**CcrKarma_gp288**	+	tccgaaaGAGGccttaagtac	atg	gagg	10	hypothetical conserved protein	
**CcrKarma_gp289**	+	aacggaAGGAcctgaaagacc	atg	agga	11	hypothetical conserved protein	
**CcrKarma_gp290**	+	acaacgccGGAGGccctttc	atg	ggagg	8	hypothetical conserved protein	
**CcrKarma_gp291**	+	gcctggaAGGcggctgacatc	ctg	agg	11	hypothetical conserved protein	
**CcrKarma_gp292**	+	acgccaagaAGGccgcctgac	atg	agg	9	hypothetical conserved protein	
**CcrKarma_gp293**	+	ccagatcaAGGAGGcccagtc	ttg	aggagg	7	hypothetical conserved protein	
**CcrKarma_gp294**	+	aatcagcaataGGGGgccagc	atg	gggg	5	hypothetical conserved protein	
**CcrKarma_gp295**	+	ctgggccGAGGgctcgccgcg	atg	gagg	10	hypothetical conserved protein	
**CcrKarma_gp296**	+	cggacatagAGGAGcaacccg	atg	aggag	7	hypothetical conserved protein	
**CcrKarma_gp297**	+	tcgccgcctgAGGAGGacccg	atg	aggagg	5	hypothetical conserved protein	
**CcrKarma_gp298**	+	gcttgagaAGGcgttcggcca	atg	agg	10	hypothetical conserved protein	
**CcrKarma_gp299**	+	ccaagcctcGGGGctgttcca	atg	gggg	8	hypothetical conserved protein	
**CcrKarma_gp300**	+	caccgactactAGGAGGtccc	atg	aggagg	4	hypothetical conserved protein	
**CcrKarma_gp301**	+	ccgctaggacaAGGAGGtccc	atg	aggagg	4	hypothetical conserved protein	
**CcrKarma_gp302**	+	acgacaacAGGAGGccccgcc	ttg	aggagg	7	hypothetical conserved protein	
**CcrKarma_gp303**	+	ttgcggtcatgAGGAttcgaa	atg	agga	6	hypothetical conserved protein	
**CcrKarma_gp304**	+	atcGGGGtctgggatttctcg	ctg	gggg	14	hypothetical conserved protein	
**CcrKarma_gp305**	+	gacgacaacgAGGAGGccccc	atg	aggagg	5	hypothetical conserved protein	
**CcrKarma_gp306**	+	ggactacatcGAGGTggccct	gtg	gaggt	6	hypothetical conserved protein	
**CcrKarma_gp307**	+	ccttagccgGGAGGcgcagcc	ttg	ggagg	7	hypothetical conserved protein	
**CcrKarma_gp308**	+	ggcggcagaaGAGGccatggc	ctg	gagg	7	hypothetical conserved protein	
**CcrKarma_gp309**	+	ggaacAGGAGaccgccgcctg	atg	aggag	11	hypothetical conserved protein	
**CcrKarma_gp310**	+	gatcgcgcgactGGAGaacgc	atg	ggag	5	hypothetical conserved protein	
**CcrKarma_gp311**	+	cggcgggatcaAGGTgagcgc	atg	aggt	6	hypothetical conserved protein	
**CcrKarma_gp312**	+	tttcatcgccGAGGccctgtc	atg	gagg	7	hypothetical conserved protein	

**CcrKarma_gp313**	+	acgctgcgaAGGAGcctcgac	ttg	aggag	7	hypothetical conserved protein	
**CcrKarma_gp314**	+	ccagatcAGGAaggccgccgc	atg	agga	10	hypothetical conserved protein	
**CcrKarma_gp315**	+	tcgcgacacggcaAGGTacgc	atg	aggt	4	hypothetical conserved protein	
**CcrKarma_gp316**	+	cggcctgcatgcGAGGggctg	atg	gagg	5	hypothetical conserved protein	
**CcrKarma_gp317**	+	gatctacaagccggtgcgctg	atg	None	0	hypothetical conserved protein	
**CcrKarma_gp318**	+	gctcgccaAGGAcgtggtgtg	atg	agga	9	hypothetical conserved protein	
**CcrKarma_gp319**	+	ccgcctGGAGcttgccgccac	atg	ggag	11	hypothetical conserved protein	
**CcrKarma_gp320**	+	cctcggctGGAagtcctcgtc	atg	gga	10	hypothetical conserved protein	
**CcrKarma_gp321**	+	cctggagtatcGGGGtcgggc	gtg	gggg	6	putative HNH homing endonuclease	IPR002711
**CcrKarma_gp322**	+	aaccgggctagaccGGAggcc	atg	gga	4	hypothetical conserved protein	
**CcrKarma_gp323**	+	gatccttccGGAGGacgcggc	gtg	ggagg	7	hypothetical conserved protein	
**CcrKarma_gp324**	+	cagactgaaGAGGcccggcgg	atg	gagg	8	hypothetical conserved protein	
**CcrKarma_gp325**	+	caccctcaacaAGGAGcccct	gtg	aggag	5	hypothetical conserved protein	
**CcrKarma_gp326**	+	taagaatgAGGAaatacatcc	atg	agga	9	hypothetical conserved protein	
**CcrKarma_gp327**	+	aaggcccctcgGGAGccaccc	atg	ggag	6	hypothetical conserved protein	
**CcrKarma_gp328**	+	ccatcgcctGAGaccctcccc	atg	gag	9	hypothetical conserved protein	
**CcrKarma_gp329**	+	cgccgacGGGGtcgacctgac	gtg	gggg	10	putative RNaseH-like domain protein	IPR012337
**CcrKarma_gp330**	+	cgacccggcGGAGtttccggc	ttg	ggag	8	hypothetical conserved protein	
**CcrKarma_gp331**	+	acgaagccGAGGacGAGGacg	atg	gagg	3	hypothetical conserved protein	
**CcrKarma_gp332**	+	aaacgcaccGAGctttagatc	atg	gag	9	Terminase small subunit	HHpred vs pdb70_18Aug12, hit 3zqp_A, prob 90.3% E=0.16; position relative to terminase large subunit, see text
**CcrKarma_gp333**	+	tcgcgctgaccctagaacgcc	gtg	None	0	Terminase large subunit	IPR004921, IPR007868, IPR006141, IPR003587, IPR006517, SSF51294, G3DSA:2.170.16.10

Table S3. Predicted proteins, gene starts and annotations of *C. crescentus* phage CcrMagneto. Upstream sequence is the 25 bp of DNA sequence upstream of the start codon, with the predicted Shine-Dalgarno motif capitalized. S-D is the Shine-Dalgarno motif associated with the gene start, and spacing is the interval (in bp) between the S-D and start codon. Evidence includes detected conserved domains or motifs, BlastP and HHpred hits, and experimental or other evidence as detailed in the text.

Protein name	Strand	Upstream Sequence	Start Codon	S-D	spacing	Predicted product	Evidence
**CcrMagneto_gp001**	+	agccGGGGaccctggcgaatc	atg	gggg	13	hypothetical conserved protein	
**CcrMagneto_gp002**	+	cgatttcaaAGGcttactgcg	atg	agg	9	hypothetical conserved protein	
**CcrMagneto_gp003**	+	ccagcccttgtGAGGcctcgg	atg	gagg	6	hypothetical conserved protein	
**CcrMagneto_gp004**	+	ccgggcaAGGAGcttcccgcc	atg	aggag	9	hypothetical conserved protein	
**CcrMagneto_gp005**	+	gcgccttcGGAGaaaaccgcc	atg	ggag	9	hypothetical conserved protein	
**CcrMagneto_gp006**	+	cttcaatgacGGGGcggccgg	gtg	gggg	7	hypothetical conserved protein	
**CcrMagneto_gp007**	+	ggccttcaAGGAGtccaaccc	gtg	aggag	8	hypothetical conserved protein	
**CcrMagneto_gp008**	+	cccgtcatgacctctcattcc	atg	None	0	hypothetical conserved protein	
**CcrMagneto_gp009**	+	gccggccaAGGAtcgcagact	atg	agga	9	hypothetical conserved protein	
**CcrMagneto_gp010**	+	ccctcgaaAGGAtcgccgacc	atg	agga	9	hypothetical conserved protein	
**CcrMagneto_gp011**	+	gacgacggGGAGGcgtgagcc	atg	ggagg	8	hypothetical conserved protein	
**CcrMagneto_gp012**	+	gccgcccaAGGAttggaccgc	atg	agga	9	hypothetical conserved protein	
**CcrMagneto_gp013**	+	ccctcgcaAGGAtcgcacccc	atg	agga	9	hypothetical conserved protein	
**CcrMagneto_gp014**	+	tcgcctaAGGAGGcccgcccg	atg	aggagg	8	hypothetical conserved protein	
**CcrMagneto_gp015**	+	caaccgaAGGccgcctaagcc	atg	agg	11	hypothetical conserved protein	
**CcrMagneto_gp016**	+	ccgcaAGGAGGcctaaagccc	atg	aggagg	10	hypothetical conserved protein	
**CcrMagneto_gp017**	+	cttccttcGGAGcttctgatc	atg	ggag	9	hypothetical conserved protein	
**CcrMagneto_gp018**	+	cccctttctGGAtcgcttccc	atg	gga	9	hypothetical conserved protein	
**CcrMagneto_gp019**	+	ggtcgcaAGGAGacacccgac	atg	aggag	9	hypothetical conserved protein	
**CcrMagneto_gp020**	+	cacgcaatcgAGGAGcccgtc	atg	aggag	6	hypothetical conserved protein	
**CcrMagneto_gp021**	+	acgccgtcccggcctgatccc	atg	None	0	hypothetical conserved protein	
**CcrMagneto_gp022**	+	cgcccttGGAGttcccggacg	atg	ggag	10	hypothetical conserved protein	
**CcrMagneto_gp023**	+	caacaaGAGGTaccccgcctg	atg	gaggt	10	putative lipoprotein	LipoP
**CcrMagneto_gp024**	+	cgaaaccccggcgacgcgctg	atg	None	0	hypothetical novel protein	
**CcrMagneto_gp025**	+	acttcaacccgtAGGcgtagc	atg	agg	6	hypothetical conserved protein	
**CcrMagneto_gp026**	+	caccagcaacGAGttccattg	atg	gag	8	hypothetical conserved protein	
**CcrMagneto_gp027**	+	catccccatcggaGAGGtctg	atg	gagg	4	hypothetical conserved protein	
**CcrMagneto_gp028**	+	ctacgagcgcgtGGAGGctcc	gtg	ggagg	4	hypothetical conserved protein	
**CcrMagneto_gp029**	+	ggtgccggcGGAGccgctccc	gtg	ggag	8	hypothetical conserved protein	
**CcrMagneto_gp030**	+	tcgcgggcaAGGAGGcgatcc	atg	aggagg	6	hypothetical conserved protein	
**CcrMagneto_gp031**	+	gccccgcaAGGAGcgtgtctt	ttg	aggag	8	hypothetical conserved protein	
**CcrMagneto_gp032**	+	agctgccgcctgaAGGAcaac	atg	agga	4	hypothetical conserved protein	
**CcrMagneto_gp033**	+	tcgccccgtaacgAGGAcccc	atg	agga	4	hypothetical conserved protein	
**CcrMagneto_gp034**	+	ccctgatcaccGAGGcctgac	atg	gagg	6	hypothetical conserved protein	
**CcrMagneto_gp035**	+	catgaagaAGGgctccaagcg	gtg	agg	10	hypothetical conserved protein	
**CcrMagneto_gp036**	+	tgGGAacttggtgaagctctg	atg	gga	16	hypothetical conserved protein	
**CcrMagneto_gp037**	+	cgaaactgtGAGGcccattcg	atg	gagg	8	hypothetical conserved protein	
**CcrMagneto_gp038**	-	gctacctcaAGGAGtagcacc	gtg	aggag	7	hypothetical conserved protein	
**CcrMagneto_gp039**	-	atggttaatgacacttaaggg	gtg	None	0	putative transglutaminase-like cysteine peptidase	IPR010319
**CcrMagneto_gp040**	+	gcgacctcgcAGGccatctat	ctg	agg	8	putative portal protein	HHpred vs pdb70_18Aug12, hit 2jes_A, prob 99.9% E=4.5E-23; positional evidence, see text
**CcrMagneto_gp041**	+	cccgaacccgAGGAccgcccg	atg	agga	7	hypothetical conserved protein	
**CcrMagneto_gp042**	+	aaccctagcttgtaGAGtaac	atg	gag	4	hypothetical conserved protein	
**CcrMagneto_gp043**	+	ccgtcccaagaAGGcgacctg	atg	agg	7	hypothetical conserved protein	
**CcrMagneto_gp044**	+	cctccagccttGAGGcgcctt	gtg	gagg	6	hypothetical conserved protein	
**CcrMagneto_gp045**	+	gttaaccacGAGGgcttttcc	atg	gagg	8	hypothetical conserved protein	
**CcrMagneto_gp046**	+	ggctcctttcccGAGGgctcc	atg	gagg	5	hypothetical conserved protein	
**CcrMagneto_gp047**	+	cgaccgcgtgcGGGGcacctg	atg	gggg	6	hypothetical conserved protein	
**CcrMagneto_gp048**	+	ttcgtggttGGAGGgcgtaca	atg	ggagg	7	hypothetical conserved protein	
**CcrMagneto_gp049**	+	gatcaccgcttcggtcaagac	gtg	None	0	hypothetical conserved protein	
**CcrMagneto_gp050**	+	caccatctacGAGagcacgtg	atg	gag	6	hypothetical conserved protein	
**CcrMagneto_gp051**	+	gtcgtgaaaccggcccagccc	atg	None	0	hypothetical conserved protein	
**CcrMagneto_gp052**	+	tccggtcgAGGAGGgcgacat	ttg	aggagg	7	hypothetical conserved protein	
**CcrMagneto_gp053**	+	gcttatctccGGAccccgccg	atg	gga	8	hypothetical conserved protein	
**CcrMagneto_gp054**	+	ttaaccatgacgggctccccg	atg	None	0	hypothetical conserved protein	
**CcrMagneto_gp055**	+	cctgatgagcGGGGtggacgc	gtg	gggg	7	putative HD-domain/PDEase-like protein	SSF109604
**CcrMagneto_gp056**	+	aacgatacGAGaacatccgct	atg	gag	10	hypothetical conserved protein	
**CcrMagneto_gp057**	+	gctatctGGAtcgctgagccg	atg	gga	11	hypothetical conserved protein	
**CcrMagneto_gp058**	+	cctgccgaagaAGGccgcctg	atg	agg	7	putative DHH phosphoesterase protein	SSF64182
**CcrMagneto_gp059**	+	caagcgccgggcGGGGacggc	gtg	gggg	5	hypothetical conserved protein	
**CcrMagneto_gp060**	+	tctgccaacAGGAGatcgccg	gtg	aggag	7	hypothetical conserved protein	
**CcrMagneto_gp061**	+	ttccagcacatctAGGAcccc	atg	agga	4	hypothetical conserved protein	
**CcrMagneto_gp062**	+	gattacGAGGcctatcaccac	gtg	gagg	11	hypothetical conserved protein	
**CcrMagneto_gp063**	+	ttcgctgcGAGGccatttctg	atg	gagg	9	hypothetical conserved protein	
**CcrMagneto_gp064**	+	aagccggcgcgcaacttccgg	atg	None	0	putative HNH endonuclease	IPR002711
**CcrMagneto_gp065**	+	ccgtgggaaGAGagaaactgt	atg	gag	7	hypothetical conserved protein	
**CcrMagneto_gp066**	+	aacaccaagAGGAGcaagcca	atg	aggag	7	putative major capsid protein	experimental evidence, see text
**CcrMagneto_gp067**	+	atctaagcAGGAaatccccag	atg	agga	9	putative minor capsid protein	experimental evidence, see text
**CcrMagneto_gp068**	+	ccccgaaacGGAaaatccccc	atg	gga	9	hypothetical conserved protein	
**CcrMagneto_gp069**	+	aagcccacggcggcctaagcc	atg	None	0	hypothetical conserved protein	
**CcrMagneto_gp070**	+	atcacccAGGTgaactaagcc	gtg	aggt	10	hypothetical conserved protein	
**CcrMagneto_gp071**	+	caactccgctacgcctaagcc	atg	None	0	hypothetical conserved protein	
**CcrMagneto_gp072**	+	ttcacgagctaaGGGGcggcc	atg	gggg	5	hypothetical conserved protein	
**CcrMagneto_gp073**	+	aacccccAGGAGccctgaacc	atg	aggag	9	hypothetical conserved protein	
**CcrMagneto_gp074**	+	gccgcctcaAGGgtcttcgcg	ttg	agg	9	putative lectin-like domain protein	IPR013320, IPR008985
**CcrMagneto_gp075**	+	gctgacctGGAGGcctaagac	atg	ggagg	8	hypothetical conserved protein	
**CcrMagneto_gp076**	+	cgtggcgatgaagttctaacg	atg	None	0	hypothetical conserved protein	
**CcrMagneto_gp077**	+	tctGGAGtttcgagcgccatc	atg	ggag	14	hypothetical conserved protein	
**CcrMagneto_gp078**	+	cgcaccgccAGGgctttccca	atg	agg	9	hypothetical conserved protein	
**CcrMagneto_gp079**	+	ctgcggttgcgcGAGGcttcc	atg	gagg	5	hypothetical conserved protein	
**CcrMagneto_gp080**	+	ggcgtccgttcGAGagccatc	atg	gag	5	hypothetical conserved protein	
**CcrMagneto_gp081**	+	agtcGGGGaccgagagccgtc	atg	gggg	13	hypothetical conserved protein	
**CcrMagneto_gp082**	+	tcctgggtGGAGttttcccta	atg	ggag	9	hypothetical conserved protein	
**CcrMagneto_gp083**	+	cgccatcccagagGGAGcgac	gtg	ggag	4	putative PhoH-like protein	IPR003714, SSF52540, G3DSA:3.40.50.300
**CcrMagneto_gp084**	+	gtgacaaagacgtagcgcacc	atg	None	0	hypothetical conserved protein	
**CcrMagneto_gp085**	+	caagaagatcattcgccacta	atg	None	0	hypothetical conserved protein	
**CcrMagneto_gp086**	+	cagcacgcggaagaAGGcccc	gtg	agg	4	hypothetical conserved protein	
**CcrMagneto_gp087**	+	ctcgattaaaGAGGTgttcgg	atg	gaggt	6	hypothetical conserved protein	
**CcrMagneto_gp088**	+	gttttggctgggaGAGGactg	atg	gagg	4	hypothetical conserved protein	
**CcrMagneto_gp089**	+	ccgtGGAttggtgacctgccc	atg	gga	14	putative major tail tube protein	experimental evidence, see text
**CcrMagneto_gp090**	+	cccgatacGGAatccccgaac	atg	gga	10	putative pre-tape measure chaperone protein	HHpred vs pdb70_18Aug12, hit 2ob9_A, prob 89.3% E=0.82; positional evidence, see text
**CcrMagneto_gp091**	+	cccgatacGGAatccccgaac	atg	gga	10	putative pre-tape measure chaperone protein, frameshifted version	positional evidence, see text; slippery sequence AAAAAAC
**CcrMagneto_gp092**	+	gcGGGGccgccgataggatca	atg	gggg	15	putative tail tape measure protein	IPR007921, IPR013491, IPR013423; experimental evidence, see text
**CcrMagneto_gp093**	+	tcctttcatggttaatgcccg	atg	None	0	hypothetical conserved protein	IPR011740
**CcrMagneto_gp094**	+	aaatccgtccccgaagacctg	atg	None	0	putative tail protein	IPR019228, IPR018964, IPR011928; experimental evidence, see text
**CcrMagneto_gp095**	+	gcccaacgctccGGAGcagga	atg	ggag	5	putative NlpC/P60 family cell wall peptidase	IPR000064, SSF54001, G3DSA:3.90.1720.10
**CcrMagneto_gp096**	+	ccccggcgttgAGGActaatc	atg	agga	6	putative tail protein	IPR007110; experimental evidence, see text
**CcrMagneto_gp097**	+	tgtcataaaagtcgtggaacg	atg	None	0	hypothetical conserved protein	
**CcrMagneto_gp098**	+	cctcaAGGcgtcatcctgca	atg	agg	13	putative tail protein	IPR000421, IPR008979, G3DSA:2.60.120.260; experimental evidence, see text
**CcrMagneto_gp099**	+	accctcaagGGAatccgatag	atg	gga	9	hypothetical conserved protein	
**CcrMagneto_gp100**	+	tcttcatgctcGGAGcctgac	atg	ggag	6	hypothetical conserved protein	

**CcrMagneto_gp101**	+	tcagcacttccGGAattccac	atg	gga	7	putative endolysin	IPR002196, SSF53955, G3DSA:1.10.530.40
**CcrMagneto_gp102**	+	gggcttcaAGGgctgctgatc	atg	agg	10	putative inner membrane spanin component	N-terminal TMD, outer membrane spanin component embedded in +1 frame, see text
**CcrMagneto_gp103**	+	agtaccaaGAGGTtattcacg	atg	gaggt	8	putative outer membrane spanin component	lipoprotein embedded in inner membrane spanin component, see text
**CcrMagneto_gp104**	+	caagctcaaGGGGaagacgca	gtg	gggg	8	putative holin protein	predicted 4 TMDs, see text
**CcrMagneto_gp105**	+	cgctatcacGGAGtagggacc	atg	ggag	8	hypothetical conserved protein	
**CcrMagneto_gp106**	-	cgacggacggAGGAGaacgaa	atg	aggag	6	putative HTH domain DNA-binding protein	IPR001387, IPR010982, G3DSA:1.10.260.40
**CcrMagneto_gp107**	-	ttaaccaGAGGcccgaaaata	atg	gagg	10	putative dUTP pyrophosphatase	IPR008181, IPR008180, SSF51283, PTHR11241, G3DSA:2.70.40.10
**CcrMagneto_gp108**	-	tccttttcctGAGaccccttc	atg	gag	8	putative ribonucleoside diphosphate reductase beta subunit	IPR012348, IPR012335, IPR000358, IPR002109, IPR009078, IPR012336, IPR011767
**CcrMagneto_gp109**	-	tcgacgcGAGaacGAGacccc	atg	gag	5	putative ribonucleoside diphosphate reductase alpha subunit	IPR006141, IPR003587, IPR000788, IPR013509, IPR008926, SSF51294, SSF51998, G3DSA:2.170.16.10, G3DSA:3.20.70.20
**CcrMagneto_gp110**	-	cggtggttGGGGtggctggcg	ttg	gggg	9	hypothetical conserved protein	
**CcrMagneto_gp111**	-	tcctcatgcagcAGGAatccc	atg	agga	5	hypothetical conserved protein	
**CcrMagneto_gp112**	-	acgacatcaccGGAGccgagc	atg	ggag	6	hypothetical conserved protein	
**CcrMagneto_gp113**	-	gacgccgaccAGGActgatcc	atg	agga	7	putative thymidylate synthase	IPR003669
**CcrMagneto_gp114**	-	atccttcgctAGGAGaacccc	atg	aggag	6	putative dNMP kinase	HHpred vs pdb70_18Aug12, hit 2grj_A, prob 99.9% E=3.6E-27; SSF52540, G3DSA:3.40.50.300
**CcrMagneto_gp115**	-	cgtctcgtccgaaagtctccg	atg	None	0	putative RecD-like helicase	HHpred vs pdb70_18Aug12, hit 3e1s_A, prob 100% E=1.3E-45; SSF52540, G3DSA:3.40.50.300
**CcrMagneto_gp116**	-	acagaaaatAGGTatcagcaa	atg	aggt	8	hypothetical conserved protein	
**CcrMagneto_gp117**	-	aGAGaaatgtgacggtgcgcg	atg	gag	17	hypothetical conserved protein	
**CcrMagneto_gp118**	-	caagatgcgGGAGaaggccgc	gtg	ggag	8	hypothetical conserved protein	
**CcrMagneto_gp119**	-	actcccgaacGAGatcgcccg	atg	gag	8	putative exoribonuclease, Pol III-like	IPR006055, IPR013520, IPR012337, G3DSA:3.30.420.10
**CcrMagneto_gp120**	-	cacctgggcGGAGacgcactg	atg	ggag	8	hypothetical conserved protein	
**CcrMagneto_gp121**	-	atcaaatcctcaAGGTgctcc	atg	aggt	5	putative Pol I DNA polymerase, T7-like	IPR001098, IPR012337, SSF56672, PTHR10133, G3DSA:1.10.150.20, G3DSA:1.20.1060.10, G3DSA:3.30.420.10, G3DSA:3.30.70.370; see text
**CcrMagneto_gp122**	-	acatccgttaaccatagagcc	atg	None	0	putative DNA cytosine methyltransferase	IPR001525, IPR018117, SSF53335, PTHR10629:SF11, G3DSA:3.40.50.150, G3DSA:3.90.120.10
**CcrMagneto_gp123**	-	ggcgcctgtaagcGAGGtccc	atg	gagg	4	hypothetical conserved protein	
**CcrMagneto_gp124**	-	gcgccaaaccGGAGcttctct	atg	ggag	7	putative T5 A1-like protein	blastP to T5 A1 (AAU05157), E=3.51E-89, 34% Dice identity; HHpred vs pdb70_18Aug12, hit 1g2h_A, prob 94.0% E=0.05
**CcrMagneto_gp125**	-	gctggtcgcaaagacccctcc	atg	None	0	putative DNA methylase	IPR002052, SSF53335, G3DSA:3.40.50.150
**CcrMagneto_gp126**	-	cAGGAcctatgacgggttgcc	ctg	agga	16	hypothetical conserved protein	
**CcrMagneto_gp127**	-	aaagatcgctatcgttgtcgc	atg	None	0	hypothetical conserved protein	
**CcrMagneto_gp128**	-	tatcgcaaAGGTcatggacac	gtg	aggt	9	hypothetical conserved protein	
**CcrMagneto_gp129**	-	cgttaaccacgtgcggcgccc	atg	None	0	putative DNA helicase	IPR014001, IPR001650, IPR000330, IPR014021, SSF52540, PTHR10799, G3DSA:3.40.50.300
**CcrMagneto_gp130**	+	ccggtcacaAGGAGaactatc	atg	aggag	7	hypothetical conserved protein	
**CcrMagneto_gp131**	-	ggaaatcaAGGAGGccgcgtg	atg	aggagg	7	hypothetical conserved protein	
**CcrMagneto_gp132**	-	ccccgccgtaaAGGccccctg	atg	agg	7	hypothetical conserved protein	
**CcrMagneto_gp133**	-	acccacGAGGTctcgcgttac	ctg	gaggt	10	hypothetical conserved protein	
**CcrMagneto_gp134**	-	cgccttttcaAGGAtcgcccg	atg	agga	7	hypothetical conserved protein	
**CcrMagneto_gp135**	-	gccggtctcaAGGAGacgacc	atg	aggag	6	Putative rIIb-like protein	PD130832
**CcrMagneto_gp136**	-	gcctgcctttcGGAGtttgcc	atg	ggag	6	putative rIIa-like protein	IPR003594
**CcrMagneto_gp137**	+	ggcaaactccgaaAGGcAGGc	ttg	agg	1	hypothetical conserved protein	
**CcrMagneto_gp138**	+	gcctGAGGcatgctcgtattc	atg	gagg	13	hypothetical conserved protein	
**CcrMagneto_gp139**	+	cgtatggtacgcgAGGAtgac	atg	agga	4	hypothetical conserved protein	
**CcrMagneto_gp140**	+	caccgacgggcgcgtcctcca	ttg	None	0	hypothetical conserved protein	
**CcrMagneto_gp141**	+	acgacaacgaAGGAGGctctc	atg	aggagg	5	putative tyrosine recombinase	IPR013762, IPR002104, IPR011010
**CcrMagneto_gp142**	+	gtcctgcgatAGGAGGgcgcg	atg	aggagg	5	hypothetical conserved protein	
**CcrMagneto_gp143**	+	cgctagtcccAGGAGtacagt	ttg	aggag	6	hypothetical conserved protein	
**CcrMagneto_gp144**	+	acccccgtcccgacAGGcttc	atg	agg	4	hypothetical conserved protein	
**CcrMagneto_gp145**	+	atcgctcctGGGGccttttga	gtg	gggg	8	hypothetical conserved protein	
**CcrMagneto_gp146**	+	tcctgtacggtaagGGAaaac	atg	gga	4	hypothetical conserved protein	
**CcrMagneto_gp147**	+	acgattcGGAGtattcatgaa	atg	ggag	10	putative HTH domain protein	IPR001387, G3DSA:1.10.260.40
**CcrMagneto_gp148**	+	cctgtattcctGAGGTcccgc	atg	gaggt	5	putative tRNA amidotransferase domain protein	IPR019004, IPR003789
**CcrMagneto_gp149**	-	actgatcgaGGGGaagttgag	atg	gggg	8	putative DNA ligase	IPR012340, IPR016027, SSF56091, G3DSA:3.30.470.30
**CcrMagneto_gp150**	-	tcgatccGGAGattttcggcg	atg	ggag	10	hypothetical conserved protein	
**CcrMagneto_gp151**	-	gccatcccctctatctccgag	gtg	None	0	hypothetical conserved protein	
**CcrMagneto_gp152**	-	gtttcAGGgcaaatgtcAGGa	atg	agg	1	DUF1643 protein	IPR012441
**CcrMagneto_gp153**	-	gcctcgcccttgaAGGAggca	atg	agga	4	hypothetical conserved protein	
**CcrMagneto_gp154**	-	gAGGccaagaagcgggccacg	gtg	agg	17	hypothetical conserved protein	
**CcrMagneto_gp155**	-	ctGGAtcgccccggcggcctg	atg	gga	16	hypothetical conserved protein	
**CcrMagneto_gp156**	-	gcttttgAGGAGtttggacgt	atg	aggag	9	hypothetical conserved protein	
**CcrMagneto_gp157**	-	agcaggactAGGAGaaagacc	atg	aggag	7	hypothetical conserved protein	
**CcrMagneto_gp158**	-	cgacgccaAGGAcgtctgcca	atg	agga	9	hypothetical conserved protein	
**CcrMagneto_gp159**	-	ggccggctacGAGatcgcctg	atg	gag	8	putative lipoprotein	LipoP
**CcrMagneto_gp160**	-	cgcttaaaGGGGgacctcatc	atg	gggg	8	putative nicotinate phosphoribosyltransferase	IPR015977, IPR002638, PTHR11098
**CcrMagneto_gp161**	-	cagaatgaGGGGtaagcgata	gtg	gggg	9	putative NUDIX hydrolase domain protein	IPR014729, IPR000086, IPR015797, SSF52374, PS51462, PTHR22769
**CcrMagneto_gp162**	-	caaagcgtagtgtcgcgaggc	atg	None	0	hypothetical conserved protein	
**CcrMagneto_gp163**	-	ctcctacgaaagccgcttttc	atg	None	0	putative peptidyl-tRNA hydrolase	IPR002833, SSF102462, PTHR12649, G3DSA:3.40.1490.10
**CcrMagneto_gp164**	-	agcgccggccgccGAGGgctg	atg	gagg	4	hypothetical conserved protein	
**CcrMagneto_gp165**	-	taacctccagcAGGAGaccgc	atg	aggag	5	hypothetical conserved protein	
**CcrMagneto_gp166**	-	acaacgccGGAGGccctttc	atg	ggagg	8	hypothetical conserved protein	
**CcrMagneto_gp167**	-	ccccgatctccGAGaagcccc	atg	gag	7	putative HD-domain/PDEase-like protein	SSF109604
**CcrMagneto_gp168**	-	gggagaattcctGAGGgtcgc	atg	gagg	5	hypothetical conserved protein	
**CcrMagneto_gp169**	-	acgcccagaAGGActaatcct	atg	agga	8	putative DNA-binding domain protein	IPR016177, SSF54060
**CcrMagneto_gp170**	-	gaggcctcGGAGaagtaagcc	atg	ggag	9	hypothetical conserved protein	
**CcrMagneto_gp171**	-	tcttgagaAGGAacaaccctg	atg	agga	9	hypothetical conserved protein	
**CcrMagneto_gp172**	-	tcaacaAGGgcaagaacgccg	atg	agg	12	hypothetical conserved protein	
**CcrMagneto_gp173**	-	caagcAGGcggcgggtctgct	ttg	agg	13	hypothetical conserved protein	
**CcrMagneto_gp174**	-	gctgttcgactGGAGaaaatc	gtg	ggag	6	hypothetical conserved protein	
**CcrMagneto_gp175**	-	ctctgcgtgaAGGgctAGGcc	atg	agg	2	hypothetical conserved protein	
**CcrMagneto_gp176**	-	acaccacagaAGGAGcccgcc	gtg	aggag	6	putative RtcB-like protein	IPR001233
**CcrMagneto_gp177**	-	gtacccctcaAGGTgatcccc	atg	aggt	7	hypothetical conserved protein	
**CcrMagneto_gp178**	-	aacaaagcaAGGAGcaacccc	ttg	aggag	7	putative band 7 lipoprotein	IPR001107
**CcrMagneto_gp179**	-	actcctgaaAGGccgcgccca	atg	agg	9	hypothetical conserved protein	
**CcrMagneto_gp180**	-	gcagcgcgggctcgcggcctg	atg	None	0	hypothetical conserved protein	
**CcrMagneto_gp181**	-	gacgacaacgaAGGAGactcc	atg	aggag	5	hypothetical conserved protein	
**CcrMagneto_gp182**	-	caggatGGAGGccgccgccaa	atg	ggagg	10	hypothetical conserved protein	
**CcrMagneto_gp183**	-	tccgagccaAGGAGcgctgat	ttg	aggag	7	hypothetical conserved protein	
**CcrMagneto_gp184**	-	cgacagcgagAGGAGccgatc	atg	aggag	6	hypothetical conserved protein	
**CcrMagneto_gp185**	-	gcactctGGAaagGGAccgcc	atg	gga	5	hypothetical conserved protein	
**CcrMagneto_gp186**	-	ggtcctctcgGGAGacaaccc	atg	ggag	7	hypothetical conserved protein	
**CcrMagneto_gp187**	-	tctgaaAGGctcgacctcctg	atg	agg	12	hypothetical conserved protein	
**CcrMagneto_gp188**	-	cgttccaactcgcAGGTctcc	gtg	aggt	4	hypothetical conserved protein	
**CcrMagneto_gp189**	-	cctgggcctGGAGaagcacct	gtg	ggag	8	hypothetical conserved protein	
**CcrMagneto_gp190**	-	actggcgaAGGTgtcggacca	atg	aggt	9	hypothetical conserved protein	
**CcrMagneto_gp191**	-	acaaccgcagAGGAGaccctc	gtg	aggag	6	hypothetical conserved protein	
**CcrMagneto_gp192**	-	cgGGAtgcgttccgggtcgag	atg	gga	16	hypothetical conserved protein	
**CcrMagneto_gp193**	-	gcccgagaaAGGcgaAGGccc	atg	agg	3	hypothetical conserved protein	
**CcrMagneto_gp194**	-	cgccgacGGAGaattactgcg	atg	ggag	10	putative winged-helix HTH DNA binding protein	IPR011991
**CcrMagneto_gp195**	-	ttacgcgccaatGAGcgccag	atg	gag	6	hypothetical conserved protein	
**CcrMagneto_gp196**	-	ccacccgcgctgcccgaggag	gtg	None	0	hypothetical conserved protein	
**CcrMagneto_gp197**	-	cagcgcgggcAGGgttcccgc	atg	agg	8	hypothetical conserved protein	
**CcrMagneto_gp198**	-	aagaggtcgAGGAGGTcgccg	atg	aggaggt	5	hypothetical conserved protein	
**CcrMagneto_gp199**	-	gacgacaacgAGGAGGTcctg	atg	aggaggt	4	putative ArdC-like antirestriction protein	IPR017113, IPR013610
**CcrMagneto_gp200**	-	cgcggcgataatGAGcgcctg	atg	gag	6	hypothetical conserved protein	
**CcrMagneto_gp201**	-	ggacctGGAGaacaaccgccg	atg	ggag	11	hypothetical conserved protein	
**CcrMagneto_gp202**	-	gggccggGGAGGgcgtgcatc	atg	ggagg	9	hypothetical conserved protein	
**CcrMagneto_gp203**	-	cgcctgccaGAGcctcaagtc	atg	gag	9	hypothetical conserved protein	
**CcrMagneto_gp204**	-	aacgacaccagggGGAGagcc	gtg	ggag	4	putative acyl carrier protein	IPR009081, IPR006163, PTHR20863

**CcrMagneto_gp205**	-	tcgacaacggcaAGGAcggct	atg	agga	5	putative HNH endonuclease	IPR002711
**CcrMagneto_gp206**	-	ctcattcgttaAGGAtcgacc	atg	agga	6	hypothetical conserved protein	
**CcrMagneto_gp207**	-	cgttcctgggctatcaagccg	atg	None	0	hypothetical conserved protein	
**CcrMagneto_gp208**	-	tggGGAGaagctgaagtgagc	atg	ggag	14	hypothetical conserved protein	
**CcrMagneto_gp209**	-	cgccttcGAGGTgcaagcctg	atg	gaggt	9	hypothetical conserved protein	
**CcrMagneto_gp210**	-	tccggacctcGGAGaaggccc	gtg	ggag	7	hypothetical conserved protein	
**CcrMagneto_gp211**	-	acggttcgAGGAGatcgaggc	ttg	aggag	8	hypothetical conserved protein	
**CcrMagneto_gp212**	-	gacgacaacggaAGGAacctt	atg	agga	5	hypothetical conserved protein	
**CcrMagneto_gp213**	-	cggtgacttcgccaAGGggac	ctg	agg	4	hypothetical conserved protein	
**CcrMagneto_gp214**	-	gcgcgaaccgaAGGTcgactg	atg	aggt	6	hypothetical conserved protein	
**CcrMagneto_gp215**	-	gacgacaacaAGGAGGcctcc	atg	aggagg	5	hypothetical conserved protein	
**CcrMagneto_gp216**	-	ctacgtGGAGagccgggcggc	atg	ggag	11	hypothetical conserved protein	
**CcrMagneto_gp217**	-	cgcgcaatttccGGAGtgacc	atg	ggag	5	hypothetical conserved protein	SSF56784
**CcrMagneto_gp218**	-	gtcctgGGAGttcgcggactg	atg	ggag	11	hypothetical conserved protein	
**CcrMagneto_gp219**	-	gcagggcctGGGGgactgaac	atg	gggg	7	hypothetical novel protein	
**CcrMagneto_gp220**	-	gatcaagtcGAGGccctgagc	atg	gagg	8	hypothetical conserved protein	
**CcrMagneto_gp221**	-	ggctcccgtacAGGAtcacct	atg	agga	6	hypothetical conserved protein	
**CcrMagneto_gp222**	-	caccgggctcgtggcggcctg	atg	None	0	hypothetical conserved protein	
**CcrMagneto_gp223**	-	agcagaAGGAGGaagccgctc	atg	aggagg	9	putative HTH domain DNA-binding protein	IPR010982, IPR001387, G3DSA:1.10.260.40
**CcrMagneto_gp224**	-	aatcgcccAGGgcgccgcctg	atg	agg	10	hypothetical conserved protein	
**CcrMagneto_gp225**	-	acaacgacagaAGGAGGccgc	gtg	aggagg	4	hypothetical conserved protein	
**CcrMagneto_gp226**	-	ggccggcgcggcggcgtccgc	atg	None	0	hypothetical conserved protein	
**CcrMagneto_gp227**	-	cactgtccggaAGGTcgagcc	atg	aggt	6	hypothetical conserved protein	
**CcrMagneto_gp228**	-	ggtctatcgccGGAGGgcctg	atg	ggagg	5	putative Pol I DNA polymerase	IPR001098, IPR002298, SSF56672, G3DSA:3.30.70.370
**CcrMagneto_gp229**	-	agatcaAGGAGtagtgcaagc	gtg	aggag	10	putative GcrA-like cell cycle regulator	IPR011681; see text
**CcrMagneto_gp230**	-	gccgccaaattcGAGGggaag	atg	gagg	5	hypothetical conserved protein	
**CcrMagneto_gp231**	+	cgacaacgaaAGGAGcctgcc	atg	aggag	6	hypothetical conserved protein	
**CcrMagneto_gp232**	+	cgccgcGAGGctcgcccaatg	atg	gagg	11	hypothetical conserved protein	
**CcrMagneto_gp233**	+	caagatcGAGGaaGAGGtctg	atg	gagg	4	hypothetical conserved protein	
**CcrMagneto_gp234**	+	caagctcaagaaAGGTcgcct	atg	aggt	5	hypothetical conserved protein	
**CcrMagneto_gp235**	+	cattctcgactAGGAGGaccc	atg	aggagg	4	hypothetical conserved protein	
**CcrMagneto_gp236**	+	tcgatttcctggcgggcgccg	atg	None	0	hypothetical conserved protein	
**CcrMagneto_gp237**	+	gtttctggaAGGTcaggactg	atg	aggt	8	hypothetical conserved protein	
**CcrMagneto_gp238**	+	agcgcgagccgcAGGTctgac	atg	aggt	5	hypothetical conserved protein	
**CcrMagneto_gp239**	+	cgacagcgaaAGGAGcctgcc	ttg	aggag	6	hypothetical conserved protein	
**CcrMagneto_gp240**	+	ccggatgctGGAGGTcgcggc	ttg	ggaggt	6	hypothetical conserved protein	
**CcrMagneto_gp241**	+	actcatggacGGAGccgtctg	atg	ggag	7	hypothetical conserved protein	
**CcrMagneto_gp242**	+	cgccgacttcaAGGAcaagcg	atg	agga	6	hypothetical conserved protein	
**CcrMagneto_gp243**	+	gccgccGGAGGcagacccgac	gtg	ggagg	10	hypothetical conserved protein	
**CcrMagneto_gp244**	+	tcaagctGGAGaccgtctgac	atg	ggag	10	hypothetical conserved protein	
**CcrMagneto_gp245**	+	acgacaacgagAGGAGttccc	atg	aggag	5	hypothetical conserved protein	
**CcrMagneto_gp246**	+	cacggaatgGGAGattttcga	atg	ggag	8	hypothetical conserved protein	
**CcrMagneto_gp247**	+	ggcctGGAGatcatcgcctag	atg	ggag	12	hypothetical conserved protein	
**CcrMagneto_gp248**	+	acctcggacgAGGAGGactaa	atg	aggagg	5	hypothetical conserved protein	
**CcrMagneto_gp249**	+	gaccccGGAGtccaagaccga	atg	ggag	11	hypothetical conserved protein	
**CcrMagneto_gp250**	+	caacgccctgGGAGccgccaa	atg	ggag	7	hypothetical conserved protein	
**CcrMagneto_gp251**	+	ggtcagccAGGAcatgccgtc	atg	agga	9	hypothetical conserved protein	
**CcrMagneto_gp252**	+	gacgacaacgAGGAGGcttcg	atg	aggagg	5	hypothetical conserved protein	
**CcrMagneto_gp253**	+	gtcaaagAGGAGGcctgagcc	ttg	aggagg	8	hypothetical conserved protein	
**CcrMagneto_gp254**	+	ggcgccctacGAGGccccgca	atg	gagg	7	hypothetical conserved protein	
**CcrMagneto_gp255**	+	ccggcgAGGAGGTgcgctgac	atg	aggaggt	8	hypothetical conserved protein	
**CcrMagneto_gp256**	+	tggacgacaacAGGAGGgacc	atg	aggagg	4	hypothetical conserved protein	
**CcrMagneto_gp257**	+	ctacgtgaAGGAcctgcgtcg	atg	agga	9	hypothetical conserved protein	
**CcrMagneto_gp258**	+	tcagcgtGGGGgtggctgctg	atg	gggg	9	hypothetical conserved protein	
**CcrMagneto_gp259**	+	gacccaGGGGgtttccgaggc	atg	gggg	10	hypothetical conserved protein	
**CcrMagneto_gp260**	+	cctcgacgcgAGGAaaagctg	atg	agga	7	hypothetical conserved protein	
**CcrMagneto_gp261**	+	aagccctgaagGGAGagaccg	atg	ggag	6	hypothetical conserved protein	
**CcrMagneto_gp262**	+	gaccaaGGGGgtgcGGGGcaa	atg	gggg	3	hypothetical conserved protein	
**CcrMagneto_gp263**	+	ttgtcttGAGaaagccaacgc	atg	gag	11	DUF1937 protein	IPR015235, SSF52309
**CcrMagneto_gp264**	+	gagcaccagGGAGattgaagc	gtg	ggag	8	hypothetical conserved protein	
**CcrMagneto_gp265**	+	ggtgtcccaGAGGTaccagcg	atg	gaggt	7	hypothetical conserved protein	
**CcrMagneto_gp266**	+	ccggcgcctatGGAGagagcc	atg	ggag	6	putative TAT signal protein	IPR006311; PRED-TAT
**CcrMagneto_gp267**	+	tctcgatatggcgaagtccgc	atg	None	0	hypothetical conserved protein	
**CcrMagneto_gp268**	+	acgtcaacaAGGAGcccaccg	atg	aggag	7	hypothetical conserved protein	
**CcrMagneto_gp269**	+	agaagcccggcgggccgagtg	atg	None	0	hypothetical conserved protein	
**CcrMagneto_gp270**	+	ccaaactcgaccggttctgac	atg	None	0	hypothetical conserved protein	
**CcrMagneto_gp271**	+	gccgctcgatgggtGGAacgg	atg	gga	4	hypothetical conserved protein	
**CcrMagneto_gp272**	+	cgaggaagAGGAGcgctgctg	atg	aggag	8	putative Ser/Thr protein phosphatase	IPR004843, IPR006186, SSF56300, PTHR11668, G3DSA:3.60.21.10
**CcrMagneto_gp273**	+	gttgatccgcgAGGAcgacgc	atg	agga	6	hypothetical conserved protein	
**CcrMagneto_gp274**	+	gacgacaacgAGGAGGcccac	atg	aggagg	5	hypothetical conserved protein	
**CcrMagneto_gp275**	+	catgaccaaGAGccgcttctg	atg	gag	9	hypothetical conserved protein	
**CcrMagneto_gp276**	+	gatcatcctgGGAGatgccgc	atg	ggag	7	hypothetical conserved protein	
**CcrMagneto_gp277**	+	ccgcttcgAGGAGGcccgccc	atg	aggagg	7	hypothetical conserved protein	
**CcrMagneto_gp278**	+	acgacaacgAGGAGGcccccc	atg	aggagg	6	hypothetical conserved protein	
**CcrMagneto_gp279**	+	cttcgtctcgggtgaagcctg	atg	None	0	hypothetical conserved protein	
**CcrMagneto_gp280**	+	ccacGGAagaagacgcctaag	atg	gga	14	hypothetical conserved protein	
**CcrMagneto_gp281**	+	cGGAGGgctaacgtgagtctg	atg	ggagg	15	hypothetical conserved protein	
**CcrMagneto_gp282**	+	tccgaaaGGGGccttaagtcc	atg	gggg	10	hypothetical conserved protein	
**CcrMagneto_gp283**	+	aacggaAGGAcctgaaagacc	atg	agga	11	hypothetical conserved protein	
**CcrMagneto_gp284**	+	acaacgccGGAGGccctttttc	atg	ggagg	8	hypothetical conserved protein	
**CcrMagneto_gp285**	+	atactacgccaagcAGGccgc	atg	agg	4	hypothetical conserved protein	
**CcrMagneto_gp286**	+	caacatcgaaGAGttcggcga	atg	gag	8	hypothetical conserved protein	
**CcrMagneto_gp287**	+	ccagatcaAGGAGGcccagtc	ttg	aggagg	7	hypothetical conserved protein	
**CcrMagneto_gp288**	+	caatcagcaataGGGGccagc	atg	gggg	5	hypothetical conserved protein	
**CcrMagneto_gp289**	+	ctgggccGAGGgctcgccgcg	atg	gagg	10	hypothetical conserved protein	
**CcrMagneto_gp290**	+	cggacatagAGGAGcaacccg	atg	aggag	7	hypothetical conserved protein	
**CcrMagneto_gp291**	+	tcgccgcctgAGGAGGacccg	atg	aggagg	5	hypothetical conserved protein	
**CcrMagneto_gp292**	+	gcttgagaAGGcgttcggcca	atg	agg	10	hypothetical conserved protein	
**CcrMagneto_gp293**	+	ccaagcctcGGGGctcttcca	atg	gggg	8	hypothetical conserved protein	
**CcrMagneto_gp294**	+	caccgactactAGGAGGtccc	atg	aggagg	4	hypothetical conserved protein	
**CcrMagneto_gp295**	+	ccgctaggacaAGGAGGtccc	atg	aggagg	4	hypothetical conserved protein	
**CcrMagneto_gp296**	+	acgacaacAGGAGGccccgcc	ttg	aggagg	7	hypothetical conserved protein	
**CcrMagneto_gp297**	+	ttgcggtcatgAGGAttcgaa	atg	agga	6	hypothetical conserved protein	
**CcrMagneto_gp298**	+	ccggcggctgacgggtgcgtc	atg	None	0	hypothetical conserved protein	
**CcrMagneto_gp299**	+	gacgacaacgAGGAGGccccc	atg	aggagg	5	hypothetical conserved protein	
**CcrMagneto_gp300**	+	ggactacatcGAGGTgaccct	gtg	gaggt	6	hypothetical conserved protein	
**CcrMagneto_gp301**	+	ccttagccaaGAGGTgcagcc	ttg	gaggt	6	hypothetical conserved protein	
**CcrMagneto_gp302**	+	agcggcGGAGGccctggcctg	atg	ggagg	10	hypothetical conserved protein	
**CcrMagneto_gp303**	+	ggaacAGGAGaccgccgcctg	atg	aggag	11	hypothetical conserved protein	
**CcrMagneto_gp304**	+	gatcgcgcgactGGAGaacgc	atg	ggag	5	hypothetical conserved protein	
**CcrMagneto_gp305**	+	cggcgggatcaAGGTgagcgc	atg	aggt	6	hypothetical conserved protein	
**CcrMagneto_gp306**	+	tttcatcgccGAGGccctgtc	atg	gagg	7	hypothetical conserved protein	
**CcrMagneto_gp307**	+	acgctgcgaAGGAGcctcgac	ttg	aggag	7	hypothetical conserved protein	
**CcrMagneto_gp308**	+	ccagatcAGGAaggccgccgc	atg	agga	10	hypothetical conserved protein	

**CcrMagneto_gp309**	+	tcgcgacacggcaAGGTacgc	atg	aggt	4	hypothetical conserved protein	
**CcrMagneto_gp310**	+	cggcctgcatgcGAGGggctg	atg	gagg	5	hypothetical conserved protein	
**CcrMagneto_gp311**	+	gatctacaagccggtgcgctg	atg	None	0	hypothetical conserved protein	
**CcrMagneto_gp312**	+	gctcgccaAGGAcgtggtgtg	atg	agga	9	hypothetical conserved protein	
**CcrMagneto_gp313**	+	ccgcctGGAGcttgccgccac	atg	ggag	11	hypothetical conserved protein	
**CcrMagneto_gp314**	+	cctcggctGGAagtcctcgtc	atg	gga	10	hypothetical conserved protein	
**CcrMagneto_gp315**	+	cctggagtatcGGGGtcgggc	gtg	gggg	6	putative HNH homing endonuclease	IPR002711
**CcrMagneto_gp316**	+	cgggctagaccGGAGGccatg	atg	ggagg	5	hypothetical conserved protein	
**CcrMagneto_gp317**	+	gatccttccGGAGGacgcggc	gtg	ggagg	7	hypothetical conserved protein	
**CcrMagneto_gp318**	+	caggctgaaGAGaccgggcgg	atg	gag	9	hypothetical conserved protein	
**CcrMagneto_gp319**	+	caccctcaacaAGGAGcccct	gtg	aggag	5	hypothetical conserved protein	
**CcrMagneto_gp320**	+	tccgcgcctaaAGGAcccccg	atg	agga	6	hypothetical novel protein	
**CcrMagneto_gp321**	+	taagaatgAGGAaatacatcc	atg	agga	9	hypothetical conserved protein	
**CcrMagneto_gp322**	+	aaggcccctcgGGAGccaccc	atg	ggag	6	hypothetical conserved protein	
**CcrMagneto_gp323**	+	gccatcgcctGAGaccctccc	atg	gag	8	hypothetical conserved protein	
**CcrMagneto_gp324**	+	cgccgacGGGGtcgacctgac	gtg	gggg	10	putative RNaseH-like domain protein	IPR012337
**CcrMagneto_gp325**	+	cgacccggcGGAGtttccggc	ttg	ggag	8	hypothetical conserved protein	
**CcrMagneto_gp326**	+	aagaagacGAGGacGAGGacg	atg	gagg	3	hypothetical conserved protein	
**CcrMagneto_gp327**	+	aaacgcaccGAGctttagatc	atg	gag	9	putative terminase small subunit	HHpred vs pdb70_18Aug12, hit 3zqp_A, prob 90.4% E=0.15; position relative to terminase large subunit, see text
**CcrMagneto_gp328**	+	tcgcgctgaccctagaacgcc	gtg	None	0	putative terminase large subunit	IPR004921, IPR007868, IPR006141, IPR003587, IPR006517, SSF51294, G3DSA:2.170.16.10

Table S4. Predicted proteins, gene starts and annotations of *C. crescentus* phage CcrSwift. Upstream sequence is the 25 bp of DNA sequence upstream of the start codon, with the predicted Shine-Dalgarno motif capitalized. S-D is the Shine-Dalgarno motif associated with the gene start, and spacing is the interval (in bp) between the S-D and start codon. Evidence includes detected conserved domains or motifs, BlastP and HHpred hits, and experimental or other evidence as detailed in the text.

Protein name	Strand	Upstream Sequence	Start Codon	S-D	spacing	Predicted product	Evidence
**CcrSwift_gp001**	+	tggcgaatcatGGAGccgacg	atg	ggag	6	hypothetical conserved protein	
**CcrSwift_gp002**	+	cgaaatcaaAGGcttatcgcc	atg	agg	9	hypothetical conserved protein	
**CcrSwift_gp003**	+	atcccgttaccGGAcaacccc	atg	gga	7	hypothetical conserved protein	
**CcrSwift_gp004**	-	cgcgcccttGGAGatcgcccc	atg	ggag	8	hypothetical conserved protein	
**CcrSwift_gp005**	+	tttgacattgtGAGGcaatcc	atg	gagg	6	hypothetical conserved protein	
**CcrSwift_gp006**	+	aacccctGGAGGccctgcccc	atg	ggagg	9	hypothetical conserved protein	
**CcrSwift_gp007**	+	cgacgccgccaAGGTgaaggc	gtg	aggt	6	hypothetical conserved protein	
**CcrSwift_gp008**	+	gcgtttactttttgttaagga	atg	None	0	hypothetical conserved protein	
**CcrSwift_gp009**	+	gccccgctgtcgaaagccgcc	atg	None	0	hypothetical conserved protein	
**CcrSwift_gp010**	+	cctcacctGGAGattttcccc	atg	ggag	9	hypothetical conserved protein	
**CcrSwift_gp011**	+	ccgcactGGAaagccaagccc	atg	gga	11	hypothetical conserved protein	
**CcrSwift_gp012**	+	cccccgcaAGGAtcgcccgac	atg	agga	9	hypothetical conserved protein	
**CcrSwift_gp013**	+	gGAGccgcaaccatgcccccg	atg	gag	17	hypothetical conserved protein	
**CcrSwift_gp014**	+	ctgccgaAGGTcgcctaagcc	atg	aggt	10	hypothetical conserved protein	
**CcrSwift_gp015**	+	caagccaAGGAagcctagacc	atg	agga	10	hypothetical conserved protein	
**CcrSwift_gp016**	+	cttccttcGGAGcttctgatc	atg	ggag	9	hypothetical conserved protein	
**CcrSwift_gp017**	+	cccctttctGGAtcgctcccc	atg	gga	9	hypothetical conserved protein	
**CcrSwift_gp018**	+	ggtcgcaAGGAGacacccgac	atg	aggag	9	hypothetical conserved protein	
**CcrSwift_gp019**	+	cacgcaatcgAGGAGcccgtc	atg	aggag	6	hypothetical conserved protein	
**CcrSwift_gp020**	+	tccgAGGAcggcaagcacgtc	atg	agga	13	hypothetical conserved protein	
**CcrSwift_gp021**	+	cgcccttGGAGttcccggacg	atg	ggag	10	hypothetical conserved protein	
**CcrSwift_gp022**	+	caacaaGAGGTaccccgcctg	atg	gaggt	10	putative lipoprotein	LipoP
**CcrSwift_gp023**	+	acttcaacccgtAGGcgtatc	atg	agg	6	hypothetical conserved protein	
**CcrSwift_gp024**	+	caccagcaacGAGttccattg	atg	gag	8	hypothetical conserved protein	
**CcrSwift_gp025**	+	catccccatcggaGAGGcctg	atg	gagg	4	hypothetical conserved protein	
**CcrSwift_gp026**	+	ctacgagcgcgtGGAGGctcc	gtg	ggagg	4	hypothetical conserved protein	
**CcrSwift_gp027**	+	ggtcccgctGGAGcccctgcc	gtg	ggag	8	hypothetical conserved protein	
**CcrSwift_gp028**	+	ttgccggcaAGGAGGTggtcc	atg	aggaggt	5	hypothetical conserved protein	
**CcrSwift_gp029**	+	gccccgcaAGGAGcgtgtctt	ttg	aggag	8	hypothetical conserved protein	
**CcrSwift_gp030**	+	agacgccgcctgaAGGAcaac	atg	agga	4	hypothetical conserved protein	
**CcrSwift_gp031**	+	tcgccccgtaacgAGGAcccc	atg	agga	4	hypothetical conserved protein	
**CcrSwift_gp032**	+	ccctgatcaccGAGGcctgac	atg	gagg	6	hypothetical conserved protein	
**CcrSwift_gp033**	+	gggcatgaagaAGGcgaaacg	atg	agg	7	hypothetical conserved protein	
**CcrSwift_gp034**	+	gcgcGGAcaaccggcggcggc	atg	gga	14	hypothetical conserved protein	
**CcrSwift_gp035**	+	gacatcaAGGAcgccttcgcc	ctg	agga	10	hypothetical conserved protein	
**CcrSwift_gp036**	+	tGAGGTcttgaggaccattcg	atg	gaggt	15	hypothetical novel protein	
**CcrSwift_gp037**	+	acctgggggcGGAGGTgtatg	atg	ggaggt	5	hypothetical conserved protein	
**CcrSwift_gp038**	-	gctacctcaAGGAGtagcgac	gtg	aggag	7	hypothetical conserved protein	
**CcrSwift_gp039**	-	gacacttaaGGGGtgtcgatc	atg	gggg	8	putative transglutaminase-like cysteine peptidase	IPR010319
**CcrSwift_gp040**	+	gcgacctcgcAGGccatctat	ctg	agg	8	putative portal protein	HHpred vs pdb70_18Aug12, hit 2jes_A, prob 99.9% E=4.9E-23; positional evidence, see text
**CcrSwift_gp041**	+	cccgaacccgAGGAccgcccg	atg	agga	7	hypothetical conserved protein	
**CcrSwift_gp042**	+	aaccctagcttgtaGAGtaac	atg	gag	4	hypothetical conserved protein	
**CcrSwift_gp043**	+	ccgtcccaagaAGGcgacctg	atg	agg	7	hypothetical conserved protein	
**CcrSwift_gp044**	+	cctccagccttGAGGcgcctt	gtg	gagg	6	hypothetical conserved protein	
**CcrSwift_gp045**	+	gttaaccacGAGGgcttttcc	atg	gagg	8	hypothetical conserved protein	
**CcrSwift_gp046**	+	ggctcctttcccGAGGgctct	atg	gagg	5	hypothetical conserved protein	
**CcrSwift_gp047**	+	cgaccgcgtgcGGGGcacctg	atg	gggg	6	hypothetical conserved protein	
**CcrSwift_gp048**	+	tccaagcttAGGAGcccgtcc	atg	aggag	7	hypothetical conserved protein	
**CcrSwift_gp049**	+	gatcaccgcctcggtcaagac	gtg	None	0	hypothetical conserved protein	
**CcrSwift_gp050**	+	gaccatctacGAGagcacgtg	atg	gag	6	hypothetical conserved protein	
**CcrSwift_gp051**	+	gccgtgaaaccggcccagccc	atg	None	0	hypothetical conserved protein	
**CcrSwift_gp052**	+	tccggtcgAGGAGGgcgacat	ttg	aggagg	7	hypothetical conserved protein	
**CcrSwift_gp053**	+	gcgcgaagccgccaagcgtcc	gtg	None	0	hypothetical conserved protein	
**CcrSwift_gp054**	+	ttaaccatgacgggctccccg	atg	None	0	hypothetical conserved protein	
**CcrSwift_gp055**	+	cctgatgagcGGGGtggacgc	gtg	gggg	7	putative HD-domain/PDEase-like protein	SSF109604
**CcrSwift_gp056**	+	aacgatacGAGaacatccgct	atg	gag	10	hypothetical conserved protein	
**CcrSwift_gp057**	+	gctatctGGAtcgctgagccg	atg	gga	11	hypothetical conserved protein	
**CcrSwift_gp058**	+	cctgccgaagaAGGccgcctg	atg	agg	7	putative DHH phosphoesterase protein	SSF64182
**CcrSwift_gp059**	+	caagcgtcgggcGGGGacggc	gtg	gggg	5	hypothetical conserved protein	
**CcrSwift_gp060**	+	tctgtcagcAGGAGatcgccg	gtg	aggag	7	hypothetical conserved protein	
**CcrSwift_gp061**	+	ttccagcacatctAGGAcccc	atg	agga	4	hypothetical conserved protein	
**CcrSwift_gp062**	+	gattacGAGGcctatcaccac	gtg	gagg	11	hypothetical conserved protein	
**CcrSwift_gp063**	+	ttcgctgcGAGGccatttctg	atg	gagg	9	hypothetical conserved protein	
**CcrSwift_gp064**	+	aagccggcgcgcaacttccgg	atg	None	0	putative HNH endonuclease	IPR002711
**CcrSwift_gp065**	+	ccgtgggaaGAGagaaactgt	atg	gag	7	hypothetical conserved protein	
**CcrSwift_gp066**	+	acaccaaagAGGAGcaagcca	atg	aggag	7	putative major capsid protein	experimental evidence, see text
**CcrSwift_gp067**	+	atctaagcAGGAaatccccag	atg	agga	9	putative minor capsid protein	experimental evidence, see text
**CcrSwift_gp068**	+	ccccgaaacGGAaaatccccc	atg	gga	9	hypothetical conserved protein	
**CcrSwift_gp069**	+	aagcccacggcggcctaagcc	atg	None	0	hypothetical conserved protein	
**CcrSwift_gp070**	+	atcacccAGGTgaactaagcc	gtg	aggt	10	hypothetical conserved protein	

**CcrSwift_gp071**	+	caactccgctacgcctaagcc	atg	None	0	hypothetical conserved protein	
**CcrSwift_gp072**	+	ttcacgagctaaGGGGcggcc	atg	gggg	5	hypothetical conserved protein	
**CcrSwift_gp073**	+	aacccccAGGAGccctgaacc	atg	aggag	9	hypothetical conserved protein	
**CcrSwift_gp074**	+	gccgcctcaAGGgtcttcgcg	ttg	agg	9	putative lectin-like domain protein	IPR013320, IPR008985
**CcrSwift_gp075**	+	gctgacctGGAGGcctaagac	atg	ggagg	8	hypothetical conserved protein	
**CcrSwift_gp076**	+	cgtggcgatgaagttctaacg	atg	None	0	hypothetical conserved protein	
**CcrSwift_gp077**	+	tctGGAGtttcgagcgccatc	atg	ggag	14	hypothetical conserved protein	
**CcrSwift_gp078**	+	cgcaccgccAGGgctttccca	atg	agg	9	hypothetical conserved protein	
**CcrSwift_gp079**	+	ggcgtccgttcGAGagccatc	atg	gag	5	hypothetical conserved protein	
**CcrSwift_gp080**	+	agtcGGGGaccgagagccgtc	atg	gggg	13	hypothetical conserved protein	
**CcrSwift_gp081**	+	tcctgggtGGAGttttcccta	atg	ggag	9	hypothetical conserved protein	
**CcrSwift_gp082**	+	cgccatcccagagGGAGcgac	gtg	ggag	4	putative PhoH-like protein	IPR003714, SSF52540, G3DSA:3.40.50.300
**CcrSwift_gp083**	+	gtgacaaagacgtagcgcacc	atg	None	0	hypothetical conserved protein	
**CcrSwift_gp084**	+	caagaagatcattcgccacta	atg	None	0	hypothetical conserved protein	
**CcrSwift_gp085**	+	cagcacgcGGAagaagacccc	gtg	gga	10	hypothetical conserved protein	
**CcrSwift_gp086**	+	ctcgattaaaGAGGTgttcgg	atg	gaggt	6	hypothetical conserved protein	
**CcrSwift_gp087**	+	gttctggctgggaGAGGactg	atg	gagg	4	hypothetical conserved protein	
**CcrSwift_gp088**	+	ccgtGGAttggtgacctgccc	atg	gga	14	putative major tail tube protein	experimental evidence, see text
**CcrSwift_gp089**	+	cccgatacGGAacccccgaac	atg	gga	10	putative pre-tape measure chaperone protein	HHpred vs pdb70_18Aug12, hit 2ob9_A, prob 89.3% E=0.82; positional evidence, see text
**CcrSwift_gp090**	+	cccgatacGGAacccccgaac	atg	gga	10	putative pre-tape measure chaperone protein, frameshifted version	positional evidence, see text; slippery sequence AAAAAAC
**CcrSwift_gp091**	+	gcGGGGccgccgataggatca	atg	gggg	15	putative tail tape measure protein	IPR007921, IPR013491, IPR013423; experimental evidence, see text
**CcrSwift_gp092**	+	tcctttcatggttaatgcccg	atg	None	0	hypothetical conserved protein	IPR011740
**CcrSwift_gp093**	+	aaatccgtccccgaagacctg	atg	None	0	putative tail protein	IPR019228, IPR018964, IPR011928; experimental evidence, see text
**CcrSwift_gp094**	+	gcccaacgctccGGAGcagga	atg	ggag	5	putative NlpC/P60 family cell wall peptidase	IPR000064, SSF54001, G3DSA:3.90.1720.10
**CcrSwift_gp095**	+	ccccggcgttgAGGActaatc	atg	agga	6	putative tail protein	IPR007110; experimental evidence, see text
**CcrSwift_gp096**	+	tgtcataaaagtcgtggaacg	atg	None	0	hypothetical conserved protein	
**CcrSwift_gp097**	+	cctcaAGGgcgtcatcctgca	atg	agg	13	putative tail protein	IPR000421, IPR008979, G3DSA:2.60.120.260; experimental evidence, see text
**CcrSwift_gp098**	+	accctcaagGGAatccgatag	atg	gga	9	hypothetical conserved protein	
**CcrSwift_gp099**	+	tcttcatgctcGGAGcctgac	atg	ggag	6	hypothetical conserved protein	
**CcrSwift_gp100**	+	tcagcacttccGGAattccac	atg	gga	7	putative endolysin	IPR002196, SSF53955, G3DSA:1.10.530.40
**CcrSwift_gp101**	+	gggcttcaAGGgctgctgatc	atg	agg	10	putative inner membrane spanin component	N-terminal TMD, outer membrane spanin component embedded in +1 frame, see text
**CcrSwift_gp102**	+	agtaccaaGAGGTtattcacg	atg	gaggt	8	putative outer membrane spanin component	lipoprotein embedded in inner membrane spanin component, see text
**CcrSwift_gp103**	+	caagctcaaGGGGaagacgca	gtg	gggg	8	putative holin protein	predicted 4 TMDs, see text
**CcrSwift_gp104**	+	cgctatcacGGAGtagggacc	atg	ggag	8	hypothetical conserved protein	
**CcrSwift_gp105**	-	cgacggacggAGGAGaacgaa	atg	aggag	6	putative HTH domain DNA-binding protein	IPR001387, IPR010982, G3DSA:1.10.260.40
**CcrSwift_gp106**	-	ttaaccaGAGGcccgaaaata	atg	gagg	10	putative dUTP pyrophosphatase	IPR008181, IPR008180, SSF51283, PTHR11241, G3DSA:2.70.40.10
**CcrSwift_gp107**	-	tccttttcctGAGaccccttc	atg	gag	8	putative ribonucleoside diphosphate reductase beta subunit	IPR012348, IPR012335, IPR000358, IPR002109, IPR009078, IPR012336, IPR011767
**CcrSwift_gp108**	-	tcgacgcGAGaacGAGacccc	atg	gag	5	putative ribonucleoside diphosphate reductase alpha subunit	IPR006141, IPR003587, IPR000788, IPR013509, IPR008926, SSF51294, SSF51998, G3DSA:2.170.16.10, G3DSA:3.20.70.20
**CcrSwift_gp109**	-	cggtggttGGGGtggctggcg	ttg	gggg	9	hypothetical conserved protein	
**CcrSwift_gp110**	-	tcctcatgcagcAGGAatccc	atg	agga	5	hypothetical conserved protein	
**CcrSwift_gp111**	-	acgacatcaccGGAGccgagc	atg	ggag	6	hypothetical conserved protein	
**CcrSwift_gp112**	-	gacgccgaccAGGActgatcc	atg	agga	7	putative thymidylate synthase	IPR003669
**CcrSwift_gp113**	-	atccttcgctAGGAGaacccc	atg	aggag	6	putative dNMP kinase	HHpred vs pdb70_18Aug12, hit 2grj_A, prob 99.9% E=3.6E-27; SSF52540, G3DSA:3.40.50.300
**CcrSwift_gp114**	-	aagatcaAGGAcgggcaaccc	ctg	agga	10	putative RecD-like helicase	HHpred vs pdb70_18Aug12, hit 3e1s_A, prob 100% E=1.3E-45; SSF52540, G3DSA:3.40.50.300
**CcrSwift_gp115**	-	acagaaaatAGGTatcagcaa	atg	aggt	8	hypothetical conserved protein	
**CcrSwift_gp116**	-	aGAGaaatgtgacggtgcgcg	atg	gag	17	hypothetical conserved protein	
**CcrSwift_gp117**	-	caagatgcgGGAGaaggccgc	gtg	ggag	8	hypothetical conserved protein	
**CcrSwift_gp118**	-	actcccgaacGAGatcgcccg	atg	gag	8	putative exoribonuclease, Pol III-like	IPR006055, IPR013520, IPR012337, G3DSA:3.30.420.10
**CcrSwift_gp119**	-	cacctgggcGGAGacgcactg	atg	ggag	8	hypothetical conserved protein	
**CcrSwift_gp120**	-	atcaaatcctcaAGGTgctcc	atg	aggt	5	putative Pol I DNA polymerase, T7-like	IPR001098, IPR012337, SSF56672, PTHR10133, G3DSA:1.10.150.20, G3DSA:1.20.1060.10, G3DSA:3.30.420.10, G3DSA:3.30.70.370; see text
**CcrSwift_gp121**	-	acatccgttaaccatagagcc	atg	None	0	putative DNA cytosine methyltransferase	IPR001525, IPR018117, SSF53335, PTHR10629:SF11, G3DSA:3.40.50.150, G3DSA:3.90.120.10
**CcrSwift_gp122**	-	ggcgcctgtaagcGAGGtccc	atg	gagg	4	hypothetical conserved protein	
**CcrSwift_gp123**	-	gcgccaaaccGGAGcttctct	atg	ggag	7	putative T5 A1-like protein	blastP to T5 A1 (AAU05157), E=1.71E-89, 34.2% Dice identity; HHpred vs pdb70_18Aug12, hit 1g2h_A, prob 94.0% E=0.05
**CcrSwift_gp124**	-	tccggatatgccAGGAccccg	ctg	agga	5	putative DNA methylase	IPR002052, SSF53335, G3DSA:3.40.50.150
**CcrSwift_gp125**	-	cAGGAcctatgacgggttgcc	ctg	agga	16	hypothetical conserved protein	
**CcrSwift_gp126**	-	aaagatcgctatcgttgtcgc	atg	None	0	hypothetical conserved protein	
**CcrSwift_gp127**	-	tatcgcaaAGGTcatggacac	gtg	aggt	9	hypothetical conserved protein	
**CcrSwift_gp128**	-	cgttaaccacgtgcggcgccc	atg	None	0	putative DNA helicase	IPR014001, IPR001650, IPR000330, IPR014021, SSF52540, PTHR10799, G3DSA:3.40.50.300
**CcrSwift_gp129**	+	ccggtcacaAGGAGaactatc	atg	aggag	7	hypothetical conserved protein	
**CcrSwift_gp130**	-	ggaaatcaAGGAGGctgcgtg	atg	aggagg	7	hypothetical conserved protein	
**CcrSwift_gp131**	-	ccccgccgcaaAGGcccccctg	atg	agg	7	hypothetical conserved protein	
**CcrSwift_gp132**	-	acccacGAGGTctcgcgttac	ctg	gaggt	10	hypothetical conserved protein	
**CcrSwift_gp133**	-	cgccttttcaAGGAtcgcccg	atg	agga	7	hypothetical conserved protein	
**CcrSwift_gp134**	-	gccggtctcaAGGAGacgacc	atg	aggag	6	Putative rIIb-like protein	PD130832
**CcrSwift_gp135**	-	gcctgcctttcGGAGtttgcc	atg	ggag	6	putative rIIa-like protein	IPR003594
**CcrSwift_gp136**	+	ggcaaactccgaaAGGcAGGc	ttg	agg	1	hypothetical conserved protein	
**CcrSwift_gp137**	+	gcctGAGGcatgctcgtattc	atg	gagg	13	hypothetical conserved protein	
**CcrSwift_gp138**	+	cgtatggtacgcgAGGAtgac	atg	agga	4	hypothetical conserved protein	
**CcrSwift_gp139**	+	caccgacgggcgcgtcctcca	ttg	None	0	hypothetical conserved protein	
**CcrSwift_gp140**	+	acgacaacgaAGGAGGTtctc	atg	aggaggt	4	putative tyrosine recombinase	IPR013762, IPR002104, IPR011010
**CcrSwift_gp141**	+	gtcctgcgatAGGAGGgcgcg	atg	aggagg	5	hypothetical conserved protein	
**CcrSwift_gp142**	+	ccgctagtccAGGAGtacagt	ttg	aggag	6	hypothetical conserved protein	
**CcrSwift_gp143**	+	acccccgtcccgacAGGcttc	atg	agg	4	hypothetical conserved protein	
**CcrSwift_gp144**	+	atcgctcctGGGGcctttttga	gtg	gggg	8	hypothetical conserved protein	

**CcrSwift_gp145**	+	tcctgtacggtaagGGAaaac	atg	gga	4	hypothetical conserved protein	
**CcrSwift_gp146**	+	acgattcGGAGtattcatgaa	atg	ggag	10	putative HTH domain protein	IPR001387, G3DSA:1.10.260.40
**CcrSwift_gp147**	+	cctgtattcctGAGGTcccgc	atg	gaggt	5	putative tRNA amidotransferase domain protein	IPR019004, IPR003789
**CcrSwift_gp148**	-	actgatcgaGGGGaagttgag	atg	gggg	8	putative DNA ligase	IPR012340, IPR016027, SSF56091, G3DSA:3.30.470.30
**CcrSwift_gp149**	-	tcgatccGGAGattttcggcg	atg	ggag	10	hypothetical conserved protein	
**CcrSwift_gp150**	-	gccatcccctctatctccgag	gtg	None	0	hypothetical conserved protein	
**CcrSwift_gp151**	-	gtttcAGGgcaaatgtcAGGa	atg	agg	1	DUF1643 protein	IPR012441
**CcrSwift_gp152**	-	gcctcgcccttgaAGGAggca	atg	agga	4	hypothetical conserved protein	
**CcrSwift_gp153**	-	gAGGccaagaagcgggccacg	gtg	agg	17	hypothetical conserved protein	
**CcrSwift_gp154**	-	ctGGAtcgccccggcggcctg	atg	gga	16	hypothetical conserved protein	
**CcrSwift_gp155**	-	gcttttgAGGAGtttggacgt	atg	aggag	9	hypothetical conserved protein	
**CcrSwift_gp156**	-	agcaggactAGGAGaaagacc	atg	aggag	7	hypothetical conserved protein	
**CcrSwift_gp157**	-	cgacgccaAGGAcgtctgcca	atg	agga	9	hypothetical conserved protein	
**CcrSwift_gp158**	-	ggccggctacGAGatcgcctg	atg	gag	8	putative lipoprotein	LipoP
**CcrSwift_gp159**	-	cgcttaaaGGGGgacctcatc	atg	gggg	8	putative nicotinate phosphoribosyltransferase	IPR015977, IPR002638, PTHR11098
**CcrSwift_gp160**	-	cagaatgaGGGGtaagcgata	gtg	gggg	9	putative NUDIX hydrolase domain protein	IPR014729, IPR000086, IPR015797, SSF52374, PS51462, PTHR22769
**CcrSwift_gp161**	-	caaagcgtagtgtcgcgaggc	atg	None	0	hypothetical conserved protein	
**CcrSwift_gp162**	-	cgcaacgcctgGGActctctg	atg	gga	7	putative peptidyl-tRNA hydrolase	IPR002833, SSF102462, PTHR12649, G3DSA:3.40.1490.10
**CcrSwift_gp163**	-	agcgccggccgccGAGGgctg	atg	gagg	4	hypothetical conserved protein	
**CcrSwift_gp164**	-	taacctccagcAGGAGaccgc	atg	aggag	5	hypothetical conserved protein	
**CcrSwift_gp165**	-	acaacgccGGAGGccctttc	atg	ggagg	8	hypothetical conserved protein	
**CcrSwift_gp166**	-	ccccgatctccGAGaagcccc	atg	gag	7	putative HD-domain/PDEase-like protein	SSF109604
**CcrSwift_gp167**	-	gggagaattcctGAGGgtcgc	atg	gagg	5	hypothetical conserved protein	
**CcrSwift_gp168**	-	acgcccagaAGGActgatcct	atg	agga	8	putative DNA-binding domain protein	IPR016177, SSF54060
**CcrSwift_gp169**	-	gaggcctcGGAGaagtaagcc	atg	ggag	9	hypothetical conserved protein	
**CcrSwift_gp170**	-	tcttgagaAGGAacaaccctg	atg	agga	9	hypothetical conserved protein	
**CcrSwift_gp171**	-	tcaacaAGGgcaagaacgccg	atg	agg	12	hypothetical conserved protein	
**CcrSwift_gp172**	-	caagcAGGcggcgggtctgct	ttg	agg	13	hypothetical conserved protein	
**CcrSwift_gp173**	-	gctgttcgactGGAGaaaatc	gtg	ggag	6	hypothetical conserved protein	
**CcrSwift_gp174**	-	ctctgcgtgaAGGgctAGGcc	atg	agg	2	hypothetical conserved protein	
**CcrSwift_gp175**	-	acaccacagaAGGAGcccgcc	gtg	aggag	6	putative RtcB-like protein	IPR001233
**CcrSwift_gp176**	-	cgtacccctcaAGGTgatccc	atg	aggt	6	hypothetical conserved protein	
**CcrSwift_gp177**	-	cttctgaccgccgtcgccctg	atg	None	0	putative band 7 lipoprotein	IPR001107
**CcrSwift_gp178**	-	gctcctgaaAGGAcgtgctca	atg	agga	8	hypothetical conserved protein	
**CcrSwift_gp179**	-	gcagcGGGGgctcgcggcctg	atg	gggg	11	hypothetical conserved protein	
**CcrSwift_gp180**	-	acgacaacgaAGGAGacctcc	atg	aggag	6	hypothetical conserved protein	
**CcrSwift_gp181**	-	caggatGGAGGccgccgccaa	atg	ggagg	10	hypothetical conserved protein	
**CcrSwift_gp182**	-	tccgagccaAGGAGcgctgat	ttg	aggag	7	hypothetical conserved protein	
**CcrSwift_gp183**	-	cgacagcgagAGGAGccgatc	atg	aggag	6	hypothetical conserved protein	
**CcrSwift_gp184**	-	gaaacctggagaAGGAccgcc	atg	agga	5	hypothetical conserved protein	
**CcrSwift_gp185**	-	ggtcctgtcgggcgacaatcc	gtg	None	0	hypothetical conserved protein	
**CcrSwift_gp186**	-	tctgaaAGGctcgacctcctg	atg	agg	12	hypothetical conserved protein	
**CcrSwift_gp187**	-	cgttccaactcgcAGGTctcc	gtg	aggt	4	hypothetical conserved protein	
**CcrSwift_gp188**	-	cctgggcctGGAGaagcacct	gtg	ggag	8	hypothetical conserved protein	
**CcrSwift_gp189**	-	actggcgaAGGTgtcggacca	atg	aggt	9	hypothetical conserved protein	
**CcrSwift_gp190**	-	acaaccgcagAGGAGaccctc	gtg	aggag	6	hypothetical conserved protein	
**CcrSwift_gp191**	-	cgGGAtgcgttccgggtcgag	atg	gga	16	hypothetical conserved protein	
**CcrSwift_gp192**	-	gcccgagaaAGGcgaAGGccc	atg	agg	3	hypothetical conserved protein	
**CcrSwift_gp193**	-	cgccgacGGAGagctactgcg	atg	ggag	10	putative winged-helix HTH DNA binding protein	IPR011991
**CcrSwift_gp194**	-	ttacgcgccaatGAGcgccag	atg	gag	6	hypothetical conserved protein	
**CcrSwift_gp195**	-	ccacccgcgccgcccgaggag	gtg	None	0	hypothetical conserved protein	
**CcrSwift_gp196**	-	cagcgcgggcAGGgttcccgc	atg	agg	8	hypothetical conserved protein	
**CcrSwift_gp197**	-	aagaggtcgAGGAGGTcgccg	atg	aggaggt	5	hypothetical conserved protein	
**CcrSwift_gp198**	-	gacgacaacgAGGAGGccctg	atg	aggagg	5	putative ArdC-like antirestriction protein	IPR017113, IPR013610
**CcrSwift_gp199**	-	cgcggcgataatGAGcgcctg	atg	gag	6	hypothetical conserved protein	
**CcrSwift_gp200**	-	ggacctGGAGaacaaccgccg	atg	ggag	11	hypothetical conserved protein	
**CcrSwift_gp201**	-	gggccggGGAGGgcgggcatc	atg	ggagg	9	hypothetical conserved protein	
**CcrSwift_gp202**	-	cgcctgccaGAGcctcaagtc	atg	gag	9	hypothetical conserved protein	
**CcrSwift_gp203**	-	aacgacaccagggGGAGagcc	gtg	ggag	4	putative acyl carrier protein	IPR009081, IPR006163, PTHR20863
**CcrSwift_gp204**	-	tcgacaacggcaAGGAcggct	atg	agga	5	putative HNH endonuclease	IPR002711
**CcrSwift_gp205**	-	ctcattcgttaAGGAtcgacc	atg	agga	6	hypothetical conserved protein	
**CcrSwift_gp206**	-	cgttcctgggctatcaagccg	atg	None	0	hypothetical conserved protein	
**CcrSwift_gp207**	-	tggGGAGaagctgaagtgagc	atg	ggag	14	hypothetical conserved protein	
**CcrSwift_gp208**	-	cgccttcGAGGTgcaagcctg	atg	gaggt	9	hypothetical conserved protein	
**CcrSwift_gp209**	-	tccggacctcGGAGaaggccc	gtg	ggag	7	hypothetical conserved protein	
**CcrSwift_gp210**	-	gcggttcgAGGAGatcgaggc	ttg	aggag	8	hypothetical conserved protein	
**CcrSwift_gp211**	-	gacgacaacggaAGGAacctt	atg	agga	5	hypothetical conserved protein	
**CcrSwift_gp212**	-	cggtgacttcgccaAGGggac	ctg	agg	4	hypothetical conserved protein	
**CcrSwift_gp213**	-	ctatcgcttgcAGGAGGctcg	atg	aggagg	4	hypothetical conserved protein	
**CcrSwift_gp214**	-	cttcaagcgcgAGGAtcaccg	ttg	agga	6	hypothetical conserved protein	
**CcrSwift_gp215**	-	gacgacaacaAGGAGGcctcc	atg	aggagg	5	hypothetical conserved protein	
**CcrSwift_gp216**	-	ctacgtGGAGagccgggcggc	atg	ggag	11	hypothetical conserved protein	
**CcrSwift_gp217**	-	cgcgcaatttccGGAGtgacc	atg	ggag	5	hypothetical conserved protein	SSF56784
**CcrSwift_gp218**	-	ggcctgcgcgGGAGccctgtg	atg	ggag	7	hypothetical conserved protein	

**CcrSwift_gp219**	-	gagactagggtGGGGatgcga	ttg	gggg	6	hypothetical conserved protein	
**CcrSwift_gp220**	-	gatcaagtcGAGGccctgagc	atg	gagg	8	hypothetical conserved protein	
**CcrSwift_gp221**	-	ggctcccgtacAGGAtcacct	atg	agga	6	hypothetical conserved protein	
**CcrSwift_gp222**	-	caccgggctcgtggcggcctg	atg	None	0	hypothetical conserved protein	
**CcrSwift_gp223**	-	agcagaAGGAGGaagccgctc	atg	aggagg	9	putative HTH domain DNA-binding protein	IPR010982, IPR001387, G3DSA:1.10.260.40
**CcrSwift_gp224**	-	aatcgcccAGGgcgccgcctg	atg	agg	10	hypothetical conserved protein	
**CcrSwift_gp225**	-	acaacgacagaAGGAGGccgc	gtg	aggagg	4	hypothetical conserved protein	
**CcrSwift_gp226**	-	gaggcgggcgaGGGGcacgtc	gtg	gggg	6	hypothetical conserved protein	
**CcrSwift_gp227**	-	cactgtccggaAGGTcgagcc	atg	aggt	6	hypothetical conserved protein	
**CcrSwift_gp228**	-	ggtctatcgccGGAGGgcctg	atg	ggagg	5	putative Pol I DNA polymerase	IPR001098, IPR002298, SSF56672, G3DSA:3.30.70.370
**CcrSwift_gp229**	-	agatcaAGGAGtagtgcaagc	gtg	aggag	10	putative GcrA-like cell cycle regulator	IPR011681; see text
**CcrSwift_gp230**	-	gccgccaaattcGAGGggaag	atg	gagg	5	hypothetical conserved protein	
**CcrSwift_gp231**	+	cgacaacgaaAGGAGcctgcc	atg	aggag	6	hypothetical conserved protein	
**CcrSwift_gp232**	+	cgccacGAGGctcgcccaatg	atg	gagg	11	hypothetical conserved protein	
**CcrSwift_gp233**	+	caagatcGAGGaaGAGGtctg	atg	gagg	4	hypothetical conserved protein	
**CcrSwift_gp234**	+	caagctcaagaaAGGccgcct	atg	agg	6	hypothetical conserved protein	
**CcrSwift_gp235**	+	cattctcgactAGGAGGaccc	atg	aggagg	4	hypothetical conserved protein	
**CcrSwift_gp236**	+	tcgatttcctggcgggcgccg	atg	None	0	hypothetical conserved protein	
**CcrSwift_gp237**	+	gtttctggaAGGTcaggactg	atg	aggt	8	hypothetical conserved protein	
**CcrSwift_gp238**	+	agcgcgagccgcAGGActgac	atg	agga	5	hypothetical conserved protein	
**CcrSwift_gp239**	+	cgacagcgaaAGGAGcctgcc	ttg	aggag	6	hypothetical conserved protein	
**CcrSwift_gp240**	+	tcggatgctGGAGGTcgcggc	ttg	ggaggt	6	hypothetical conserved protein	
**CcrSwift_gp241**	+	cgccgtggAGGAGatgcgccg	gtg	aggag	8	hypothetical conserved protein	
**CcrSwift_gp242**	+	cgccgacttcaAGGAcaagcg	atg	agga	6	hypothetical conserved protein	
**CcrSwift_gp243**	+	gccgccGGAGGcagacccgac	gtg	ggagg	10	hypothetical conserved protein	
**CcrSwift_gp244**	+	tcaagctGGAGaccgtctgac	atg	ggag	10	hypothetical conserved protein	
**CcrSwift_gp245**	+	acgacaacgagAGGAGttccc	atg	aggag	5	hypothetical conserved protein	
**CcrSwift_gp246**	+	cacggaatgGGAGattttcga	atg	ggag	8	hypothetical conserved protein	
**CcrSwift_gp247**	+	ggcctGGAGatcatcgcctag	atg	ggag	12	hypothetical conserved protein	
**CcrSwift_gp248**	+	acctcggacgAGGAGGactaa	atg	aggagg	5	hypothetical conserved protein	
**CcrSwift_gp249**	+	ggcccccgAGGAacccaaagc	atg	agga	9	hypothetical conserved protein	
**CcrSwift_gp250**	+	ggtcagccAGGAcatgccgtc	atg	agga	9	hypothetical conserved protein	
**CcrSwift_gp251**	+	gacgacaacgAGGAGGcttcg	atg	aggagg	5	hypothetical conserved protein	
**CcrSwift_gp252**	+	gtcaaagAGGAGGcctgagcc	ttg	aggagg	8	hypothetical conserved protein	
**CcrSwift_gp253**	+	ggcgccctacGAGGccccgca	atg	gagg	7	hypothetical conserved protein	
**CcrSwift_gp254**	+	ccggcgAGGAGttgcgctgac	atg	aggag	10	hypothetical conserved protein	
**CcrSwift_gp255**	+	tggacgacaacAGGAGGgacc	atg	aggagg	4	hypothetical conserved protein	
**CcrSwift_gp256**	+	ctacgtgaAGGAcctgcgtcg	atg	agga	9	hypothetical conserved protein	
**CcrSwift_gp257**	+	tcagcgtGGGGgtggctgctg	atg	gggg	9	hypothetical conserved protein	
**CcrSwift_gp258**	+	gacccaGGGGgtttccgaggc	atg	gggg	10	hypothetical conserved protein	
**CcrSwift_gp259**	+	cctcgacgcgAGGAaaagctg	atg	agga	7	hypothetical conserved protein	
**CcrSwift_gp260**	+	cctgaAGGAaggcggaaccca	atg	agga	12	hypothetical conserved protein	
**CcrSwift_gp261**	+	ttgtcttGAGaaagccagcgc	atg	gag	11	DUF1937 protein	IPR015235, SSF52309
**CcrSwift_gp262**	+	gagcaccagGGAGattgaagc	gtg	ggag	8	hypothetical conserved protein	
**CcrSwift_gp263**	+	ggtgtcccaGAGGTaccagcg	atg	gaggt	7	hypothetical conserved protein	
**CcrSwift_gp264**	+	ccggcgcctatGGAGagagcc	atg	ggag	6	putative TAT signal protein	IPR006311; PRED-TAT
**CcrSwift_gp265**	+	cctGGAtatggcgaagaccgc	atg	gga	15	hypothetical conserved protein	
**CcrSwift_gp266**	+	acgtcaacaAGGAGcccaccg	atg	aggag	7	hypothetical conserved protein	
**CcrSwift_gp267**	+	agaagcccggcgggccgagtg	atg	None	0	hypothetical conserved protein	
**CcrSwift_gp268**	+	gcggtcgaaatcctGGAgacc	ctg	gga	4	hypothetical conserved protein	
**CcrSwift_gp269**	+	gccgctcgatGGAtGGAacgg	atg	gga	4	hypothetical conserved protein	
**CcrSwift_gp270**	+	cgaggaagAGGAGcgctgctg	atg	aggag	8	putative Ser/Thr protein phosphatase	IPR004843, IPR006186, SSF56300, PTHR11668, G3DSA:3.60.21.10
**CcrSwift_gp271**	+	gttgatccgcgAGGAcgacgc	atg	agga	6	hypothetical conserved protein	
**CcrSwift_gp272**	+	gacgacaacgAGGAGGcccac	atg	aggagg	5	hypothetical conserved protein	
**CcrSwift_gp273**	+	catgaccaaGAGccgcttctg	atg	gag	9	hypothetical conserved protein	
**CcrSwift_gp274**	+	gatcatcctgGGAGacgccgc	atg	ggag	7	hypothetical conserved protein	
**CcrSwift_gp275**	+	cgacgacaacgAGGAGGcccc	atg	aggagg	4	hypothetical conserved protein	
**CcrSwift_gp276**	+	cttcgtctcgggtgaagcctg	atg	None	0	hypothetical conserved protein	
**CcrSwift_gp277**	+	gccacggaagAGGAcgcctag	atg	agga	7	hypothetical conserved protein	
**CcrSwift_gp278**	+	acgagcgacgGGAGGgctgac	atg	ggagg	6	hypothetical conserved protein	
**CcrSwift_gp279**	+	tccgaaaGGGGccttaagtcc	atg	gggg	10	hypothetical conserved protein	
**CcrSwift_gp280**	+	aacggaAGGAcctgaaagacc	atg	agga	11	hypothetical conserved protein	
**CcrSwift_gp281**	+	acaacgccGGAGGccctttttc	atg	ggagg	8	hypothetical conserved protein	
**CcrSwift_gp282**	+	cgcctttgcgaacctcgccca	atg	None	0	hypothetical conserved protein	
**CcrSwift_gp283**	+	acgccaagaAGGccgcctgac	atg	agg	9	hypothetical conserved protein	
**CcrSwift_gp284**	+	ccagatcaAGGAGGcccagtc	ttg	aggagg	7	hypothetical conserved protein	
**CcrSwift_gp285**	+	caatcagcaataGGGGccagc	atg	gggg	5	hypothetical conserved protein	
**CcrSwift_gp286**	+	ctgggccGAGGgctcgccgcg	atg	gagg	10	hypothetical conserved protein	
**CcrSwift_gp287**	+	cggacatagAGGAGcaccccg	atg	aggag	7	hypothetical conserved protein	
**CcrSwift_gp288**	+	tcgccgcctgAGGAGGacccg	atg	aggagg	5	hypothetical conserved protein	
**CcrSwift_gp289**	+	gcttgagaAGGcgttcggcca	atg	agg	10	hypothetical conserved protein	
**CcrSwift_gp290**	+	ccaagcctcGGGGctgttcca	atg	gggg	8	hypothetical conserved protein	
**CcrSwift_gp291**	+	caccgactactAGGAGGtccc	atg	aggagg	4	hypothetical conserved protein	
**CcrSwift_gp292**	+	tcgctaggacaAGGAGGcccc	atg	aggagg	4	hypothetical conserved protein	

**CcrSwift_gp293**	+	acgacaacAGGAGGccccgcc	ttg	aggagg	7	hypothetical conserved protein	
**CcrSwift_gp294**	+	ttgcggtcatgAGGAttcgaa	atg	agga	6	hypothetical conserved protein	
**CcrSwift_gp295**	+	ccggcggctgacgggtgcgtc	atg	None	0	hypothetical conserved protein	
**CcrSwift_gp296**	+	gacgacaacgAGGAGGccccc	atg	aggagg	5	hypothetical conserved protein	
**CcrSwift_gp297**	+	ggactacatcGAGGTgaccct	gtg	gaggt	6	hypothetical conserved protein	
**CcrSwift_gp298**	+	ccttagccgGGAGGcgcagcc	ttg	ggagg	7	hypothetical conserved protein	
**CcrSwift_gp299**	+	gacggcGGAGGccctggcctg	atg	ggagg	10	hypothetical conserved protein	
**CcrSwift_gp300**	+	ggaacAGGAGaccgccgcctg	atg	aggag	11	hypothetical conserved protein	
**CcrSwift_gp301**	+	gatcgcgcgactGGAGaacgc	atg	ggag	5	hypothetical conserved protein	
**CcrSwift_gp302**	+	cggcgggatcaAGGTgagcgc	atg	aggt	6	hypothetical conserved protein	
**CcrSwift_gp303**	+	tttcatcgccGAGGccctgtc	atg	gagg	7	hypothetical conserved protein	
**CcrSwift_gp304**	+	acgctgcgaAGGAGcctcgac	ttg	aggag	7	hypothetical conserved protein	
**CcrSwift_gp305**	+	ccagatcAGGAaggccgccgc	atg	agga	10	hypothetical conserved protein	
**CcrSwift_gp306**	+	tcgcgacacggcaAGGTacgc	atg	aggt	4	hypothetical conserved protein	
**CcrSwift_gp307**	+	cggcctgcatgcGAGGggctg	atg	gagg	5	hypothetical conserved protein	
**CcrSwift_gp308**	+	gatctacaagccggtgcgctg	atg	None	0	hypothetical conserved protein	
**CcrSwift_gp309**	+	gctcgccaAGGAcgtggtgtg	atg	agga	9	hypothetical conserved protein	
**CcrSwift_gp310**	+	ccgcctGGAGcttgccgccac	atg	ggag	11	hypothetical conserved protein	
**CcrSwift_gp311**	+	cctcggctGGAagtcctcgtc	atg	gga	10	hypothetical conserved protein	
**CcrSwift_gp312**	+	cctggagtatcGGGGtcgggc	gtg	gggg	6	putative HNH homing endonuclease	IPR002711
**CcrSwift_gp313**	+	aaccgggctagaccGGAggcc	atg	gga	4	hypothetical conserved protein	
**CcrSwift_gp314**	+	gatccttccGGAGGacgcggc	gtg	ggagg	7	hypothetical conserved protein	
**CcrSwift_gp315**	+	caggctgaaGAGaccgggcgg	atg	gag	9	hypothetical conserved protein	
**CcrSwift_gp316**	+	caccctcaacaAGGAGcccct	gtg	aggag	5	hypothetical conserved protein	
**CcrSwift_gp317**	+	ccggtcctgaGAGGccccaag	atg	gagg	7	hypothetical conserved protein	
**CcrSwift_gp318**	+	taagaatgAGGAaatacatcc	atg	agga	9	hypothetical conserved protein	
**CcrSwift_gp319**	+	aaggcccctcgGGAGccaccc	atg	ggag	6	hypothetical conserved protein	
**CcrSwift_gp320**	+	ccatcgcctGAGaccctcccc	atg	gag	9	hypothetical conserved protein	
**CcrSwift_gp321**	+	cgccgacGGGGtcgacctgac	gtg	gggg	10	putative RNaseH-like domain protein	IPR012337
**CcrSwift_gp322**	+	cgacccggcGGAGtttccggc	ttg	ggag	8	hypothetical conserved protein	
**CcrSwift_gp323**	+	aagaagacGAGGacGAGGacg	atg	gagg	3	hypothetical conserved protein	
**CcrSwift_gp324**	+	aaacgcaccGAGctttagatc	atg	gag	9	Terminase small subunit	HHpred vs pdb70_18Aug12, hit 3zqp_A, prob 90.4% E=0.15; position relative to terminase large subunit, see text
**CcrSwift_gp325**	+	tcgcgctgaccctagaacgcc	gtg	None	0	Terminase large subunit	IPR004921, IPR007868, IPR006141, IPR003587, IPR006517, SSF51294, G3DSA:2.170.16.10

Table S5. Predicted proteins, gene starts and annotations of *C. crescentus* phage CcrRogue. Upstream sequence is the 25 bp of DNA sequence upstream of the start codon, with the predicted Shine-Dalgarno motif capitalized. S-D is the Shine-Dalgarno motif associated with the gene start, and spacing is the interval (in bp) between the S-D and start codon. Evidence includes detected conserved domains or motifs, BlastP and HHpred hits, and experimental or other evidence as detailed in the text.

Protein name	Strand	Upstream Sequence	Start Codon	S-D	spacing	Predicted product	Evidence
**CcrRogue_gp001**	+	tggcaaatcaAGGAGccggcg	atg	aggag	6	hypothetical conserved protein	
**CcrRogue_gp002**	+	cgaaatcaaAGGcttatcgcc	atg	agg	9	hypothetical conserved protein	
**CcrRogue_gp003**	+	atcccgttaccGGAcaacccc	atg	gga	7	hypothetical conserved protein	
**CcrRogue_gp004**	-	cgcgcccttGGAGaacgcccc	atg	ggag	8	hypothetical conserved protein	
**CcrRogue_gp005**	+	tttgacattgtGAGGcaaccc	atg	gagg	6	hypothetical conserved protein	
**CcrRogue_gp006**	+	aattccggcctcttgcgcctt	atg	None	0	hypothetical novel protein	
**CcrRogue_gp007**	+	aaccccctGGAGGccctgcccc	atg	ggagg	9	hypothetical conserved protein	
**CcrRogue_gp008**	+	cgacgccgccaAGGTgaaagc	gtg	aggt	6	hypothetical conserved protein	
**CcrRogue_gp009**	+	cccggccttGGAGttcttccc	atg	ggag	8	hypothetical conserved protein	
**CcrRogue_gp010**	+	tccgtgaaAGGAatccatacc	gtg	agga	9	hypothetical conserved protein	
**CcrRogue_gp011**	+	cctcacctGGAGattttcccc	atg	ggag	9	hypothetical conserved protein	
**CcrRogue_gp012**	+	gccgcactcGGAaccaagccc	atg	gga	9	hypothetical conserved protein	
**CcrRogue_gp013**	+	cccccgcaAGGAtcgcccgac	atg	agga	9	hypothetical conserved protein	
**CcrRogue_gp014**	+	gaaccgaAGGAaccccgcccc	atg	agga	10	hypothetical conserved protein	
**CcrRogue_gp015**	+	ccctgccgaAGGTcgcctaac	atg	aggt	8	hypothetical conserved protein	
**CcrRogue_gp016**	+	cacccgaaAGGAcgcctgagc	atg	agga	9	hypothetical conserved protein	
**CcrRogue_gp017**	+	tccttctctGGAGccgctacc	atg	ggag	8	hypothetical conserved protein	
**CcrRogue_gp018**	+	tcctctcttGGAtcgctccac	atg	gga	9	hypothetical conserved protein	
**CcrRogue_gp019**	+	gggtcgcaAGGAGacacccac	atg	aggag	8	hypothetical conserved protein	
**CcrRogue_gp020**	+	aacgcaagcccAGGAGGctcc	atg	aggagg	4	hypothetical conserved protein	
**CcrRogue_gp021**	+	cgtcacggcctgatcgtcacc	atg	None	0	hypothetical conserved protein	
**CcrRogue_gp022**	+	cggcgcctGGGGcaaggtctg	atg	gggg	9	hypothetical conserved protein	
**CcrRogue_gp023**	+	tcaagcctGGAttctcatctg	atg	gga	10	putative TAT signal protein	PRED-TAT
**CcrRogue_gp024**	+	gaagaAGGctctggtcgcctg	atg	agg	13	putative lipoprotein	LipoP
**CcrRogue_gp025**	+	tgtcgacaccccggcccgctg	atg	None	0	putative HTH homeodomain DNA binding protein	IPR009057
**CcrRogue_gp026**	+	cAGGAagctggaagccaagac	atg	agga	16	hypothetical novel protein	
**CcrRogue_gp027**	+	aagccgGGAccagaaaatctg	atg	gga	12	hypothetical novel protein	
**CcrRogue_gp028**	+	cctggaaaaccgcGAGcccgc	atg	gag	5	hypothetical novel protein	
**CcrRogue_gp029**	+	cgcccggcggccgttggcggc	atg	None	0	hypothetical conserved protein	
**CcrRogue_gp030**	+	cccgatcGGAGaatcctgacc	atg	ggag	10	hypothetical conserved protein	
**CcrRogue_gp031**	+	agaccatcttcGAGGcgacct	atg	gagg	6	hypothetical conserved protein	
**CcrRogue_gp032**	+	gccgcccaAGGAtgccgccgc	gtg	agga	9	hypothetical conserved protein	
**CcrRogue_gp033**	+	ggtccgcaAGGAGcgcctcta	ttg	aggag	8	hypothetical conserved protein	
**CcrRogue_gp034**	+	actacaacaAGGcctgatccc	atg	agg	9	hypothetical conserved protein	
**CcrRogue_gp035**	+	cgcgaaagccGGGGttaacct	gtg	gggg	7	hypothetical novel protein	
**CcrRogue_gp036**	+	gacccggatgcGGAGGgcgtc	gtg	ggagg	5	putative HTH DNA binding protein	IPR001387, IPR010982, G3DSA:1.10.260.40
**CcrRogue_gp037**	+	ccctgagttcaAGGcctgatc	atg	agg	7	hypothetical novel protein	
**CcrRogue_gp038**	+	caagctGGAGaagggtgaggc	gtg	ggag	11	hypothetical novel protein	
**CcrRogue_gp039**	+	gcagcagccgcAGGAagagtc	atg	agga	6	hypothetical conserved protein	
**CcrRogue_gp040**	+	caacggcgatctggtgaaatt	gtg	None	0	hypothetical novel protein	
**CcrRogue_gp041**	+	gcgcaacgccctGGGGgttct	gtg	gggg	4	hypothetical novel protein	
**CcrRogue_gp042**	+	ctatcgcttGAGGgacgccga	gtg	gagg	8	hypothetical conserved protein	
**CcrRogue_gp043**	-	cgggtatctcaAGGAGtagcc	atg	aggag	5	hypothetical conserved protein	
**CcrRogue_gp044**	-	tgacatttcGGGGgtgtcgtc	atg	gggg	7	putative transglutaminase-like cysteine peptidase	IPR010319
**CcrRogue_gp045**	-	ttgacgtgGGAagcccgccgg	atg	gga	10	hypothetical novel protein	
**CcrRogue_gp046**	+	gcgacctcgcAGGcgatctat	ctg	agg	8	putative portal protein	HHpred vs pdb70_18Aug12, hit 2jes_A, prob 99.9% E=1.7E-23; positional evidence, see text
**CcrRogue_gp047**	+	atccagcaagcctGAGttcag	atg	gag	5	hypothetical conserved protein	
**CcrRogue_gp048**	+	gcgcccgaagaaagcggcctg	atg	None	0	hypothetical conserved protein	
**CcrRogue_gp049**	+	gttaaccacGAGGgcttttttc	atg	gagg	8	hypothetical conserved protein	
**CcrRogue_gp050**	+	caccccttccaaGAGGccctcg	atg	gagg	6	hypothetical conserved protein	
**CcrRogue_gp051**	+	cgcgtccGGGGgacctgaccc	atg	gggg	9	hypothetical novel protein	
**CcrRogue_gp052**	+	cacctgcgtcGAGGcctgatc	atg	gagg	7	hypothetical conserved protein	
**CcrRogue_gp053**	+	gatcgccgcctccgtcaagac	gtg	None	0	hypothetical conserved protein	
**CcrRogue_gp054**	+	catctacgaaagcaccccgga	atg	None	0	hypothetical conserved protein	
**CcrRogue_gp055**	+	gctcacgttccgGGAGacgcc	gtg	ggag	5	hypothetical conserved protein	
**CcrRogue_gp056**	+	gccgatcccGGAcggcgccct	gtg	gga	9	hypothetical conserved protein	
**CcrRogue_gp057**	+	ttaaccacgacgggctctccg	atg	None	0	hypothetical conserved protein	
**CcrRogue_gp058**	+	accgagatcaaGGGGtaggcc	atg	gggg	6	putative HD-domain/PDEase-like protein	SSF109604
**CcrRogue_gp059**	+	gaacgatacGAGaacatgccc	atg	gag	9	hypothetical conserved protein	
**CcrRogue_gp060**	+	gctatctcgaccgctgatccg	atg	None	0	hypothetical conserved protein	
**CcrRogue_gp061**	+	catgcctAGGAaggccgcctg	atg	agga	10	putative HNH homing endonuclease	IPR001982, G3DSA:3.10.28.10, SSF55608
**CcrRogue_gp062**	+	aatgaacaagaGAGGcctata	atg	gagg	6	putative DHH phosphoesterase protein	SSF64182
**CcrRogue_gp063**	+	cccgtcgcgcgggcaagacca	atg	None	0	hypothetical novel protein	
**CcrRogue_gp064**	+	agtttgagcAGGAGGTcggcg	gtg	aggaggt	5	hypothetical conserved protein	
**CcrRogue_gp065**	+	ttccagcacatctGAGGtccc	atg	gagg	4	hypothetical conserved protein	
**CcrRogue_gp066**	+	tacGAGGcctaccatcacatc	atg	gagg	14	hypothetical conserved protein	
**CcrRogue_gp067**	+	gtccatcaAGGgctccttctg	atg	agg	10	hypothetical conserved protein	
**CcrRogue_gp068**	+	aagcccccgcgcaactaccgg	atg	None	0	putative HNH endonuclease	IPR002711
**CcrRogue_gp069**	+	tccgtggaaaGAGagaaacct	atg	gag	6	hypothetical conserved protein	
**CcrRogue_gp070**	+	aacaccaaaAGGAGcaagcca	atg	aggag	7	putative major capsid protein	experimental evidence, see text
**CcrRogue_gp071**	+	atctaagcAGGAactccccag	atg	agga	9	putative minor capsid protein	experimental evidence, see text
**CcrRogue_gp072**	+	ccccgaaacGGAtatttcccc	atg	gga	9	hypothetical conserved protein	
**CcrRogue_gp073**	+	cgcaAGGcgggcgcctaagcc	atg	agg	14	hypothetical conserved protein	

**CcrRogue_gp074**	+	atcacccAGGTgaactaaccc	gtg	aggt	10	hypothetical conserved protein	
**CcrRogue_gp075**	+	caactgcgctacgcctaaccc	atg	None	0	hypothetical conserved protein	
**CcrRogue_gp076**	+	tcacgaactaaGGGGgcggcc	atg	gggg	5	hypothetical conserved protein	
**CcrRogue_gp077**	+	taaccctcAGGAGccccaacc	atg	aggag	8	hypothetical conserved protein	
**CcrRogue_gp078**	+	tcccgtcaaAGGTctcccgcg	ttg	aggt	8	putative lectin-like domain protein	IPR013320, IPR008985
**CcrRogue_gp079**	+	gcgaccctGGAGacctaaggc	atg	ggag	9	hypothetical conserved protein	
**CcrRogue_gp080**	+	ttccggtccGAGGgccgccgc	gtg	gagg	8	hypothetical conserved protein	
**CcrRogue_gp081**	+	tctGGAGtttcgagcgccacc	atg	ggag	14	hypothetical conserved protein	
**CcrRogue_gp082**	+	gcctgcatgcacGAGGctacc	atg	gagg	5	hypothetical conserved protein	
**CcrRogue_gp083**	+	ggtggccagtcGAGagttgtc	atg	gag	5	hypothetical conserved protein	
**CcrRogue_gp084**	+	aagctggatGAGcttggcgca	atg	gag	9	hypothetical conserved protein	
**CcrRogue_gp085**	+	tgttcctggGGAGtttcctag	atg	ggag	8	hypothetical conserved protein	
**CcrRogue_gp086**	+	cgccatcccagagGGAGcgac	gtg	ggag	4	putative PhoH-like protein	IPR003714, SSF52540, G3DSA:3.40.50.300
**CcrRogue_gp087**	+	gtgacaaagacgtaGAGcacc	atg	gag	4	hypothetical conserved protein	
**CcrRogue_gp088**	+	caagaagatcatccggcacta	atg	None	0	hypothetical conserved protein	
**CcrRogue_gp089**	+	cacctcccGGAtcgtgacgcc	gtg	gga	10	hypothetical conserved protein	
**CcrRogue_gp090**	+	ttctggctcggGGAGGactag	atg	ggagg	5	hypothetical conserved protein	
**CcrRogue_gp091**	+	ccctGGAttggtgacctgcac	atg	gga	14	putative major tail tube protein	experimental evidence, see text
**CcrRogue_gp092**	+	cgatacatGGActccccgaaa	atg	gga	10	putative pre-tape measure chaperone protein	HHpred vs pdb70_18Aug12, hit 2ob9_A, prob 86.0% E=2.3; positional evidence, see text
**CcrRogue_gp093**	+	cgatacatGGActccccgaaa	atg	gga	10	putative pre-tape measure chaperone protein, frameshifted version	positional evidence, see text; slippery sequence AAAAAAC
**CcrRogue_gp094**	+	acGGGGccgccgataggatca	atg	gggg	15	putative tail tape measure protein	IPR007921, IPR013491, IPR013423; experimental evidence, see text
**CcrRogue_gp095**	+	atccagGGActcccttctcgc	atg	gga	12	hypothetical conserved protein	IPR011740
**CcrRogue_gp096**	+	ctatcAGGAGatcatcctccc	gtg	aggag	11	putative tail protein	IPR019228, IPR018964, IPR011928; experimental evidence, see text
**CcrRogue_gp097**	+	gcccaacgcgccGGAGcagga	atg	ggag	5	putative NlpC/P60 family cell wall peptidase	IPR000064, SSF54001, G3DSA:3.90.1720.10
**CcrRogue_gp098**	+	ccccggcgttgAGGActaagc	atg	agga	6	putative tail protein	IPR007110; experimental evidence, see text
**CcrRogue_gp099**	+	tgtcataaaagccgtggaacg	atg	None	0	hypothetical conserved protein	
**CcrRogue_gp100**	+	cctcaAGGgcgtcatcctgca	atg	agg	13	putative tail protein	IPR000421, IPR008979, G3DSA:2.60.120.260; experimental evidence, see text
**CcrRogue_gp101**	+	agcactcccGGAattccccac	atg	gga	9	putative endolysin	IPR002196, SSF53955, G3DSA:1.10.530.40
**CcrRogue_gp102**	+	aagccttcaAGGgctgctgac	atg	agg	9	putative inner membrane spanin component	N-terminal TMD, outer membrane spanin component embedded in +1 frame, see text
**CcrRogue_gp103**	+	agtaccaaGAGGTtatccaca	atg	gaggt	8	putative outer membrane spanin component	lipoprotein embedded in inner membrane spanin component, see text
**CcrRogue_gp104**	+	caagttcaaGGGGaagaagtg	atg	gggg	8	putative holin protein	predicted 4 TMDs, see text
**CcrRogue_gp105**	+	cgctatcacGGAGtagggacc	atg	ggag	8	hypothetical novel protein	
**CcrRogue_gp106**	-	caacggacggAGGAGaacgat	atg	aggag	6	putative HTH domain DNA-binding protein	IPR001387, IPR010982, IPR010982, G3DSA:1.10.260.40
**CcrRogue_gp107**	-	ttaaccaGAGGcccgaaaata	atg	gagg	10	putative dUTP pyrophosphatase	IPR008181, IPR008180, SSF51283, PTHR11241, G3DSA:2.70.40.10
**CcrRogue_gp108**	-	tcctttcGAGacctcccacac	atg	gag	11	putative ribonucleoside diphosphate reductase beta subunit	IPR012348, IPR012335, IPR000358, IPR002109, IPR009078, IPR012336, IPR011767
**CcrRogue_gp109**	-	ttgtcgcGAGaacGAGacccc	atg	gag	5	putative ribonucleoside diphosphate reductase alpha subunit	IPR006141, IPR003587, IPR000788, IPR013509, IPR008926, SSF51294, SSF51998, G3DSA:2.170.16.10, G3DSA:3.20.70.20
**CcrRogue_gp110**	-	gcatcgttGGGGtccagggcg	ttg	gggg	9	hypothetical conserved protein	
**CcrRogue_gp111**	-	tcctcatgcagcAGGAatccc	atg	agga	5	hypothetical conserved protein	
**CcrRogue_gp112**	-	cgacatcccccGGAGccgccc	atg	ggag	6	hypothetical conserved protein	
**CcrRogue_gp113**	-	tcgtcttcgacaAGGActgac	atg	agga	5	putative thymidylate synthase	IPR003669
**CcrRogue_gp114**	-	gtccttcgctAGGAGaccccg	atg	aggag	6	putative dNMP kinase	HHpred vs pdb70_18Aug12, hit 2grj_A, prob 99.9% E=7.3E-24; SSF52540, G3DSA:3.40.50.300
**CcrRogue_gp115**	-	aagatcaAGGAcggccagcct	ctg	agga	10	putative RecD-like helicase	HHpred vs pdb70_18Aug12, hit 3e1s_A, prob 100% E=4E-47; SSF52540, G3DSA:3.40.50.300
**CcrRogue_gp116**	-	cagaaaatAGGcatcagcact	atg	agg	10	hypothetical conserved protein	
**CcrRogue_gp117**	-	tttatgacggcggtgcgctag	atg	None	0	hypothetical conserved protein	
**CcrRogue_gp118**	-	ctcccgaacGAGatcgccccg	atg	gag	9	putative exoribonuclease, Pol III-like	IPR006055, IPR013520, IPR012337, G3DSA:3.30.420.10
**CcrRogue_gp119**	-	cacctgggccGAGacgcactg	atg	gag	8	hypothetical conserved protein	
**CcrRogue_gp120**	-	accagattctcaAGGTgctcc	atg	aggt	5	putative Pol I DNA polymerase, T7-like	IPR001098, IPR012337, SSF56672, PTHR10133, G3DSA:1.10.150.20, G3DSA:1.20.1060.10, G3DSA:3.30.420.10, G3DSA:3.30.70.370; see text
**CcrRogue_gp121**	-	gacatcggttaaccatacctc	atg	None	0	putative DNA cytosine methyltransferase	IPR001525, IPR018117, SSF53335, PTHR10629:SF11, G3DSA:3.40.50.150, G3DSA:3.90.120.10
**CcrRogue_gp122**	-	ggcacctgtaacGAGGccccg	atg	gagg	5	hypothetical conserved protein	
**CcrRogue_gp123**	-	gcgccaaaccGGAGcttctct	atg	ggag	7	putative T5 A1-like protein	blastP to T5 A1 (AAU05157), E=7.75E-85, 30.8% Dice identity; HHpred vs pdb70_18Aug12, hit 1qgp_A, prob 96.5% E=0.0028
**CcrRogue_gp124**	-	tcgggctatgccAGGAacccc	ttg	agga	5	putative DNA methylase	IPR002052, SSF53335, G3DSA:3.40.50.150
**CcrRogue_gp125**	-	taggcgcgGGActgcaaatct	ctg	gga	10	hypothetical novel protein	
**CcrRogue_gp126**	+	gactttcAGGAGatttgtgtc	gtg	aggag	9	putative cadherin-like domain protein	IPR002126, IPR006626, IPR015919 PTHR10596
**CcrRogue_gp127**	-	tttGAGtaagtgtcccaaccc	atg	gag	15	hypothetical conserved protein	
**CcrRogue_gp128**	-	catcgcgaAGGTcatggacac	gtg	aggt	9	hypothetical conserved protein	
**CcrRogue_gp129**	-	gcgttaaccacgtgcggaccc	atg	None	0	putative DNA helicase	IPR014001, IPR001650, IPR000330, IPR014021, SSF52540, PTHR10799, G3DSA:3.40.50.300
**CcrRogue_gp130**	+	ccggtcacaAGGAGaactacc	atg	aggag	7	hypothetical conserved protein	
**CcrRogue_gp131**	-	ggaaattaAGGAGGcggcgtg	atg	aggagg	7	hypothetical conserved protein	
**CcrRogue_gp132**	-	agaagtacaagaagtgagcca	atg	None	0	hypothetical conserved protein	
**CcrRogue_gp133**	-	tgcaagacctgcgggcggacg	ctg	None	0	hypothetical conserved protein	
**CcrRogue_gp134**	-	ccacttcagaAGGAtcgcccc	atg	agga	7	hypothetical conserved protein	
**CcrRogue_gp135**	-	gccgcgagcaAGGAGtctccc	atg	aggag	6	Putative rIIb-like protein	PD130832
**CcrRogue_gp136**	-	cactctcctctGGAGttgata	atg	ggag	6	putative rIIa-like protein	IPR003594
**CcrRogue_gp137**	+	actccagAGGAGagtggttag	gtg	aggag	9	hypothetical conserved protein	
**CcrRogue_gp138**	+	tcaatggtacgcgAGGAcgac	atg	agga	4	hypothetical conserved protein	
**CcrRogue_gp139**	+	ctccgacgggcgcgtgctcaa	ttg	None	0	hypothetical conserved protein	
**CcrRogue_gp140**	+	acgacaacgaAGGAGGctttc	atg	aggagg	5	putative tyrosine recombinase	IPR013762, IPR002104, IPR011010
**CcrRogue_gp141**	+	gtcctgcgataGGGGgcggcg	atg	gggg	5	hypothetical conserved protein	
**CcrRogue_gp142**	+	gcctgaagtaAGGAGtacagc	ttg	aggag	6	hypothetical conserved protein	
**CcrRogue_gp143**	+	gggcgcggtggtcgcggcctg	atg	None	0	hypothetical novel protein	
**CcrRogue_gp144**	+	acccccgtcccgacAGGcttc	atg	agg	4	hypothetical conserved protein	
**CcrRogue_gp145**	-	ttcccgcctctGGAGattttc	atg	ggag	6	hypothetical novel protein	
**CcrRogue_gp146**	+	cttgtacggtaagGGAaaccg	atg	gga	5	hypothetical conserved protein	
**CcrRogue_gp147**	+	ccgagacGGAGtattcatgag	atg	ggag	10	putative HTH domain protein	IPR001387, G3DSA:1.10.260.40
**CcrRogue_gp148**	+	tctctacgcctgaAGGAcccc	atg	agga	4	putative tRNA amidotransferase domain protein	IPR019004, IPR003789
**CcrRogue_gp149**	-	ccaagcaagAGGAcaccgacg	atg	agga	8	hypothetical novel protein	
**CcrRogue_gp150**	-	gttgatcgaGGGGaaattgag	atg	gggg	8	putative DNA ligase	IPR012340, IPR016027, SSF56091, G3DSA:3.30.470.30

**CcrRogue_gp151**	-	tcctcaacccGGAcatcttcg	atg	gga	8	hypothetical conserved protein	
**CcrRogue_gp152**	-	ggggcgcctagcgcggcgctg	atg	None	0	hypothetical conserved protein	
**CcrRogue_gp153**	-	gtctcGGGGcaaatgtcagga	atg	gggg	12	DUF1643 protein	IPR012441
**CcrRogue_gp154**	-	gcacgcgcctaagccgctctg	atg	None	0	hypothetical conserved protein	
**CcrRogue_gp155**	-	catcagtAGGAGtttggacgt	atg	aggag	9	hypothetical conserved protein	
**CcrRogue_gp156**	-	aacaggaatAGGAGcagtccc	atg	aggag	7	hypothetical conserved protein	
**CcrRogue_gp157**	-	cgaccccgtgaAGGTctgcca	atg	aggt	6	hypothetical conserved protein	
**CcrRogue_gp158**	-	cttcgtcgacGAGcccgcctg	atg	gag	8	hypothetical conserved protein	
**CcrRogue_gp159**	-	caacgcttaaaGGGGaacctc	atg	gggg	6	putative nicotinate phosphoribosyltransferase	IPR015977, IPR002638, PTHR11098
**CcrRogue_gp160**	-	cccggcctttAGGAGGTgatg	gtg	aggaggt	4	putative NUDIX hydrolase domain protein	IPR014729, IPR000086, IPR015797, SSF52374, PS51462, PTHR22769
**CcrRogue_gp161**	-	aaagccGGAGGgaaatcgata	gtg	ggagg	10	hypothetical conserved protein	
**CcrRogue_gp162**	-	ttgcacgcctgGGAcctcgtg	atg	gga	7	putative peptidyl-tRNA hydrolase	IPR002833, SSF102462, PTHR12649, G3DSA:3.40.1490.10
**CcrRogue_gp163**	-	ccgacgcccGAGGcctaatcg	gtg	gagg	8	hypothetical conserved protein	
**CcrRogue_gp164**	-	agcaagtacactcgtctgccc	gtg	None	0	hypothetical conserved protein	
**CcrRogue_gp165**	-	acaacgccGGAGGccctttc	atg	ggagg	8	hypothetical conserved protein	
**CcrRogue_gp166**	-	cctgatccccGAGaaaacgtc	atg	gag	8	putative HD-domain/PDEase-like protein	SSF109604
**CcrRogue_gp167**	-	cgaattcttGAGGgtcgcctg	atg	gagg	8	hypothetical conserved protein	
**CcrRogue_gp168**	-	acgccaagaAGGAttgatccc	atg	agga	8	putative DNA-binding domain protein	IPR016177, SSF54060
**CcrRogue_gp169**	-	cctccacgaagtAGGTccgcc	atg	aggt	5	hypothetical conserved protein	
**CcrRogue_gp170**	-	gctcgaaaAGGAaccgacctg	atg	agga	9	hypothetical conserved protein	
**CcrRogue_gp171**	-	tcgtcaacaaGGGGtccaagc	atg	gggg	7	hypothetical conserved protein	
**CcrRogue_gp172**	-	caagcAGGcggcgggcttgct	ttg	agg	13	hypothetical conserved protein	
**CcrRogue_gp173**	-	cctgttcaagtGGAccaaatc	gtg	gga	7	hypothetical conserved protein	
**CcrRogue_gp174**	-	ctctgcgtgaAGGgctagtcg	atg	agg	8	hypothetical conserved protein	
**CcrRogue_gp175**	-	gacaacggcagcgAGGAgcac	atg	agga	4	putative RtcB-like protein	IPR001233
**CcrRogue_gp176**	-	cgcagcatgctGGAGGacacc	ctg	ggagg	5	hypothetical novel protein	
**CcrRogue_gp177**	-	aacaagacgAGGAGtaacccc	ttg	aggag	7	putative band 7 lipoprotein	IPR001107
**CcrRogue_gp178**	-	cattctgaaAGGTcccacctc	gtg	aggt	8	hypothetical conserved protein	
**CcrRogue_gp179**	-	gcagcGGGGgctcgcgggctg	atg	gggg	11	hypothetical conserved protein	
**CcrRogue_gp180**	-	acgacaacaAGGAGacccgat	ttg	aggag	7	hypothetical conserved protein	
**CcrRogue_gp181**	-	gttcggctggtAGGAGGaccc	atg	aggagg	4	hypothetical conserved protein	
**CcrRogue_gp182**	-	acctgctgatGGAGacccgcc	gtg	ggag	7	hypothetical conserved protein	
**CcrRogue_gp183**	-	ccgccgagaAGGTgcaggctg	atg	aggt	8	hypothetical novel protein	
**CcrRogue_gp184**	-	ggaaGAGGccgagcaaagctg	atg	gagg	13	hypothetical novel protein	
**CcrRogue_gp185**	-	tcctagccgaaaAGGgtcgcc	atg	agg	6	hypothetical conserved protein	
**CcrRogue_gp186**	-	cgtcctctcgacgatcacgac	atg	None	0	hypothetical conserved protein	
**CcrRogue_gp187**	-	cacggatagaaAGGAGccaag	atg	aggag	5	hypothetical novel protein	
**CcrRogue_gp188**	-	cggtgggcgcgaacgcgttcc	atg	None	0	hypothetical conserved protein	
**CcrRogue_gp189**	-	cctgggcctGGAGaagcacct	gtg	ggag	8	hypothetical conserved protein	
**CcrRogue_gp190**	-	actggcgaAGGTgtcggcctg	atg	aggt	9	hypothetical conserved protein	
**CcrRogue_gp191**	-	caacgacagAGGAGacctctc	ttg	aggag	7	hypothetical conserved protein	
**CcrRogue_gp192**	-	caatcgtccgaGGGGcaagaa	atg	gggg	6	hypothetical conserved protein	
**CcrRogue_gp193**	-	gacgacaatctGGAGaccgcg	atg	ggag	6	putative winged-helix HTH DNA binding protein	IPR011991
**CcrRogue_gp194**	-	catgaacgtctcgtgcgggga	atg	None	0	hypothetical novel protein	
**CcrRogue_gp195**	-	cgacggcGGAGcctacatcct	atg	ggag	10	hypothetical novel protein	
**CcrRogue_gp196**	-	gaccatgccggtggtccgtcg	atg	None	0	hypothetical novel protein	
**CcrRogue_gp197**	-	acgacaacgaAGGAGGcccac	gtg	aggagg	5	hypothetical novel protein	
**CcrRogue_gp198**	-	ggacagtcgctGGGGcactgc	atg	gggg	6	hypothetical conserved protein	
**CcrRogue_gp199**	-	caacggtGAGaagttcctctg	atg	gag	11	hypothetical novel protein	
**CcrRogue_gp200**	-	ggaaagtcaaAGGTccgatcg	atg	aggt	7	hypothetical conserved protein	
**CcrRogue_gp201**	-	tcaagcgGGAcctgaacgtcg	atg	gga	11	hypothetical conserved protein	
**CcrRogue_gp202**	-	cgtgaccaagcaGAGGgcctg	atg	gagg	5	hypothetical conserved protein	
**CcrRogue_gp203**	-	tGGAGtgaagtccagatcgcg	gtg	ggag	16	putative acyl carrier protein	IPR009081, IPR006163, PTHR20863
**CcrRogue_gp204**	-	tcgagaacggcgAGGAcggct	atg	agga	5	putative HNH endonuclease	IPR002711
**CcrRogue_gp205**	-	cgtcctctactAGGAGacccc	atg	aggag	5	hypothetical conserved protein	
**CcrRogue_gp206**	-	cgtacctGGGGtatcaggccg	atg	gggg	10	hypothetical conserved protein	
**CcrRogue_gp207**	-	cggGGAGaagctgaagtgagc	atg	ggag	14	hypothetical conserved protein	
**CcrRogue_gp208**	-	ccgtgcctcGGAGaaggccct	ttg	ggag	8	hypothetical conserved protein	
**CcrRogue_gp209**	-	ccgcttcgAGGAGatcacggc	ttg	aggag	8	hypothetical conserved protein	
**CcrRogue_gp210**	-	cgacgacaacggaAGGAcccg	atg	agga	4	hypothetical conserved protein	
**CcrRogue_gp211**	-	tttcgccgaGGGGgaccgcac	gtg	gggg	7	hypothetical conserved protein	
**CcrRogue_gp212**	-	cttcgaaactagGGAGGgctc	atg	ggagg	4	hypothetical conserved protein	
**CcrRogue_gp213**	-	gcgcccggcgaAGGccactg	atg	agg	7	hypothetical conserved protein	
**CcrRogue_gp214**	-	acgacaacccaAGGAGGccccg	atg	aggagg	5	hypothetical conserved protein	
**CcrRogue_gp215**	-	atacgtGGAGagcaaggcggc	gtg	ggag	11	hypothetical conserved protein	
**CcrRogue_gp216**	-	actgcgGGAGtgtaactgacc	atg	ggag	11	hypothetical conserved protein	SSF56784
**CcrRogue_gp217**	-	cgcccaatttccGGAGtagac	atg	ggag	5	hypothetical novel protein	
**CcrRogue_gp218**	-	atgcagggcctGGGGgactag	gtg	gggg	5	hypothetical conserved protein	
**CcrRogue_gp219**	-	gctgatcaagtcGAGGccctg	atg	gagg	5	hypothetical conserved protein	
**CcrRogue_gp220**	-	gcctgccctacAGGAtcacct	atg	agga	6	hypothetical conserved protein	
**CcrRogue_gp221**	-	gatcgccgggttcgtcgccgc	gtg	None	0	hypothetical conserved protein	
**CcrRogue_gp222**	-	aaaagaAGGAGGaagccgcca	atg	aggagg	9	putative HTH domain DNA-binding protein	IPR010982, IPR001387
**CcrRogue_gp223**	-	aatcgcccAGGgcgccgcctg	atg	agg	10	hypothetical conserved protein	
**CcrRogue_gp224**	-	cgacgacatagAGGAGttccc	gtg	aggag	5	hypothetical conserved protein	
**CcrRogue_gp225**	-	ccaaacgaataGGGGaccgcc	ttg	gggg	6	hypothetical conserved protein	
**CcrRogue_gp226**	-	ccgcaagaAGGAccccacggc	atg	agga	9	hypothetical conserved protein	
**CcrRogue_gp227**	-	cggcaagatcaaGAGGgcctg	atg	gagg	5	putative Pol I DNA polymerase	IPR001098, IPR002298

**CcrRogue_gp228**	-	agaacAGGAGtagcgtacaag	gtg	aggag	11	putative GcrA-like cell cycle regulator	IPR011681
**CcrRogue_gp229**	-	cggcgaaaatcgAGGAGcgtc	atg	aggag	4	hypothetical conserved protein	
**CcrRogue_gp230**	+	cgacaacgaaAGGAGGcgctg	atg	aggagg	5	hypothetical conserved protein	
**CcrRogue_gp231**	+	cgcccgccAGGAcatggccgc	atg	agga	9	hypothetical conserved protein	
**CcrRogue_gp232**	+	cgacaagatcGAGGaaGAGGt	ttg	gagg	1	hypothetical conserved protein	
**CcrRogue_gp233**	+	ttctgccccAGGAGcttctcg	atg	aggag	7	hypothetical conserved protein	
**CcrRogue_gp234**	+	cattttcgactAGGAGGgacc	atg	aggagg	4	hypothetical conserved protein	
**CcrRogue_gp235**	+	catcgtcGAGGcgttcGAGGc	gtg	gagg	1	hypothetical conserved protein	
**CcrRogue_gp236**	+	tcctggagaagcAGGActgac	gtg	agga	5	hypothetical conserved protein	
**CcrRogue_gp237**	+	cgacaacgcaAGGAGcctgtc	ttg	aggag	6	hypothetical conserved protein	
**CcrRogue_gp238**	+	gagcctGGAGccggtggcctg	atg	ggag	11	hypothetical conserved protein	
**CcrRogue_gp239**	+	catgaaaaagagGGAGGgcta	atg	ggagg	4	hypothetical conserved protein	
**CcrRogue_gp240**	+	cccgccccAGGAGccgccgac	gtg	aggag	8	hypothetical conserved protein	
**CcrRogue_gp241**	+	tgacctcaagaAGGAaatcca	atg	agga	6	hypothetical conserved protein	
**CcrRogue_gp242**	+	gacgacaacgAGGAGGccccg	atg	aggagg	5	hypothetical conserved protein	
**CcrRogue_gp243**	+	cgccggaatgGGAGattcaag	atg	ggag	7	hypothetical conserved protein	
**CcrRogue_gp244**	+	cggcctggagcttGAGGcctg	atg	gagg	4	hypothetical conserved protein	
**CcrRogue_gp245**	+	acctggacgAGGAGaactgac	atg	aggag	7	hypothetical conserved protein	
**CcrRogue_gp246**	+	ggaagatgcGGAGccgaaggt	ttg	ggag	8	hypothetical conserved protein	
**CcrRogue_gp247**	+	caacgccctgGGAGccgcccg	atg	ggag	7	hypothetical conserved protein	
**CcrRogue_gp248**	+	cccgaccagcGAGGTcaagcc	atg	gaggt	6	hypothetical novel protein	
**CcrRogue_gp249**	+	acgacaacgaAGGAGGcttcc	gtg	aggagg	5	hypothetical novel protein	
**CcrRogue_gp250**	+	gctctcgtgggccaAGGtctg	atg	agg	4	hypothetical conserved protein	
**CcrRogue_gp251**	+	tggcccatcacGGAGGctgac	atg	ggagg	5	hypothetical novel protein	
**CcrRogue_gp252**	+	gcacgtGGAGGccaagctctg	atg	ggagg	10	hypothetical conserved protein	
**CcrRogue_gp253**	+	gatcatcccgccGAGGTtctg	atg	gaggt	4	hypothetical conserved protein	
**CcrRogue_gp254**	+	gacgacaacaaAGGAGGcccc	atg	aggagg	4	hypothetical conserved protein	
**CcrRogue_gp255**	+	ctacatcagGGAcatgcgccc	gtg	gga	9	hypothetical conserved protein	
**CcrRogue_gp256**	+	tcagcgtGGAGGTggctgctg	atg	ggaggt	8	hypothetical conserved protein	
**CcrRogue_gp257**	+	catggacgcgAGGAagggctg	atg	agga	7	hypothetical conserved protein	
**CcrRogue_gp258**	+	tgctgcgcgaAGGAGacgacc	ttg	aggag	6	hypothetical conserved protein	
**CcrRogue_gp259**	+	agccaaaGGGGttcGGGGcaa	atg	gggg	3	hypothetical conserved protein	
**CcrRogue_gp260**	+	cttcgtcGAGGcggtctccac	gtg	gagg	10	hypothetical conserved protein	
**CcrRogue_gp261**	+	aacacgtcAGGAacaccagcc	atg	agga	9	putative TAT signal protein	IPR006311; PRED-TAT
**CcrRogue_gp262**	+	agccaagaAGGcccAGGcctg	atg	agg	4	hypothetical conserved protein	
**CcrRogue_gp263**	+	acctccagaAGGAGcccacct	atg	aggag	7	hypothetical conserved protein	
**CcrRogue_gp264**	+	agcccggcggcGGGGcgagta	atg	gggg	6	hypothetical conserved protein	
**CcrRogue_gp265**	+	ccagcctgaagcgcttctgac	atg	None	0	hypothetical conserved protein	
**CcrRogue_gp266**	+	accgctcgacgggtGGAacgg	atg	gga	4	putative pleckstrin domain protein	IPR001849
**CcrRogue_gp267**	+	tgaggaagAGGAGcgctgctg	atg	aggag	8	putative Ser/Thr protein phosphatase	IPR004843, IPR006186, SSF56300, PTHR11668, G3DSA:3.60.21.10
**CcrRogue_gp268**	+	gctcaagatcacggtgtgacg	atg	None	0	hypothetical conserved protein	
**CcrRogue_gp269**	+	gacgacaacgAGGAGGgccgc	atg	aggagg	5	hypothetical novel protein	
**CcrRogue_gp270**	+	cgtcatgaAGGgccgcttcta	atg	agg	10	hypothetical novel protein	
**CcrRogue_gp271**	+	cggcgcggcgctgccgttcta	atg	None	0	hypothetical conserved protein	
**CcrRogue_gp272**	+	gacgacaacgAGGAGGccccc	atg	aggagg	5	hypothetical conserved protein	
**CcrRogue_gp273**	+	gtggcgggcGAGGcctaagcc	atg	gagg	8	hypothetical novel protein	
**CcrRogue_gp274**	+	ggcgctgacgaAGGgctggtg	atg	agg	7	hypothetical conserved protein	
**CcrRogue_gp275**	+	taatctggaGAGGgccttaag	ttg	gagg	8	hypothetical conserved protein	
**CcrRogue_gp276**	+	aagttctAGGAaAGGAagacc	atg	agga	5	hypothetical novel protein	
**CcrRogue_gp277**	+	acaacgccGGAGGccctttc	atg	ggagg	8	hypothetical novel protein	
**CcrRogue_gp278**	+	cgccgcctcGGGGtggttcga	atg	gggg	8	hypothetical novel protein	
**CcrRogue_gp279**	+	actactagGGAGGaccccgac	atg	ggagg	8	hypothetical novel protein	
**CcrRogue_gp280**	+	acgacaacgaAGGAGGccccg	atg	aggagg	5	hypothetical novel protein	
**CcrRogue_gp281**	+	cgaccgcatGGGGtacgccgc	atg	gggg	8	hypothetical novel protein	
**CcrRogue_gp282**	+	ctcccacgacggctacgatct	gtg	None	0	hypothetical conserved protein	
**CcrRogue_gp283**	+	gctgctggcgggcgtggacct	gtg	None	0	hypothetical novel protein	
**CcrRogue_gp284**	+	ccagatgaAGGAGcccgcctc	ttg	aggag	8	hypothetical novel protein	
**CcrRogue_gp285**	+	acctcagaAGGAGGcccagcc	ttg	aggagg	7	hypothetical novel protein	
**CcrRogue_gp286**	+	ctctcgtctAGGAGacccgtc	ttg	aggag	7	hypothetical conserved protein	
**CcrRogue_gp287**	+	ggcctacgcttcggtgagcgc	atg	None	0	hypothetical conserved protein	
**CcrRogue_gp288**	+	gttctgGGAGGaaggcaatac	gtg	ggagg	10	hypothetical conserved protein	
**CcrRogue_gp289**	+	cggacataatAGGAGGTccag	atg	aggaggt	4	hypothetical novel protein	
**CcrRogue_gp290**	+	ggcggtcgaacgcgcggcggg	gtg	None	0	hypothetical conserved protein	
**CcrRogue_gp291**	+	cttcgcctcGGGGtggttcgc	atg	gggg	8	hypothetical novel protein	
**CcrRogue_gp292**	+	cgactaccgctAGGAGGtccc	atg	aggagg	4	hypothetical conserved protein	
**CcrRogue_gp293**	+	cgacgacggAGGAGacctgac	atg	aggag	7	hypothetical novel protein	
**CcrRogue_gp294**	+	ctccGGAGGcccgacatggca	atg	ggagg	12	hypothetical novel protein	
**CcrRogue_gp295**	+	catgaAGGgcgtcgacctcgg	atg	agg	13	hypothetical conserved protein	
**CcrRogue_gp296**	+	cgacgacaacAGGAGGccctc	atg	aggagg	5	hypothetical conserved protein	
**CcrRogue_gp297**	+	ttgcggctcagAGGAttcgtt	atg	agga	6	hypothetical conserved protein	
**CcrRogue_gp298**	+	ccggcggctgacgggtgcatc	atg	None	0	hypothetical conserved protein	
**CcrRogue_gp299**	+	gacgacaacgAGGAGGccccc	atg	aggagg	5	hypothetical conserved protein	
**CcrRogue_gp300**	+	atcaaggtGGAGGTctaagcc	atg	ggaggt	7	hypothetical novel protein	
**CcrRogue_gp301**	+	tctggtGGAGGccagcctcgc	atg	ggagg	10	hypothetical novel protein	
**CcrRogue_gp302**	+	catcgagcagAGGAagcaatc	gtg	agga	7	hypothetical conserved protein	
**CcrRogue_gp303**	+	gggactgGGAGacgacgactg	atg	ggag	10	hypothetical conserved protein	
**CcrRogue_gp304**	+	acgctgcgaAGGAGccgccac	gtg	aggag	7	putative winged-helix HTH DNA binding protein	IPR011991

**CcrRogue_gp305**	+	catgctgaaagagaAGGccgc	atg	agg	4	hypothetical conserved protein	
**CcrRogue_gp306**	+	ccgcaagctGGAGGccgacct	gtg	ggagg	7	hypothetical conserved protein	
**CcrRogue_gp307**	+	ccgcgacacggcaAGGAacgc	atg	agga	4	hypothetical conserved protein	
**CcrRogue_gp308**	+	ccctgcacgcaaGGGGctgac	atg	gggg	5	hypothetical conserved protein	
**CcrRogue_gp309**	+	gcgctttgatcccgtgacctg	atg	None	0	hypothetical conserved protein	
**CcrRogue_gp310**	+	cctgcatcgGGAcgtcatctg	atg	gga	9	hypothetical conserved protein	
**CcrRogue_gp311**	+	cgacgtgccGAGGcggacctg	atg	gagg	8	hypothetical conserved protein	
**CcrRogue_gp312**	+	cttcgacggctGGAGGTcgtc	atg	ggaggt	4	hypothetical conserved protein	
**CcrRogue_gp313**	+	tctggagtatcGGGGcgtcca	gtg	gggg	6	putative HNH homing endonuclease	IPR002711
**CcrRogue_gp314**	+	cgacaacgaAGGAGaccccgc	atg	aggag	7	hypothetical novel protein	
**CcrRogue_gp315**	+	cgaagccgccgggcgcgccgc	atg	None	0	hypothetical novel protein	
**CcrRogue_gp316**	+	gatccgctatcatcggggctt	ttg	None	0	hypothetical conserved protein	
**CcrRogue_gp317**	+	tcgtcgacaacatctggacct	atg	None	0	hypothetical conserved protein	
**CcrRogue_gp318**	+	gtttctccAGGAGatgttcgc	ttg	aggag	8	hypothetical novel protein	
**CcrRogue_gp319**	+	cgactactttgggtccggccg	atg	None	0	hypothetical conserved protein	
**CcrRogue_gp320**	+	cgactaccggctgctgctcgc	atg	None	0	hypothetical novel protein	
**CcrRogue_gp321**	+	ctacacgttGAGGgcggtgcg	atg	gagg	8	hypothetical novel protein	
**CcrRogue_gp322**	+	actctctagtaAGGcttcatt	atg	agg	7	hypothetical novel protein	
**CcrRogue_gp323**	+	tacaagaGGGGaaatatacca	atg	gggg	10	hypothetical conserved protein	
**CcrRogue_gp324**	+	ttggtccctcgGGAGccgctc	atg	ggag	6	hypothetical conserved protein	
**CcrRogue_gp325**	+	gccatcgcctaGAGcaggacc	atg	gag	7	hypothetical conserved protein	
**CcrRogue_gp326**	+	cgacggcgtGGAcgtgaccga	atg	gga	9	hypothetical novel protein	
**CcrRogue_gp327**	+	tgacgacgctggcggtggtgc	atg	None	0	putative RNaseH-like domain protein	IPR012337
**CcrRogue_gp328**	+	cgaaccggcGGAGttcccggc	ttg	ggag	8	hypothetical conserved protein	
**CcrRogue_gp329**	+	aggtcacccAGGAaaaggtcg	atg	agga	8	hypothetical conserved protein	
**CcrRogue_gp330**	+	agcgcccGGAactctgatccc	atg	gga	11	Terminase small subunit	HHpred vs pdb70_18Aug12, hit 3zqp_A, prob 95.7% E=0.0062; position relative to terminase large subunit, see text
**CcrRogue_gp331**	+	cgcgctgaccactgatcgtct	atg	None	0	Terminase large subunit	IPR004921, IPR007868, IPR006141, IPR003587, IPR006517, SSF51294, G3DSA:2.170.16.10

Table S6. Predicted proteins, gene starts and annotations of *C. crescentus* phage CcrColossus. Upstream sequence is the 25 bp of DNA sequence upstream of the start codon, with the predicted Shine-Dalgarno motif capitalized. S-D is the Shine-Dalgarno motif associated with the gene start, and spacing is the interval (in bp) between the S-D and start codon. Evidence includes detected conserved domains or motifs, BlastP and HHpred hits, and experimental or other evidence as detailed in the text.

Protein name	Strand	Upstream Sequence	Start Codon	S-D	spacing	Predicted product	Evidence
**CcrColossus_gp001**	-	cgcgctctccaAGGTgaatcc	atg	aggt	6	hypothetical novel protein	
**CcrColossus_gp002**	-	tctttgacatcgtGAGGcatc	atg	gagg	4	hypothetical novel protein	
**CcrColossus_gp003**	+	tcccgcgccctGGAGtctcgc	atg	ggag	6	hypothetical conserved protein	
**CcrColossus_gp004**	+	gccttcaagtAGGAGtcgccc	gtg	aggag	6	hypothetical novel protein	
**CcrColossus_gp005**	+	tcccgccctGGAGatgaaccg	atg	ggag	8	hypothetical novel protein	
**CcrColossus_gp006**	+	ccgcgccctGGAGcaagtcac	atg	ggag	8	hypothetical novel protein	
**CcrColossus_gp007**	+	ccggctgatccgtGAGGggcg	atg	gagg	4	hypothetical novel protein	
**CcrColossus_gp008**	+	tcccgccctGGAGaacctgag	atg	ggag	8	hypothetical novel protein	
**CcrColossus_gp009**	+	gtcccgccGGAGaaaaacgac	atg	ggag	9	hypothetical novel protein	
**CcrColossus_gp010**	+	gcctctctGGAGcgcaagccc	atg	ggag	9	hypothetical novel protein	
**CcrColossus_gp011**	+	catcccctGGAGcgcaagccc	atg	ggag	9	hypothetical novel protein	
**CcrColossus_gp012**	+	accctcgtttgggttgtcgag	ttg	None	0	hypothetical conserved protein	
**CcrColossus_gp013**	+	ccctcaggacaAGGAGtccgc	atg	aggag	5	hypothetical novel protein	
**CcrColossus_gp014**	+	cccaaactcacgGGAGGccac	atg	ggagg	4	hypothetical conserved protein	
**CcrColossus_gp015**	+	ccgcgcccctGGAGaacgcac	atg	ggag	7	hypothetical conserved protein	
**CcrColossus_gp016**	+	aagccctggaaAGGccctgaa	atg	agg	7	hypothetical novel protein	
**CcrColossus_gp017**	+	acccctcgGGAGacacccgcc	atg	ggag	9	hypothetical conserved protein	
**CcrColossus_gp018**	+	tccggcaAGGcggcctagcct	atg	agg	11	hypothetical novel protein	
**CcrColossus_gp019**	+	atccgcaAGGAagcctaaccg	atg	agga	10	hypothetical novel protein	
**CcrColossus_gp020**	+	tcgactcgGGAGccctagccc	atg	ggag	9	hypothetical novel protein	
**CcrColossus_gp021**	+	cctaaccGGAGGaacccgccc	atg	ggagg	9	hypothetical novel protein	
**CcrColossus_gp022**	+	cagtagaaacaAGGAGttaac	ttg	aggag	5	hypothetical novel protein	
**CcrColossus_gp023**	+	ctttctgttGGAGcgcagaca	atg	ggag	8	hypothetical novel protein	
**CcrColossus_gp024**	+	ccgcgccctGGAGaaaatcgg	atg	ggag	8	hypothetical novel protein	
**CcrColossus_gp025**	+	cgccctaccGGAGcgcctaac	atg	ggag	8	hypothetical novel protein	
**CcrColossus_gp026**	+	tcacgcctAGGAGGgcctgac	atg	aggagg	7	hypothetical novel protein	
**CcrColossus_gp027**	+	tcttgttaagaaatAGGccct	atg	agg	4	hypothetical novel protein	
**CcrColossus_gp028**	+	cccaagccctGGAGtccgctc	atg	ggag	7	hypothetical novel protein	
**CcrColossus_gp029**	+	ccctagctGGAGcgaaagccc	atg	ggag	9	hypothetical novel protein	
**CcrColossus_gp030**	+	acaacctcgaaAGGAcaaccc	ttg	agga	6	hypothetical novel protein	
**CcrColossus_gp031**	+	aatggcctctaGAGtgactac	atg	gag	7	hypothetical novel protein	
**CcrColossus_gp032**	+	ctaggactGGAGtccttcgca	atg	ggag	9	hypothetical novel protein	
**CcrColossus_gp033**	+	cttccccgctaGAGtgtgcgt	atg	gag	7	hypothetical novel protein	
**CcrColossus_gp034**	+	aaggccctAGGAGacaaagcg	atg	aggag	8	hypothetical novel protein	
**CcrColossus_gp035**	+	cctcacctcGGAGcccctgcc	atg	ggag	8	hypothetical conserved protein	
**CcrColossus_gp036**	-	tccatcaatcAGGAGattcag	gtg	aggag	6	hypothetical conserved protein	
**CcrColossus_gp037**	-	tctcctgccGGActtcctgtc	atg	gga	9	putative transglutaminase-like cysteine peptidase	IPR010319, PS51257
**CcrColossus_gp038**	+	cgcccacttttcaaaagaac	atg	None	0	putative portal protein	HHpred vs pdb70_18Aug12, hit 2jes_A, prob 99.6% E=9.1E-15; positional evidence, see text
**CcrColossus_gp039**	+	gccgcaaaatGGAGcgagcta	atg	ggag	7	hypothetical novel protein	
**CcrColossus_gp040**	+	tccggtgaAGGAcgaagcctg	atg	agga	9	hypothetical novel protein	
**CcrColossus_gp041**	+	ctccaagcctGGAaagaagaa	gtg	gga	8	hypothetical novel protein	
**CcrColossus_gp042**	+	ttcagtacccagcgtatcgcc	atg	None	0	hypothetical conserved protein	
**CcrColossus_gp043**	+	tccgaagaAGGActagccccg	atg	agga	9	hypothetical novel protein	
**CcrColossus_gp044**	+	tgaaggctaaAGGAGcttccc	gtg	aggag	6	hypothetical novel protein	
**CcrColossus_gp045**	+	gctgcgtcttcggctaagccg	atg	None	0	hypothetical novel protein	
**CcrColossus_gp046**	+	cgagtcctgctGGGGtgattg	atg	gggg	6	hypothetical novel protein	
**CcrColossus_gp047**	+	gttcctgatGGGGctcgcact	atg	gggg	8	hypothetical novel protein	
**CcrColossus_gp048**	+	ctgcccatttggctgtgacga	atg	None	0	hypothetical novel protein	PD968187
**CcrColossus_gp049**	+	cttcagtccatGGAGGaataa	atg	ggagg	5	putative ATPase domain protein	SSF52540, G3DSA:3.40.50.300
**CcrColossus_gp050**	+	ccgccttcagaAGGgttgatc	atg	agg	7	hypothetical conserved protein	
**CcrColossus_gp051**	+	agtcgcagagGGAGcctaatc	atg	ggag	7	hypothetical novel protein	
**CcrColossus_gp052**	+	ctcctgcacGAGGTttaagcc	atg	gaggt	7	hypothetical conserved protein	
**CcrColossus_gp053**	+	cctccgcatcccggtcggctg	atg	None	0	hypothetical novel protein	
**CcrColossus_gp054**	+	attcgaaagaactggtcagcc	atg	None	0	hypothetical novel protein	
**CcrColossus_gp055**	+	cctcgccgacgGGAGcatctg	atg	ggag	6	hypothetical novel protein	
**CcrColossus_gp056**	+	ggtcggccggtAGGAGGagtc	atg	aggagg	4	hypothetical conserved protein	
**CcrColossus_gp057**	+	attgcctaccgctcAGGaatt	ttg	agg	4	putative Ntn hydrolase domain proetin	SSF56235
**CcrColossus_gp058**	+	caccctgaagttgcGAGatta	atg	gag	4	hypothetical novel protein	
**CcrColossus_gp059**	+	tctcttcatgtcGGGGgtcat	gtg	gggg	4	hypothetical conserved protein	
**CcrColossus_gp060**	+	ggaaatgaaaaAGGTcgcctg	atg	aggt	6	hypothetical conserved protein	
**CcrColossus_gp061**	+	cgcccccgacGGAGcgaaggc	gtg	ggag	7	hypothetical conserved protein	
**CcrColossus_gp062**	+	cctccgcccGGAcgccgctgc	atg	gga	9	hypothetical novel protein	
**CcrColossus_gp063**	+	ggtaccggccgAGGAtaagac	atg	agga	6	hypothetical novel protein	
**CcrColossus_gp064**	+	ccgccgaagAGGAaaagtagg	atg	agga	8	hypothetical novel protein	
**CcrColossus_gp065**	+	taacgcttcccggctggtcct	gtg	None	0	hypothetical novel protein	
**CcrColossus_gp066**	+	tctgcacctGGAGcaagtcca	ttg	ggag	8	hypothetical novel protein	
**CcrColossus_gp067**	+	tctggtgGGAtgggccgacta	atg	gga	11	hypothetical conserved protein	
**CcrColossus_gp068**	+	gcacgacccGGAcaagcgctg	atg	gga	9	hypothetical novel protein	
**CcrColossus_gp069**	+	tcgccaaatGGAGttgccgct	gtg	ggag	8	hypothetical conserved protein	
**CcrColossus_gp070**	+	cttcgacgaagaAGGcctctg	atg	agg	6	putative DHH phosphoesterase protein	SSF64182
**CcrColossus_gp071**	+	gatcggctgGGAGGggcgctc	gtg	ggagg	7	hypothetical novel protein	
**CcrColossus_gp072**	+	atGGAcaacttcGGAcaagcg	ctg	gga	6	hypothetical novel protein	
**CcrColossus_gp073**	+	agcgctgaAGGAGccctcagc	atg	aggag	8	hypothetical conserved protein	

**CcrColossus_gp074**	+	caaccgacAGGcggctgcggc	gtg	agg	10	hypothetical novel protein	
**CcrColossus_gp075**	+	acattatGAGatcGAGcccgc	ttg	gag	5	hypothetical novel protein	
**CcrColossus_gp076**	+	aggactcaccagtGGAGtcct	atg	ggag	4	hypothetical novel protein	
**CcrColossus_gp077**	+	cctgcGAGGgacttcgttctg	atg	gagg	12	hypothetical conserved protein	
**CcrColossus_gp078**	+	ccgcgacGAGtaccaccgcta	atg	gag	11	hypothetical conserved protein	
**CcrColossus_gp079**	+	tccacacgctgctgacaccca	atg	None	0	putative HNH endonuclease	IPR002711, G3DSA:3.90.10.10
**CcrColossus_gp080**	+	gccggctctccccAGGgcttg	ttg	agg	5	hypothetical conserved protein	
**CcrColossus_gp081**	+	accaactaagGGAaagctcta	atg	gga	8	putative major capsid protein	experimental evidence, see text
**CcrColossus_gp082**	+	cttttctaAGGgcatctgaag	atg	agg	10	putative minor capsid protein	experimental evidence, see text
**CcrColossus_gp083**	-	atagttaaccGGGGttccctc	atg	gggg	7	hypothetical conserved protein	
**CcrColossus_gp084**	+	ttcctccgccgcAGGAaccac	atg	agga	5	hypothetical novel protein	
**CcrColossus_gp085**	-	ggcagttcggaAGGTccgcgt	atg	aggt	6	hypothetical novel protein	
**CcrColossus_gp086**	+	cccctcttggcGGAattttcg	gtg	gga	7	hypothetical conserved protein	
**CcrColossus_gp087**	+	ccGGAGatcgaaggcggcacc	atg	ggag	15	hypothetical conserved protein	
**CcrColossus_gp088**	+	gtacagcaactAGGTccaacg	atg	aggt	6	hypothetical conserved protein	
**CcrColossus_gp089**	+	cactctccttctcgaactaag	atg	None	0	hypothetical conserved protein	
**CcrColossus_gp090**	+	actttctaaAGGgcaaagccg	atg	agg	9	hypothetical conserved protein	
**CcrColossus_gp091**	+	tgatcgaagcgcccctgactc	gtg	None	0	putative lectin-like domain protein	IPR013320, IPR008985
**CcrColossus_gp092**	+	ctacgtgctggcgaacctcca	atg	None	0	hypothetical conserved protein	
**CcrColossus_gp093**	+	ctgtttcgcttcAGGTgaact	atg	aggt	5	hypothetical conserved protein	
**CcrColossus_gp094**	+	cggttttcttgacactgtagg	ttg	None	0	hypothetical novel protein	
**CcrColossus_gp095**	+	gtcttcggacgggaAGGccat	atg	agg	4	hypothetical novel protein	
**CcrColossus_gp096**	+	caccgccaAGGTttcgctgaa	atg	aggt	9	hypothetical conserved protein	
**CcrColossus_gp097**	+	gatcgtccAGGAGGagccagc	atg	aggagg	7	hypothetical novel protein	
**CcrColossus_gp098**	+	cgcgagcaagGGAGccgcaac	atg	ggag	7	hypothetical novel protein	
**CcrColossus_gp099**	-	catcaaaccaAGGAataccat	atg	agga	7	hypothetical novel protein	
**CcrColossus_gp100**	+	tggcgacgctaaactgcgccc	atg	None	0	putative FkbM-like methyltransferase	IPR006342, G3DSA:3.40.50.150
**CcrColossus_gp101**	+	cgcaataacgAGGAGacttac	atg	aggag	6	hypothetical conserved protein	
**CcrColossus_gp102**	+	ccaccgcctcccGGAGcttcc	atg	ggag	5	putative protein metallo-phosphoesterase	IPR004843, SSF56300, G3DSA:3.60.21.10
**CcrColossus_gp103**	+	tctggtggtGGAGatttaacg	atg	ggag	8	hypothetical conserved protein	
**CcrColossus_gp104**	+	cctccAGGAGcagcacgccta	atg	aggag	11	putative peptide formylmethionine deformylase	IPR000181
**CcrColossus_gp105**	+	aactctctgcctGGAGtccac	atg	ggag	5	hypothetical conserved protein	
**CcrColossus_gp106**	+	ctaccgtcGGGGacgaaggct	gtg	gggg	9	hypothetical novel protein	
**CcrColossus_gp107**	+	gatcgcgtgcgGGAGatcgag	atg	ggag	6	hypothetical novel protein	
**CcrColossus_gp108**	+	acgcgccctcGGAGGgacac	atg	ggagg	6	hypothetical novel protein	
**CcrColossus_gp109**	+	tacgccgcaagGGAGcgacgt	ttg	ggag	6	putative PhoH-like protein	IPR003714, SSF52540, G3DSA:3.40.50.300
**CcrColossus_gp110**	+	agtcatAGGgtagaacggtcg	atg	agg	12	hypothetical conserved protein	
**CcrColossus_gp111**	+	aagatcgtccggtcgtaatcc	atg	None	0	hypothetical conserved protein	
**CcrColossus_gp112**	+	tccagatgcgtgcggcctaac	gtg	None	0	hypothetical conserved protein	
**CcrColossus_gp113**	+	cgaagctatggcAGGTgtcct	gtg	aggt	5	hypothetical novel protein	
**CcrColossus_gp114**	+	gctctctgAGGAGacctgacc	gtg	aggag	8	hypothetical conserved protein	
**CcrColossus_gp115**	+	tgcaacttttggtgggcagaa	atg	None	0	putative major tail tube protein	experimental evidence, see text
**CcrColossus_gp116**	+	ccctgacaAGGTaacccgaaa	atg	aggt	9	putative pre-tape measure chaperone protein	HHpred vs pdb70_18Aug12, hit 2ob9_A, prob 60.2% E=27; positional evidence, see text
**CcrColossus_gp117**	+	ccctgacaAGGTaacccgaaa	atg	aggt	9	putative pre-tape measure chaperone protein, frameshifted version	positional evidence, see text; slippery sequence AAAAAAC
**CcrColossus_gp118**	+	cttcgcggcgctGAGGgatag	atg	gagg	5	putative tail tape measure protein	IPR008258, IPR013491, SSF53955, G3DSA:1.10.530.10; experimental evidence, see text
**CcrColossus_gp119**	+	cacaacGAGttaacccccagc	atg	gag	12	DUF2460 protein	IPR011740
**CcrColossus_gp120**	+	tgattGGGGgaacctgttcta	atg	gggg	11	putative tail protein	IPR019228, IPR018964, IPR011928; experimental evidence, see text
**CcrColossus_gp121**	+	accGGAcgctccgacctcgta	atg	gga	15	putative NlpC/P60 family cell wall peptidase	IPR000064, SSF54001, G3DSA:3.90.1720.10
**CcrColossus_gp122**	+	ttccctggcgtGGAGGactaa	atg	ggagg	5	putative tail protein	experimental evidence, see text
**CcrColossus_gp123**	+	ccccgtacaccGAGttctaga	atg	gag	7	putative tail protein	experimental evidence, see text
**CcrColossus_gp124**	+	gagtcacgtttcggtccaaaa	atg	None	0	hypothetical conserved protein	
**CcrColossus_gp125**	+	tcaAGGgcgtctacttctaaa	atg	agg	15	putative tail protein	IPR000421, IPR008979, G3DSA:2.60.120.260; experimental evidence, see text
**CcrColossus_gp126**	+	ttattgggcacaGAGcttcta	atg	gag	6	putative alpha-2,3-sialyltransferase-like protein	IPR009251
**CcrColossus_gp127**	+	tcttcgccttaagacctaggg	atg	None	0	hypothetical novel protein	
**CcrColossus_gp128**	+	ggtgcccGAGGTggcaagatc	atg	gaggt	9	hypothetical conserved protein	
**CcrColossus_gp129**	+	gcctgaatttGGAcgccttgc	atg	gga	8	putative nucleotide-diphospho-sugar transferase	SSF53448
**CcrColossus_gp130**	+	tGGAttGGAcccgcgctctcc	atg	gga	12	hypothetical conserved protein	
**CcrColossus_gp131**	+	gcgatcGGAGacgcatgggca	atg	ggag	11	putative family 9 glycosyl transferase	IPR002201, SSF53756, G3DSA:3.40.50.2000
**CcrColossus_gp132**	+	gtcggcaattGGAGaaggtcc	atg	ggag	7	hypothetical conserved protein	
**CcrColossus_gp133**	+	tcaccgaaAGGgcattcatca	atg	agg	10	putative RNA polymerase alpha subunit C-terminal domain protein	IPR011260, G3DSA:1.10.150.20
**CcrColossus_gp134**	+	atcttgctGAGcctctctgac	atg	gag	10	putative endolysin	IPR002196, SSF53955, G3DSA:1.10.530.40
**CcrColossus_gp135**	+	ggcttGGAGtttcaagggcta	atg	ggag	12	putative holin protein	predicted 4 TMDs, see text
**CcrColossus_gp136**	+	caaattcAGGTgaccgactag	atg	aggt	10	putative inner membrane spanin component	N-terminal TMD, outer membrane spanin component embedded in +1 frame, see text
**CcrColossus_gp137**	+	ctgaagtGGAcacccttctcg	atg	gga	11	putative outer membrane spanin component	lipoprotein embedded in inner membrane spanin component, see text
**CcrColossus_gp138**	+	tcggcaagaAGGAatagcgcc	atg	agga	8	hypothetical novel protein	
**CcrColossus_gp139**	-	agtagAGGAGccgacccgacc	atg	aggag	11	putative TROVE-like domain protein	IPR008858, SSF140864, SSF53300
**CcrColossus_gp140**	-	ggcaccgacaAGGAcgaaggc	atg	agga	7	putative HTH domain DNA-binding protein	IPR001387, IPR010982, G3DSA:1.10.260.40
**CcrColossus_gp141**	-	acctacctgaAGGActaaccc	atg	agga	7	putative dUTP pyrophosphatase	IPR008181, IPR008180, SSF51283, PTHR11241, G3DSA:2.70.40.10
**CcrColossus_gp142**	-	cctctttttcGAGcgccttta	atg	gag	8	putative ribonucleoside diphosphate reductase beta subunit	IPR012348, IPR012335, IPR000358, IPR002109, IPR011767, IPR009078, IPR012336, PTHR23409:SF5,
**CcrColossus_gp143**	-	ccttttttcttcGGAaccgtac	atg	gga	7	putative ribonucleoside diphosphate reductase alpha subunit	IPR000788, IPR013509, IPR003587, IPR006141, IPR008926, SSF51998, SSF51294, G3DSA:3.20.70.20, G3DSA:2.170.16.10
**CcrColossus_gp144**	-	tagtcagtaGAGcgtacgcgt	atg	gag	9	hypothetical conserved protein	
**CcrColossus_gp145**	-	ccgcctcGGAGGaataaggtc	atg	ggagg	9	hypothetical conserved protein	
**CcrColossus_gp146**	-	caccaccgcctaAGGAccacc	atg	agga	5	hypothetical novel protein	
**CcrColossus_gp147**	-	tggcaatGAGttcgttcccaa	atg	gag	11	hypothetical conserved protein	
**CcrColossus_gp148**	-	cccccaacaaaaGAGcgcact	atg	gag	6	hypothetical conserved protein	
**CcrColossus_gp149**	-	tccgaacGAGcgcctgtaacc	gtg	gag	11	hypothetical novel protein	
**CcrColossus_gp150**	-	gaaccagtacgaaagccgcgc	atg	None	0	putative thymidylate synthase	IPR003669

**CcrColossus_gp151**	-	ccgacgcctAGGAagtcgccc	atg	agga	8	hypothetical novel protein	
**CcrColossus_gp152**	-	gtcctggtctgAGGAaacacc	atg	agga	6	putative dNMP kinase	HHpred vs pdb70_18Aug12, hit 3ch4_B, prob 99.9% E=8.5E-25; SSF52540, G3DSA:3.40.50.300
**CcrColossus_gp153**	-	cAGGgcgacgcggttcgcact	gtg	agg	17	putative RecD-like helicase	HHpred vs pdb70_18Aug12, hit 3e1s_A, prob 100% E=1.2E-44; IPR000606, SSF52540, G3DSA:3.40.50.300
**CcrColossus_gp154**	-	gacttagaagtagaaaaccag	atg	None	0	hypothetical conserved protein	
**CcrColossus_gp155**	-	cccgcctAGGAGtcacctttc	atg	aggag	9	hypothetical conserved protein	
**CcrColossus_gp156**	-	gccgaAGGgtggtcgttaatc	atg	agg	13	putative exoribonuclease, Pol III-like	IPR013520, IPR006055, IPR012337, G3DSA:3.30.420.10
**CcrColossus_gp157**	-	agggcctcctccGAGaacgac	atg	gag	6	hypothetical novel protein	
**CcrColossus_gp158**	-	ccttcatGAGGctcgcgccta	atg	gagg	10	putative Pol I DNA polymerase, T7-like	IPR001098, IPR012337, SSF56672, PTHR10133, G3DSA:3.30.420.10, G3DSA:1.20.1060.10, G3DSA:3.30.70.370, G3DSA:1.10.150.20; see text
**CcrColossus_gp159**	-	ccgttaaccataccggccccg	atg	None	0	putative DNA cytosine methyltransferase	IPR001525, IPR018117, SSF53335, PTHR10629:SF11, G3DSA:3.40.50.150, G3DSA:3.90.120.10
**CcrColossus_gp160**	-	gaacaacGGAaagtggcgcct	gtg	gga	11	hypothetical conserved protein	
**CcrColossus_gp161**	-	acgccaaggctGAGatgttct	atg	gag	7	putative T5 A1-like protein	blastP to T5 A1 (AAU05157), E=6.5E-90, 34.9% Dice identity; HHpred vs pdb70_18Aug12, hit 1g2h_A, prob 95.7% E=0.0072
**CcrColossus_gp162**	-	aacggtcaagcctGGAGagaa	atg	ggag	4	putative DNA methylase	IPR002052
**CcrColossus_gp163**	-	cctgcctcAGGcggcggctcc	atg	agg	10	hypothetical novel protein	
**CcrColossus_gp164**	-	gctggccGAGacaccgtagaa	atg	gag	11	hypothetical novel protein	
**CcrColossus_gp165**	-	tcgaAGGTccaagcctgacgc	atg	aggt	13	putative DNA helicase	IPR000330, IPR014001, IPR001650, IPR014021, SSF52540, PTHR10799, G3DSA:3.40.50.300
**CcrColossus_gp166**	-	ttccagtAGGAGtcacgtttc	gtg	aggag	9	hypothetical conserved protein	
**CcrColossus_gp167**	-	tgggaaaaccccgAGGAccct	atg	agga	4	putative RNA ligase	IPR021122, IPR012646, SSF56091
**CcrColossus_gp168**	-	catgcggaAGGAagaacaata	atg	agga	9	hypothetical novel protein	
**CcrColossus_gp169**	-	ggcggtGGAGatcacccgctg	atg	ggag	11	conserved T4 30.3-like protein	IPR012596, SSF143990
**CcrColossus_gp170**	-	agacctgGGActcgacttctg	atg	gga	11	hypothetical novel protein	
**CcrColossus_gp171**	-	cgatgccctAGGAGtcggcta	atg	aggag	7	hypothetical novel protein	
**CcrColossus_gp172**	-	cagactgacgagAGGAGccct	atg	aggag	4	putative nucleotidyltransferase protein	IPR018775
**CcrColossus_gp173**	-	caagccacGGAagcggcgcaa	atg	gga	10	hypothetical novel protein	
**CcrColossus_gp174**	-	gcacgcGGAGaagaaggcctc	atg	ggag	11	hypothetical novel protein	
**CcrColossus_gp175**	-	cgcacgctgtAGGAGcccgag	atg	aggag	6	hypothetical novel protein	
**CcrColossus_gp176**	-	gttcgacaAGGAaacgaagca	atg	agga	9	hypothetical novel protein	
**CcrColossus_gp177**	-	caaggccgacgAGGAtaagtg	atg	agga	6	hypothetical conserved protein	
**CcrColossus_gp178**	-	caagtccaAGGcctaacgacc	atg	agg	10	putative rIIb-like protein	PD130832
**CcrColossus_gp179**	-	caagctaaccgggcggggcgt	atg	None	0	putative rIIa-like protein	IPR003594
**CcrColossus_gp180**	+	tgtctggtatgcgAGGAagaa	ttg	agga	4	hypothetical conserved protein	
**CcrColossus_gp181**	+	cggcgactcgcgggtgctgaa	ttg	None	0	hypothetical conserved protein	
**CcrColossus_gp182**	+	acgataacgaAGGAGGccccg	atg	aggagg	5	putative tyrosine recombinase	IPR013762, IPR002104, IPR011010
**CcrColossus_gp183**	+	ttcgtgaacGGAtGGAcgagg	gtg	gga	5	hypothetical conserved protein	
**CcrColossus_gp184**	-	ccgcaAGGcgggccgtccctg	atg	agg	13	hypothetical novel protein	
**CcrColossus_gp185**	-	acctttttGAGGcaaattggaa	atg	gagg	10	hypothetical novel protein	
**CcrColossus_gp186**	+	atgagtattacAGGAGtttcc	atg	aggag	5	hypothetical conserved protein	
**CcrColossus_gp187**	+	gtaatgcGGAGagtgcatgaa	atg	ggag	10	hypothetical conserved protein	
**CcrColossus_gp188**	+	gcgcgctGGAGcgacgctcgc	gtg	ggag	10	hypothetical conserved protein	
**CcrColossus_gp189**	-	ggctgcctagaagAGGAtatc	atg	agga	4	putative YqeY-like protein	IPR019004
**CcrColossus_gp190**	-	gcacGGGGaagagcgcgtact	gtg	gggg	13	hypothetical conserved protein	G3DSA:1.10.340.30
**CcrColossus_gp191**	-	ggccgcaataGGGGaaacgac	atg	gggg	7	putative DNA ligase	IPR012340, IPR012310, IPR016027, SSF56091
**CcrColossus_gp192**	-	cttctacggtGGAGGccgccg	atg	ggagg	6	hypothetical conserved protein	
**CcrColossus_gp193**	-	aacccctctGGAGcgtttttg	atg	ggag	8	hypothetical conserved protein	
**CcrColossus_gp194**	-	cgtctggcgcccGGAGGcgtg	atg	ggagg	4	hypothetical novel protein	
**CcrColossus_gp195**	-	tgGGAtcatagcctcccGGAg	atg	gga	1	DUF1643 protein	IPR012441
**CcrColossus_gp196**	-	tggtccagcAGGcgaagatga	atg	agg	9	hypothetical novel protein	
**CcrColossus_gp197**	-	acaAGGAGaagggcctggacg	ttg	aggag	13	hypothetical novel protein	
**CcrColossus_gp198**	-	ctgggactaGAGagatcgcag	atg	gag	7	hypothetical conserved protein	
**CcrColossus_gp199**	-	ccactacacgccggtgaagcc	gtg	None	0	hypothetical novel protein	
**CcrColossus_gp200**	-	ctcctcgttaagctggctgct	gtg	None	0	hypothetical novel protein	
**CcrColossus_gp201**	-	ctaagcctGGAGGcggcgatc	atg	ggagg	8	hypothetical novel protein	
**CcrColossus_gp202**	-	tcaactgtaagcgAGGActcc	atg	agga	4	hypothetical novel protein	
**CcrColossus_gp203**	-	gccagcttctaGGGGtctccc	atg	gggg	6	putative nicotinate phosphoribosyltransferase	IPR015977, IPR002638, PTHR11098
**CcrColossus_gp204**	-	atgccgaatcacGGAcgaatg	atg	gga	6	putative NUDIX hydrolase domain protein	IPR014729, IPR000086, IPR004820, IPR015797, IPR020084, SSF52374, PS51462, PTHR22769,
**CcrColossus_gp205**	-	ccaaaatggcGAGGccgagag	ttg	gagg	7	hypothetical conserved protein	
**CcrColossus_gp206**	-	atgatagcggccttAGGtcg	atg	agg	4	hypothetical novel protein	
**CcrColossus_gp207**	-	tctacaAGGAtaaagagcacc	atg	agga	11	hypothetical novel protein	
**CcrColossus_gp208**	-	ctggaagaAGGcctagttcgg	gtg	agg	10	hypothetical novel protein	
**CcrColossus_gp209**	-	aagccttaaatcGAGaccaag	atg	gag	6	hypothetical novel protein	
**CcrColossus_gp210**	-	gcctcccctGAGtaaccgcag	atg	gag	9	hypothetical novel protein	
**CcrColossus_gp211**	-	tcaggtcacaacGGAGttccc	atg	ggag	5	hypothetical novel protein	
**CcrColossus_gp212**	-	ggcgatgcgaaAGGcgctctg	atg	agg	7	putative HD-domain/PDEase-like protein	SSF109604
**CcrColossus_gp213**	-	tggcggcggtGGAGGTgactg	atg	ggaggt	5	putative protein metallo-phosphoesterase	IPR004843, SSF56300, G3DSA:3.60.21.10
**CcrColossus_gp214**	-	gatctagcccaAGGAGaacca	atg	aggag	5	hypothetical novel protein	
**CcrColossus_gp215**	-	ccgtttgtGGGGgacgaagta	atg	gggg	8	hypothetical conserved protein	
**CcrColossus_gp216**	-	tgactggcatAGGAGagaacg	gtg	aggag	6	putative RtcB-like protein	IPR001233
**CcrColossus_gp217**	-	tgccatgtctaAGGcttgaag	atg	agg	7	hypothetical novel protein	
**CcrColossus_gp218**	-	gacGGGGatgaaccccgtccg	ctg	gggg	14	hypothetical novel protein	
**CcrColossus_gp219**	-	tgacgaaaAGGAGaggatgtc	gtg	aggag	8	putative TerD-like bacterial stress protein	IPR003325, G3DSA:2.60.60.30
**CcrColossus_gp220**	-	caagcatAGGAGttcaccttc	gtg	aggag	9	DUF475 protein	IPR007427
**CcrColossus_gp221**	-	aacatttccaggGGAGaaccc	atg	ggag	5	putative von Willebrand A domain protein	IPR002035, SSF53300
**CcrColossus_gp222**	-	aaggcttaAGGAGcctcatcg	atg	aggag	8	putative TelA-like resistance protein	IPR008863
**CcrColossus_gp223**	-	cgattgacacAGGAGacccaa	ttg	aggag	6	hypothetical novel protein	
**CcrColossus_gp224**	-	tcGGAGGcggcctgttcccac	atg	ggagg	14	hypothetical novel protein	
**CcrColossus_gp225**	+	tgttcgcggcactctatctcc	atg	None	0	hypothetical conserved protein	
**CcrColossus_gp226**	+	acgacaacgccAGGAGGcacc	atg	aggagg	4	hypothetical conserved protein	
**CcrColossus_gp227**	+	ttcaccggGGAGaactaaatc	atg	ggag	9	hypothetical novel protein	

**CcrColossus_gp228**	+	catcgagttcGAGGgcatctg	atg	gagg	7	hypothetical conserved protein	
**CcrColossus_gp229**	+	catagcagaAGGAcccactcc	gtg	agga	8	putative oxoglutarate oxygenase domain protein	IPR005123, SSF51197, PS51471, G3DSA:2.60.120.590
**CcrColossus_gp230**	+	aacctacaGGGGactcattca	atg	gggg	9	hypothetical novel protein	
**CcrColossus_gp231**	+	gctggcttttcctgaagtagg	atg	None	0	hypothetical novel protein	
**CcrColossus_gp232**	+	ccactagctgaAGGAGtcctc	atg	aggag	5	hypothetical novel protein	
**CcrColossus_gp233**	+	gacgatcagAGGAGactagac	atg	aggag	7	hypothetical conserved protein	
**CcrColossus_gp234**	+	tactgtaAGGAGaacgcgtac	atg	aggag	9	hypothetical novel protein	
**CcrColossus_gp235**	+	cctcacagccccGAGtgattc	atg	gag	6	putative AAA+ ATPase and mitochondrial BCS1 domain protein	IPR014851, IPR003959, IPR003593, IPR003960, SSF52540, PTHR23070, G3DSA:3.40.50.300
**CcrColossus_gp236**	+	aactggcaagcGGAaccattc	atg	gga	7	hypothetical novel protein	
**CcrColossus_gp237**	+	ccctaagcccaaaGAGGcctg	atg	gagg	4	UPF0114 protein	IPR005134
**CcrColossus_gp238**	+	ctctgcggaAGGAcactaaac	atg	agga	8	hypothetical conserved protein	
**CcrColossus_gp239**	+	cgggggcgacGGAGGctgatc	atg	ggagg	6	hypothetical novel protein	
**CcrColossus_gp240**	+	cgctacagtctgggccagcat	atg	None	0	hypothetical conserved protein	
**CcrColossus_gp241**	+	cgaaaggGGAGtcctcaccac	atg	ggag	10	putative lipoprotein	LipoP
**CcrColossus_gp242**	+	ccgcagcagtaGGGGgcgaac	ttg	gggg	5	putative CsgG-like curli assembly protein	IPR005534
**CcrColossus_gp243**	+	aaacaaacgacgaAGGAccct	atg	agga	4	hypothetical novel protein	
**CcrColossus_gp244**	+	tctgatctgacAGGAGacctc	gtg	aggag	5	putative ATPase domain protein	SSF52540, G3DSA:3.40.50.300
**CcrColossus_gp245**	+	gaaactcatgaaagactgacc	atg	None	0	putative lipoprotein	LipoP
**CcrColossus_gp246**	+	tgtccagatcaacggcatcca	atg	None	0	hypothetical conserved protein	
**CcrColossus_gp247**	+	ggacaatggaAGGgtccaaga	atg	agg	8	putative elongation factor EF-Ts domain protein	IPR014039
**CcrColossus_gp248**	+	tagaccacatcGGAGGgccgt	atg	ggagg	5	hypothetical conserved protein	
**CcrColossus_gp249**	+	ctccagcttcttcacctgctc	gtg	None	0	hypothetical novel protein	
**CcrColossus_gp250**	+	ggtcacgttcgAGGAcgtcgc	atg	agga	6	hypothetical novel protein	
**CcrColossus_gp251**	+	cctGGAGGatgagttcgcctg	atg	ggagg	13	hypothetical novel protein	
**CcrColossus_gp252**	+	cacaacagAGGAGtttgtccc	atg	aggag	8	putative HTH domain DNA-binding protein	IPR001387, IPR010982, G3DSA:1.10.260.40
**CcrColossus_gp253**	+	tcagcgcggcaAGGcggcctg	atg	agg	7	hypothetical novel protein	
**CcrColossus_gp254**	+	ctcatggcggtgaagcactaa	ttg	None	0	hypothetical novel protein	
**CcrColossus_gp255**	+	ttgaactgAGGAGtctagctc	ttg	aggag	8	hypothetical conserved protein	
**CcrColossus_gp256**	+	gttcctccgaAGGActaagcc	atg	agga	7	putative ferritin-like protein	IPR012347, IPR003251, IPR009078
**CcrColossus_gp257**	+	tgacgatcttaAGGAGaccct	atg	aggag	5	hypothetical novel protein	
**CcrColossus_gp258**	+	agttgcgGGAGGccgtgggcg	atg	ggagg	9	hypothetical novel protein	
**CcrColossus_gp259**	+	ggacggaaAGGTgttcaaatt	ttg	aggt	9	hypothetical conserved protein	
**CcrColossus_gp260**	+	agtacGGAGcgcgcgtacgtc	atg	ggag	12	hypothetical novel protein	
**CcrColossus_gp261**	+	gacgatcacacGGAGGcaatc	atg	ggagg	5	hypothetical conserved protein	
**CcrColossus_gp262**	+	gatgttcaaGGGGatcgccgc	atg	gggg	8	hypothetical novel protein	
**CcrColossus_gp263**	+	ccatGAGGcgttcgcgttctt	ctg	gagg	13	hypothetical novel protein	
**CcrColossus_gp264**	+	gttcgtgctGGAGGgctgagc	atg	ggagg	7	hypothetical novel protein	
**CcrColossus_gp265**	+	acgatcactgAGGAGGTcccg	atg	aggaggt	4	DUF262 protein	IPR004919
**CcrColossus_gp266**	+	cgagccgaAGGTggtcgcgtg	atg	aggt	9	hypothetical conserved protein	
**CcrColossus_gp267**	+	cagcccggaAGGAGGTcgctg	atg	aggaggt	5	hypothetical novel protein	
**CcrColossus_gp268**	+	tgaacagaAGGAGGcttccgc	atg	aggagg	7	putative dehalogenase hydrolase-like protein	IPR005833, IPR005834, SSF56784, PTHR12725, G3DSA:3.40.50.1000
**CcrColossus_gp269**	+	ccctgaccGGAGtccttctga	atg	ggag	9	putative phosphoribosyltransferase-like domain protein	SSF53271
**CcrColossus_gp270**	+	gtctaactgacAGGTaaaacc	atg	aggt	6	hypothetical novel protein	
**CcrColossus_gp271**	+	gtgacgaagcAGGTcacccgc	atg	aggt	7	hypothetical novel protein	
**CcrColossus_gp272**	+	gaccctttcAGGAtaaccggc	atg	agga	8	hypothetical novel protein	
**CcrColossus_gp273**	+	ctaccaattcacGGAGtcctc	atg	ggag	5	hypothetical novel protein	
**CcrColossus_gp274**	+	atcGAGGTcGAGGTctaggcc	atg	gaggt	7	putative TAT signal protein	IPR006311 ; PRED-TAT
**CcrColossus_gp275**	+	caagctgaAGGgttcggtctg	atg	agg	10	hypothetical conserved protein	
**CcrColossus_gp276**	+	tgaacggccttGGAGaagaca	gtg	ggag	6	hypothetical conserved protein	SSF56784, G3DSA:3.40.50.1000
**CcrColossus_gp277**	+	gtttgatgcgcgggtgcctga	atg	None	0	putative HNH endonuclease	IPR002711
**CcrColossus_gp278**	+	agaaccagacggtggtggcct	atg	None	0	hypothetical novel protein	
**CcrColossus_gp279**	+	ctggaataagGGAGcctaacc	ttg	ggag	7	hypothetical conserved protein	
**CcrColossus_gp280**	+	gttgatcgcggcgaacctctg	atg	None	0	hypothetical conserved protein	
**CcrColossus_gp281**	+	tttcccgaatAGGAGtctgtt	gtg	aggag	6	hypothetical novel protein	
**CcrColossus_gp282**	+	tcgaaccgatcaagtagcgcc	atg	None	0	hypothetical novel protein	
**CcrColossus_gp283**	+	gctttgggtGGAcggctacca	gtg	gga	9	putative radical SAM-like protein	SSF102114, G3DSA:3.80.30.20
**CcrColossus_gp284**	+	cggcatgttcatGGAGGcgtg	atg	ggagg	4	hypothetical novel protein	
**CcrColossus_gp285**	+	cgcctgagatGGAatagaacc	atg	gga	8	hypothetical conserved protein	
**CcrColossus_gp286**	+	tgtacgcgcctGGAGGTgaag	atg	ggaggt	4	hypothetical novel protein	
**CcrColossus_gp287**	+	cgcgcaccggGGAGaatagaa	atg	ggag	7	hypothetical novel protein	
**CcrColossus_gp288**	+	cacgtcagctAGGAGGgcgcc	atg	aggagg	5	hypothetical novel protein	
**CcrColossus_gp289**	+	ccaaactggcAGGTtggcggg	atg	aggt	7	putative tRNA nucleotidyl transferase	IPR002646, PTHR13734, SSF81301, G3DSA:3.30.460.10, G3DSA:1.10.3090.10
**CcrColossus_gp290**	+	gatcgtgatGGAGGaatggac	ttg	ggagg	7	hypothetical novel protein	
**CcrColossus_gp291**	+	cgcctggattGGAGccgccag	atg	ggag	7	hypothetical novel protein	
**CcrColossus_gp292**	+	cgaggcgcGGAGcggcgagtg	atg	ggag	9	hypothetical novel protein	
**CcrColossus_gp293**	+	cggctttGAGGTgttttaggg	atg	gaggt	9	hypothetical novel protein	
**CcrColossus_gp294**	+	caagccgaagaAGGcggcctg	atg	agg	7	hypothetical novel protein	
**CcrColossus_gp295**	+	tcctgggccGGActgcaccgc	atg	gga	9	hypothetical novel protein	
**CcrColossus_gp296**	+	acgatctcaaAGGAaggccct	gtg	agga	7	hypothetical conserved protein	
**CcrColossus_gp297**	+	gagcagcggagcGGAGGaagc	atg	ggagg	4	hypothetical conserved protein	
**CcrColossus_gp298**	+	gatcaatcagaAGGAGGtccc	atg	aggagg	4	hypothetical novel protein	
**CcrColossus_gp299**	+	ggcgccgacgaaAGGAcacca	atg	agga	5	hypothetical novel protein	
**CcrColossus_gp300**	+	cacctcgcgggtGGAcgcctg	atg	gga	6	putative tRNA His guanylyltransferase protein	IPR007537
**CcrColossus_gp301**	+	aacgcatgacAGGTgaagccg	atg	aggt	7	hypothetical novel protein	
**CcrColossus_gp302**	+	ccaaacttgtAGGAGGTccag	atg	aggaggt	4	hypothetical novel protein	
**CcrColossus_gp303**	+	gccgatggtcGAGGccgccgc	atg	gagg	7	hypothetical novel protein	
**CcrColossus_gp304**	+	tgccgtGGAtcaagacgacga	atg	gga	12	hypothetical novel protein	

**CcrColossus_gp305**	+	gggcctGGAGtggcatccgta	gtg	ggag	11	hypothetical novel protein	
**CcrColossus_gp306**	+	tactgacgacaAGGAGGcccc	atg	aggagg	4	hypothetical novel protein	
**CcrColossus_gp307**	+	cgccaagaAGGAcgccgcctg	atg	agga	9	hypothetical conserved protein	
**CcrColossus_gp308**	+	cggcgtGGAGGaacgctttta	atg	ggagg	10	hypothetical novel protein	
**CcrColossus_gp309**	+	caagctggcgatGGGGcgctg	atg	gggg	5	hypothetical novel protein	
**CcrColossus_gp310**	+	tacgatcgcacGGAGacgctg	atg	ggag	6	hypothetical novel protein	
**CcrColossus_gp311**	+	ttcGGAGcgccggcgccccga	atg	ggag	14	hypothetical novel protein	
**CcrColossus_gp312**	+	accgaAGGAccgcgtcagcct	gtg	agga	12	hypothetical novel protein	
**CcrColossus_gp313**	+	aagggctttcGAGGcgaagaa	gtg	gagg	7	hypothetical novel protein	
**CcrColossus_gp314**	+	gcaattactggcagaccagca	gtg	None	0	hypothetical novel protein	
**CcrColossus_gp315**	+	ataaacatgaagGGAGcccac	atg	ggag	5	hypothetical novel protein	
**CcrColossus_gp316**	+	ggtctcgcttatccaagagcc	atg	None	0	hypothetical novel protein	
**CcrColossus_gp317**	+	gatcaatcagAGGAGacccac	atg	aggag	6	hypothetical novel protein	
**CcrColossus_gp318**	+	tcctggtctcggtcgactgac	gtg	None	0	hypothetical novel protein	
**CcrColossus_gp319**	+	tgtgcaatccAGGAGGacccg	atg	aggagg	5	hypothetical novel protein	
**CcrColossus_gp320**	+	caacgccatGGAGGctttctc	atg	ggagg	7	hypothetical novel protein	
**CcrColossus_gp321**	+	ccggtcAGGTcggcgtctacg	atg	aggt	11	hypothetical novel protein	
**CcrColossus_gp322**	+	cggcaccctgccgctcgccgc	gtg	None	0	hypothetical novel protein	
**CcrColossus_gp323**	+	cgacgaaacgAGGAGGcaccc	atg	aggagg	5	hypothetical novel protein	
**CcrColossus_gp324**	+	cgtcgcgcctaAGGAccctcc	atg	agga	6	hypothetical conserved protein	
**CcrColossus_gp325**	+	cctttctggaaAGGAGctaat	atg	aggag	5	hypothetical novel protein	
**CcrColossus_gp326**	+	tcgcacgctGGAGcgtgcctg	atg	ggag	8	hypothetical novel protein	
**CcrColossus_gp327**	+	ccgcaatttgAGGAGGcctga	atg	aggagg	5	hypothetical novel protein	
**CcrColossus_gp328**	+	tcagcaaAGGAGtcacatccc	atg	aggag	9	hypothetical conserved protein	
**CcrColossus_gp329**	+	cgtcaagaccggcgcgctctg	atg	None	0	hypothetical novel protein	
**CcrColossus_gp330**	+	ctcctgccggcggcggcctaa	atg	None	0	putative methyltransferase	SSF53335, G3DSA:3.40.50.150
**CcrColossus_gp331**	+	cctgatcaaaAGGAGccgctg	gtg	aggag	6	hypothetical novel protein	
**CcrColossus_gp332**	+	cgtcctggcggcgctctgcct	atg	None	0	hypothetical novel protein	
**CcrColossus_gp333**	+	ctccaacccaaAGGAGccctc	atg	aggag	5	hypothetical conserved protein	
**CcrColossus_gp334**	+	gcgcggaagaaGAGctaaacc	atg	gag	7	hypothetical conserved protein	
**CcrColossus_gp335**	+	ctcggtgatggcgaccgtcgg	atg	None	0	hypothetical novel protein	
**CcrColossus_gp336**	+	tagcccgtactAGGAGGaccg	atg	aggagg	4	hypothetical novel protein	
**CcrColossus_gp337**	+	tcccgacGGAGccgggctgac	atg	ggag	10	hypothetical novel protein	
**CcrColossus_gp338**	+	atcacagccatGGAGGctcct	ttg	ggagg	5	hypothetical conserved protein	
**CcrColossus_gp339**	+	cttcaaggGGAGcttctcctg	atg	ggag	9	hypothetical novel protein	
**CcrColossus_gp340**	+	caacctgaacctgccattctg	atg	None	0	hypothetical conserved protein	
**CcrColossus_gp341**	+	cgatcaatgAGGAGGTaggtc	atg	aggaggt	5	hypothetical novel protein	
**CcrColossus_gp342**	+	tttcgcactgaagGGAGccta	gtg	ggag	4	hypothetical novel protein	
**CcrColossus_gp343**	+	cttcttccgaaagatcaactg	atg	None	0	hypothetical novel protein	
**CcrColossus_gp344**	+	cattcgcaaAGGAaagcgcgc	atg	agga	8	hypothetical conserved protein	
**CcrColossus_gp345**	+	acgatcacaaAGGAGaccctc	atg	aggag	6	hypothetical novel protein	
**CcrColossus_gp346**	+	gttcatgcGGAcgtagctccg	atg	gga	10	hypothetical conserved protein	
**CcrColossus_gp347**	+	cgccgcgaAGGAGttggcgca	atg	aggag	8	hypothetical novel protein	
**CcrColossus_gp348**	+	cgctcagatcatGGGGatcgc	atg	gggg	5	hypothetical novel protein	
**CcrColossus_gp349**	+	ccAGGcgaacAGGccgccggc	atg	agg	8	hypothetical novel protein	
**CcrColossus_gp350**	+	atttgcgccggttcGAGtctg	atg	gag	4	hypothetical novel protein	
**CcrColossus_gp351**	+	gccaacctgaaAGGctgaacc	atg	agg	7	hypothetical conserved protein	
**CcrColossus_gp352**	+	GGAaccctatgGGAtgtcgaa	gtg	gga	7	hypothetical conserved protein	
**CcrColossus_gp353**	+	tatgaccttgaGAGGgatgtg	atg	gagg	6	hypothetical conserved protein	
**CcrColossus_gp354**	+	tcagaagctgaagttcatgtg	atg	None	0	hypothetical novel protein	
**CcrColossus_gp355**	+	cctcgcGGAGaagaaacgtcg	atg	ggag	11	hypothetical novel protein	
**CcrColossus_gp356**	+	cgctaaaggGGAGGggctaag	atg	ggagg	7	hypothetical conserved protein	
**CcrColossus_gp357**	+	cggtaAGGAGtacaagtacac	gtg	aggag	11	hypothetical novel protein	
**CcrColossus_gp358**	+	tcacagcacAGGAGGcctctc	atg	aggagg	6	putative rRNA methyltransferase	IPR004398, SSF53335, G3DSA:3.40.50.150
**CcrColossus_gp359**	+	tcatcacgctgtGGAagccgc	atg	gga	6	hypothetical novel protein	
**CcrColossus_gp360**	+	tacctGGAGGacggcacggtc	atg	ggagg	11	hypothetical novel protein	
**CcrColossus_gp361**	+	gttagccaacGGAGactaccc	atg	ggag	7	hypothetical novel protein	
**CcrColossus_gp362**	+	cccgattttgtcAGGcgtgag	atg	agg	6	hypothetical novel protein	
**CcrColossus_gp363**	-	tcccgaggcctGGAGatcacc	atg	ggag	6	hypothetical novel protein	
**CcrColossus_gp364**	-	gaacatgctGGAGGgcgcgga	gtg	ggagg	7	hypothetical novel protein	
**CcrColossus_gp365**	-	cgaactggccGAGGTgtacct	gtg	gaggt	6	hypothetical novel protein	
**CcrColossus_gp366**	-	tgaagatgcGGAGcgcttcta	atg	ggag	8	hypothetical novel protein	
**CcrColossus_gp367**	-	ggcgcGAGGgaaggctgccta	atg	gagg	12	hypothetical novel protein	
**CcrColossus_gp368**	-	cgtgaAGGTcgtggccggcga	ctg	aggt	12	hypothetical novel protein	
**CcrColossus_gp369**	-	aacaacgaaAGGAGGctcgcc	atg	aggagg	6	hypothetical novel protein	
**CcrColossus_gp370**	-	gctctatgagaAGGAGGcctc	atg	aggagg	4	hypothetical novel protein	
**CcrColossus_gp371**	-	cGAGccggtggtgacggcgcc	atg	gag	17	hypothetical conserved protein	
**CcrColossus_gp372**	-	atacatcgtctAGGAGGgctc	atg	aggagg	4	hypothetical conserved protein	
**CcrColossus_gp373**	-	tcaacgccaagaaagaagcca	atg	None	0	putative NAD GMP synthase domain protein	IPR014729, IPR022310
**CcrColossus_gp374**	-	ccaaaacGGGGaagcgatctg	atg	gggg	10	hypothetical novel protein	
**CcrColossus_gp375**	-	tgacgatcacgAGGAGGccct	atg	aggagg	4	hypothetical conserved protein	
**CcrColossus_gp376**	-	ggccggcgcGGAGGccgcgtg	atg	ggagg	7	hypothetical novel protein	
**CcrColossus_gp377**	-	cgaacaacccAGGAGGccctg	atg	aggagg	5	hypothetical novel protein	
**CcrColossus_gp378**	-	caagaAGGAcacggtgtcgcg	atg	agga	12	hypothetical novel protein	
**CcrColossus_gp379**	-	gcgatcgccAGGAGccacgcc	atg	aggag	7	hypothetical novel protein	
**CcrColossus_gp380**	+	ccattaaccaatGAGGccgac	atg	gagg	5	hypothetical novel protein	
**CcrColossus_gp381**	-	cccgcAGGAGGgaaacgccta	atg	aggagg	10	hypothetical novel protein	

**CcrColossus_gp382**	-	cgcgacgcgcctGGAcgactg	atg	gga	6	hypothetical novel protein	
**CcrColossus_gp383**	-	gtggcgtgacAGGAGGcttat	atg	aggagg	5	hypothetical novel protein	
**CcrColossus_gp384**	-	ctgccgacGGAGttctgacgc	atg	ggag	9	hypothetical novel protein	
**CcrColossus_gp385**	-	acagcgaaatAGGAGatcagg	atg	aggag	6	hypothetical conserved protein	
**CcrColossus_gp386**	-	tggacgaacacAGGAGGccct	atg	aggagg	4	hypothetical novel protein	
**CcrColossus_gp387**	+	aaaaacggctattccacctac	atg	None	0	hypothetical novel protein	
**CcrColossus_gp388**	+	caaatggcttgaAGGTaaacc	atg	aggt	5	hypothetical novel protein	
**CcrColossus_gp389**	+	taagtcgtggtgGGAcctctg	atg	gga	6	hypothetical novel protein	
**CcrColossus_gp390**	+	ggtggcgccggtGGActccca	atg	gga	6	hypothetical novel protein	
**CcrColossus_gp391**	+	cgaggcgcGGAGcggcgagtg	atg	ggag	9	hypothetical conserved protein	
**CcrColossus_gp392**	+	cctatttGGAcGGAaacgcac	atg	gga	7	hypothetical conserved protein	
**CcrColossus_gp393**	+	ttttgaagaAGGctcaaacct	gtg	agg	9	hypothetical novel protein	
**CcrColossus_gp394**	+	ctcctgcccacAGGAcggcca	atg	agga	6	hypothetical novel protein	
**CcrColossus_gp395**	+	ccgAGGAGatgaccgagaccg	atg	aggag	13	hypothetical novel protein	
**CcrColossus_gp396**	+	ctgaaggctGAGtccgcttag	atg	gag	9	hypothetical novel protein	
**CcrColossus_gp397**	+	ctcctgctgaaGAGtaccacg	atg	gag	7	hypothetical conserved protein	
**CcrColossus_gp398**	+	tcttcgacaactGAGGgtaca	atg	gagg	5	hypothetical novel protein	
**CcrColossus_gp399**	+	caccatcgtcacggtggagct	atg	None	0	hypothetical novel protein	
**CcrColossus_gp400**	+	ccttcctGGAGtattgagccg	atg	ggag	10	hypothetical novel protein	
**CcrColossus_gp401**	-	tctagccagGGAacaacccgc	atg	gga	9	hypothetical novel protein	
**CcrColossus_gp402**	-	acgtggtcgaacAGGAaggcg	atg	agga	5	hypothetical novel protein	
**CcrColossus_gp403**	-	cgtgaccgtcGAGGTggtctc	gtg	gaggt	6	hypothetical novel protein	
**CcrColossus_gp404**	-	gattaagccggAGGAGGcatg	atg	aggagg	4	hypothetical novel protein	
**CcrColossus_gp405**	-	cgcgctcatgatGGActgctg	atg	gga	6	hypothetical conserved protein	
**CcrColossus_gp406**	-	gctgaagcAGGcggctccgca	atg	agg	10	hypothetical novel protein	
**CcrColossus_gp407**	-	ccgcctgtcGGAGatcacgaa	gtg	ggag	8	hypothetical novel protein	
**CcrColossus_gp408**	-	ccttgccctGGAGGccgccgc	atg	ggagg	7	hypothetical novel protein	
**CcrColossus_gp409**	-	cctgctagaAGGTcaaggctg	atg	aggt	8	hypothetical novel protein	
**CcrColossus_gp410**	-	cggagagaAGGAcccacgctg	gtg	agga	9	hypothetical novel protein	
**CcrColossus_gp411**	-	gacgatcaagAGGAGaccccg	atg	aggag	6	hypothetical novel protein	
**CcrColossus_gp412**	-	caagaaggaacGAGGcctcgc	atg	gagg	6	hypothetical novel protein	
**CcrColossus_gp413**	-	ccgcgctgacGGAGGaaggca	atg	ggagg	6	hypothetical novel protein	
**CcrColossus_gp414**	-	ctacgtgcgaaAGGAaccctg	atg	agga	6	hypothetical novel protein	
**CcrColossus_gp415**	-	tgctgggcctGGAGGaattca	ttg	ggagg	6	hypothetical novel protein	
**CcrColossus_gp416**	-	gacgatcactAGGAGaacctg	atg	aggag	6	hypothetical novel protein	
**CcrColossus_gp417**	-	tcgacgcGAGGcgcggctgaa	gtg	gagg	10	hypothetical conserved protein	PF09356
**CcrColossus_gp418**	-	gtatgttctGGAGaaataacg	atg	ggag	8	hypothetical novel protein	
**CcrColossus_gp419**	-	cgtgacgccggtggtgctgcc	gtg	None	0	hypothetical novel protein	
**CcrColossus_gp420**	-	cgccggcacaagGGAatcatg	atg	gga	6	hypothetical novel protein	
**CcrColossus_gp421**	-	ttcccgaAGGAGGcctgatcc	atg	aggagg	8	hypothetical novel protein	
**CcrColossus_gp422**	-	cgacgaagcaAGGAGacccgg	atg	aggag	6	hypothetical novel protein	
**CcrColossus_gp423**	-	gattaatcaAGGAGGgctccg	atg	aggagg	6	hypothetical novel protein	
**CcrColossus_gp424**	-	caccctggagaAGGAagcctg	atg	agga	6	putative HTH domain DNA-binding protein	IPR001387, IPR010982, PTHR10245, G3DSA:1.10.260.40
**CcrColossus_gp425**	-	gtaccttgcgGGAGaagaagc	gtg	ggag	7	hypothetical novel protein	
**CcrColossus_gp426**	-	gctggtctGGAagcgcgagca	atg	gga	10	hypothetical novel protein	
**CcrColossus_gp427**	-	actcgctcccAGGAGGccccg	atg	aggagg	5	hypothetical novel protein	
**CcrColossus_gp428**	-	ccggctgtacGAGGTcgagtg	atg	gaggt	6	hypothetical novel protein	
**CcrColossus_gp429**	-	ccctaactccacgAGGAcatg	atg	agga	4	hypothetical novel protein	
**CcrColossus_gp430**	-	cgttgcgaatgaAGGTgtgcg	atg	aggt	5	hypothetical novel protein	
**CcrColossus_gp431**	-	cgatctgcgGGAGatgatcgg	atg	ggag	8	hypothetical novel protein	
**CcrColossus_gp432**	-	gccgatcgtgttgaagcgccg	atg	None	0	hypothetical novel protein	
**CcrColossus_gp433**	-	ggtcgccgtGAGcgtgcgctg	atg	gag	9	hypothetical novel protein	
**CcrColossus_gp434**	-	taacttcaacaAGGAGGcacc	atg	aggagg	4	hypothetical novel protein	
**CcrColossus_gp435**	-	gacgatcaataAGGAGacccc	atg	aggag	5	hypothetical novel protein	
**CcrColossus_gp436**	-	gtggcggtccaAGGcgcaatc	atg	agg	7	hypothetical novel protein	
**CcrColossus_gp437**	-	gacggaagatcGAGtgctgcg	ctg	gag	7	hypothetical novel protein	
**CcrColossus_gp438**	-	agcgtaggctAGGAGGggttc	atg	aggagg	5	hypothetical novel protein	
**CcrColossus_gp439**	-	cgggatgcctAGGcggttgtc	atg	agg	8	hypothetical novel protein	
**CcrColossus_gp440**	+	aactccgaccAGGAttaaccg	atg	agga	7	hypothetical conserved protein	
**CcrColossus_gp441**	+	cagttaacgctttcAGGtccc	atg	agg	4	hypothetical conserved protein	
**CcrColossus_gp442**	+	ggcggccaacGGAGccgcgtg	atg	ggag	7	hypothetical novel protein	
**CcrColossus_gp443**	+	ccgcgaagAGGAGGctgcgca	atg	aggagg	7	hypothetical novel protein	
**CcrColossus_gp444**	+	tgtcttctggttgtatcactg	atg	None	0	putative RNaseH-like domain protein	IPR012337
**CcrColossus_gp445**	+	tatcctggtGGAGttccctaa	ttg	ggag	8	hypothetical conserved protein	
**CcrColossus_gp446**	+	cgccggcccGGAcaagacgcc	atg	gga	9	hypothetical novel protein	
**CcrColossus_gp447**	+	gccctagcgaagtaaaagaca	ttg	None	0	putative terminase small subunit	HHpred vs pdb70_18Aug12, hit 3zqp_A, prob 97.8% E=8.3E-06; position relative to terminase large subunit, see text
**CcrColossus_gp448**	+	cggcgacgacgaataatcacc	atg	None	0	putative terminase large subunit	IPR004921, IPR003587, IPR006141, IPR006517, IPR004042, SSF51294, G3DSA:2.170.16.10, G3DSA:3.10.28.10
